# Local bandgap and optoelectronic measurement using monochromated STEM-VEELS: fundamentals, challenges, and recent advances

**DOI:** 10.1186/s42649-026-00143-9

**Published:** 2026-08-28

**Authors:** Hee Joon Jung

**Affiliations:** Korea Research Institute of Standards and Science, Daejeon, 34113 Republic of Korea

**Keywords:** Monochromated STEM-VEELS, Local bandgap, Spatial delocalization, Dielectric function, Valence electron energy loss spectroscopy (VEELS)

## Abstract

Optoelectronic characterization of local bandgap, dielectric response, and plasmon at the nanometer scale has become increasingly important for understanding and optimizing modern electronic, optoelectronic, and quantum materials. Conventional optical techniques generally provide spatially averaged information and are therefore limited in their ability to resolve localized electronic variations arising from defects, interfaces, strain fields, and quantum confinement. To overcome these limits, monochromated scanning transmission electron microscopy–valence electron energy loss spectroscopy (STEM-VEELS) has evolved from a qualitative low-loss spectroscopy technique into a quantitative nanoscale bandgap metrology platform. By analyzing low-loss inelastic scattering signals with a sub-nanometer electron probe and sub-100 meV energy resolution, STEM-VEELS enables the direct investigation of bandgap onsets, local density of states (LDOS), and interband transitions at the nanoscale, although the effective spatial resolution of these measurements can be substantially broader than the probe size because of inelastic-scattering delocalization.

This review summarizes the fundamental principles and recent advances in monochromated STEM-VEELS for local bandgap measurement. Particular emphasis is placed on the physical origin of low-loss excitations, addressing critical challenges such as momentum transfer and spatial delocalization. Practical experimental strategies for rigorous zero-loss peak (ZLP) subtraction and bandgap onset determination are systematically discussed. Furthermore, major analytical artifacts—such as Cherenkov radiation, guided optical modes, and beam-induced damage—are critically reviewed alongside state-of-the-art mitigation strategies.

Recent developments in off-axis and Bessel-aperture STEM-VEELS geometries, machine-learning-assisted spectral analysis, automated onset extraction, and uncertainty-aware bandgap mapping are highlighted. Representative applications involving quantum dots, wide-bandgap oxides, heterointerfaces, and two-dimensional materials demonstrate the unparalleled capability of STEM-VEELS to resolve spatially varying electronic structures. Finally, future perspectives, including operando spectroscopy, ultrafast VEELS, artificial intelligence-driven analysis, and SEM-REELS, are discussed, providing a robust framework for standardization and quantitative nanoscale bandgap metrology.

## Introduction

The bandgap (*E*_g_) is a fundamental physical property that governs the electronic, optical, and transport properties of semiconductor and insulating materials, ultimately determining their performance in real-world applications such as light-emitting diodes, photovoltaics, power electronics, and sensors. In conventional bulk materials, the bandgap is generally uniform and well-understood. However, as modern solid-state devices and functional nanomaterials shrink toward the atomic scale, their electronic structures rarely remain homogeneous. In low-dimensional systems—such as 0-dimensional (0D) quantum dots (Jung et al. 2013), 2-dimensional (2D) moiré superlattices (Li et al. 2014; Komsa and Krasheninnikov 2012; Chiu et al. 2015), and complex oxide heterointerfaces (Huang et al. 2014)—the local bandgap fluctuates dramatically at the nanometer scale. These spatial *E*_g_ variations are driven by diverse nanoscale phenomena, including size-dependent quantum confinement, localized strain gradients, interfacial charge transfer, and atomic defect states. Consequently, accurately measuring and mapping these spatial *E*_g_ variations is no longer merely an observational characterization of material properties; it has become a critical metrological requirement for designing next-generation optoelectronics, valleytronics, and energy-conversion devices.

A variety of experimental techniques have been developed for bandgap determination and electronic structure characterization, each providing complementary information depending on the spatial scale, excitation mechanism, and probing depth, as summarized in Table 1. Among the most widely used approaches, ultraviolet–visible (UV–Vis) absorption spectroscopy remains a standard method for determining optical bandgaps through analysis of absorption edges and transition energies. Owing to its simplicity, quantitative nature, and excellent energy resolution, UV–Vis spectroscopy has become a routine characterization tool for semiconductors and dielectric materials. However, the technique inherently provides spatially averaged information over relatively large sampling volumes and therefore cannot resolve local bandgap variations arising from nanoscale compositional fluctuations, interfaces, or defects.

**Table 1:** Comparison of representative techniques for bandgap measurement

Method	Monochromated STEM-VEELS	UV–Vis Absorption	Photo-luminescence (PL)	Cathodo-luminescence (CL)	Spectroscopic Ellipsometry	XPS/UPS	Scanning Tunneling Spectroscopy (STS)
Spatial Resolution	1–10 nm (potentially atomic scale)	10–1000 μm	0.5–10 μm	10–100 nm	mm scale	1–100 μm (conventional) 50 nm–1 μm (state-of-the-art)	Atomic scale
Energy Resolution	10–100 meV	< 10 meV	< 1 meV	1–10 meV	1–10 meV	50–500 meV	10–100 meV
Surface Sensitivity	Moderate	Low	Moderate	High	Low	Very high	Extremely high
Bulk Sensitivity	High	High	Moderate	Moderate	High	Limited	Very limited
Buried Interface Accessibility	Excellent	Poor	Poor	Moderate	Poor	Limited	Not accessible
Local Bandgap Mapping	Excellent	Not available	Limited	Good	Not available	Not available	Excellent
Plasmon Characterization	Excellent	Good	Not available	Limited	Good	Not available	Limited
Exciton Analysis	Good	Limited	Excellent	Excellent	Limited	Not available	Limited
Detection of Non-radiative Transitions	Yes	No	No	No	No	No	Yes
Dielectric Function Analysis	Yes	Partial	No	No	Excellent	No	No
Local Density of States (LDOS)	Partial	No	No	No	No	Partial	Excellent
In situ / Operando	Excellent (biasing, heating, gas/liquid-cell TEM)	Moderate	Excellent	Good	Limited	Moderate	Poor
Typical Sample Requirements	Electron-transparent TEM specimen (< 100 nm)	Transparent thin film or bulk sample	Optically active material	Electron-beam stable specimen	Flat reflective surface	Vacuum-compatible surface	Conductive, atomically clean surface
Information Obtained	Bandgap, dielectric response, plasmon excitations, LDOS, interband transitions	Optical bandgap, absorption coefficient	Exciton emission, recombination dynamics, defect states	Luminescence, defects, impurity levels	Optical constants (n, k), dielectric function	Valence-band position, work function, chemical states	Atomic-scale electronic states and LDOS
Major Advantages	Simultaneous structural and spectroscopic analysis; highest spatial resolution; interface, defect, and buried-layer characterization	Simple, quantitative, widely available	Extremely high energy resolution; highly sensitive to optical quality	Higher spatial resolution than PL; compatible with SEM/TEM platforms	Accurate determination of optical constants and dielectric properties	Direct electronic structure and chemical-state information	Ultimate spatial resolution and direct LDOS measurement
Major Limitations	Beam damage, ZLP subtraction, delocalization effects, demanding specimen preparation	Poor spatial resolution; bulk-averaged information	Radiative transitions only; limited applicability to indirect-gap materials	Carrier diffusion limits true spatial resolution	No local information; requires optical modeling	Highly surface sensitive; limited bulk information	Conductive samples required; surface-only technique

Photoluminescence (PL) spectroscopy offers significantly higher sensitivity to excitonic transitions, defect-related emission states, and carrier recombination processes. Because PL directly probes radiative electronic transitions, it provides valuable insight into optical quality and defect populations in semiconductors. Nevertheless, PL measurements are fundamentally restricted to radiative processes and cannot directly access non-radiative electronic excitations, which often dominate the electronic behavior of complex materials and device structures. Furthermore, the spatial resolution of conventional PL remains limited by optical diffraction.

Cathodoluminescence (CL) spectroscopy partially overcomes the spatial-resolution limitations of PL by employing an electron beam as the excitation source. This enables nanoscale mapping of luminescence properties, making CL particularly useful for investigating defects, impurities, and local recombination centers in semiconductors. However, the measured signal still originates from radiative recombination processes and is often broadened by carrier diffusion, limiting the true spatial resolution of electronic-structure analysis.

Spectroscopic ellipsometry represents another powerful optical technique that can determine complex dielectric functions and optical constants with high accuracy. Through model-based analysis of reflected polarized light, ellipsometry provides detailed information on optical transitions and dielectric responses over a broad energy range. However, the technique remains inherently macroscopic and lacks the ability to directly probe local electronic variations at interfaces or defects.

Photoelectron spectroscopies, including X-ray photoelectron spectroscopy (XPS) and ultraviolet photoelectron spectroscopy (UPS), provide complementary information on valence-band positions, work functions, and chemical bonding environments. These methods are highly valuable for band alignment studies and surface electronic structure analysis. Conventional laboratory-scale XPS/UPS typically provides a spatial resolution on the micrometer scale (~ 1–100 μm), depending on the source, focusing optics, and instrumental configuration. State-of-the-art photoemission techniques, however, have substantially improved the achievable spatial resolution into the nanometer regime. For instance, synchrotron-based scanning photoelectron microscopy (3D nano-ESCA) has demonstrated a spatial resolution below 70 nm (Horiba et al. 2011), while energy-filtered X-ray photoemission electron microscopy (XPEEM/NanoESCA) can provide chemical and work-function mapping with a lateral resolution below 50 nm (Escher et al. 2005; Liu et al. 2022). Notably, these highest spatial resolutions have primarily been demonstrated for photoemission spectroscopy, chemical-state analysis, and work-function mapping rather than for direct bandgap measurements. Thus, the spatial resolution specifically demonstrated for bandgap determination using XPS/UPS remains more limited than the best spatial resolution achieved by state-of-the-art photoemission techniques. In addition, their applicability to local bandgap characterization remains constrained by their strong surface sensitivity and lower spatial resolution compared with modern electron microscopy techniques.

At the opposite extreme, scanning tunneling spectroscopy (STS) can achieve atomic-scale spatial resolution while directly probing the local density of electronic states. STS has become a powerful tool for investigating surface electronic structures, localized defect states, and atomic-scale bandgap variations. Nevertheless, its application is generally restricted to conductive, atomically clean surfaces and does not readily provide access to buried interfaces or internal device structures.

Compared with these established approaches, monochromated STEM-VEELS uniquely combines nanometer-scale spatial resolution with high energy resolution while simultaneously providing structural, chemical, and spectroscopic information from the same region of interest. As summarized in Table 2, STEM-VEELS offers capabilities that are difficult or inaccessible with most competing techniques, including local bandgap mapping, buried-interface analysis, detection of non-radiative electronic excitations, dielectric function extraction, and defect-scale electronic-structure characterization. Moreover, the ability to directly correlate spectroscopic signatures with atomic-scale structural information acquired using the STEM platform provides a distinct advantage for investigating complex heterostructures, quantum materials, and advanced semiconductor devices. These unique capabilities have established monochromated STEM-VEELS as one of the most versatile approaches for probing localized electronic excitations and bandgap variations at the nanoscale (Egerton 2011; Batson et al. 1986).

**Table 2:** Key advantages of STEM-VEELS vs. other bandgap characterization techniques

Key Capability	STEM-VEELS	Other Techniques
Local bandgap mapping	✓	Limited
Buried interface analysis	✓	Limited
Non-radiative transitions	✓	Mostly unavailable
Dielectric function extraction	✓	Limited
Defect-scale analysis	✓	Limited
Simultaneous structural imaging	✓	No (only STS possible from surface)
Atomic-column correlation	Potentially ✓	No

Importantly, the information accessible by monochromated STEM-VEELS extends far beyond local bandgap determination. As summarized in Table 3, the low-loss region of the EELS spectrum contains signatures of a broad range of elementary electronic excitations, each providing complementary information on the optical and electronic behavior of materials. The onset of interband transitions is directly related to the local bandgap and electronic density of states, forming the basis for nanoscale *E*_g_ measurements (Egerton 2011; Zhang et al. 2008; Cheynet et al. 2007). In addition, excitonic excitations arising from Coulomb-bound electron–hole pairs provide valuable information on many-body interactions, dielectric screening, and quantum confinement effects, particularly in low-dimensional semiconductors and van der Waals heterostructures (Susarla et al. 2021).

**Table 3:** Electronic excitations accessible by monochromated STEM-VEELS beyond local bandgap

Excitation Type	Information	Typical Energy Range	Relevance
Interband transition	Local bandgap	1–10 eV	Direct
Exciton	Bound electron-hole states	Near *E*_g_	Optical properties
Surface plasmon	Surface electron oscillation	1–20 eV	Nano-optics
Bulk plasmon	Collective valence excitation	5–30 eV	Electron density
Guided optical mode	Waveguide excitation	Material dependent	Thin films
Cherenkov radiation	Radiative loss process	Low-loss region	Artifact
Dielectric response	ε₁(E), ε₂(E)	Broad range	Optical constants

Beyond single-particle excitations, STEM-VEELS is highly sensitive to collective electronic excitations. Bulk plasmons reflect the average valence electron density and electronic structure of a material, whereas surface plasmons provide insight into localized electromagnetic fields, dielectric environments, and nanoscale optical confinement (Egerton 2011; Stöger-Pollach 2008). The low-loss spectrum may also contain guided optical modes, polaritonic excitations, and interface-related resonances that emerge in thin films, multilayers, and nanostructured materials (Stöger-Pollach 2008; Korneychuk et al. 2018a, b; Li et al. 2018). Furthermore, quantitative analysis of the low-loss signal via Kramers–Kronig transformations enables reconstruction of the complex dielectric function, allowing extraction of fundamental optical properties, including the real and imaginary dielectric responses (Egerton 2011; Cheynet et al. 2007).

At the same time, several additional spectral contributions, such as Cherenkov radiation and guided optical modes, can appear within the same energy-loss range and significantly influence bandgap determination if not properly identified and removed (Kröger 1970; Stöger-Pollach 2008; Korneychuk et al. 2018a, b). Consequently, accurate interpretation of monochromated STEM-VEELS requires a comprehensive understanding of the various excitation processes summarized in Table 3. This broad excitation sensitivity distinguishes STEM-VEELS from conventional bandgap characterization techniques and establishes it as a versatile nanoscale optical spectroscopy platform capable of probing electronic, optical, and dielectric phenomena simultaneously.

Advances in nano-optics and electron spectroscopy have further expanded the scope of STEM-VEELS beyond conventional electronic-structure characterization. Modern low-loss EELS is increasingly recognized as a powerful nanoscale optical spectroscopy technique capable of probing localized surface plasmons, excitonic resonances, dielectric responses, and light–matter interactions with nanometer-scale spatial resolution (Wu et al. 2018). Ultra-high-energy-resolution STEM-EELS has enabled the direct visualization of quantum plasmon resonances in individual metallic nanoparticles, revealing quantum-size effects and spatially localized optical excitations that are inaccessible to conventional optical spectroscopies (Scholl et al. 2012). Furthermore, the emergence of photon-assisted electron spectroscopies, including resonant electron energy-gain and energy-loss spectroscopy, has opened new opportunities to probe light–matter interactions at the nanoscale with unprecedented spatial resolution (Liu et al. 2019). These developments highlight the growing role of monochromated STEM-VEELS as a comprehensive platform for investigating local bandgaps, excitonic phenomena, plasmonic excitations, dielectric responses, and nanoscale optical physics within a unified experimental framework.

To overcome this fundamental spatial limitation, scanning transmission electron microscopy combined with valence-electron energy-loss spectroscopy (STEM-VEELS) has emerged as an indispensable high-resolution analytical platform. Operating in the low-loss energy regime (typically 0–100 eV), STEM-VEELS probes inelastic scattering events that occur when fast incident electrons interact with valence electrons in the specimen. The onset of this low-loss inelastic signal directly correlates with the optical gap and the local joint density of states (JDOS).

**Fig. 1:**
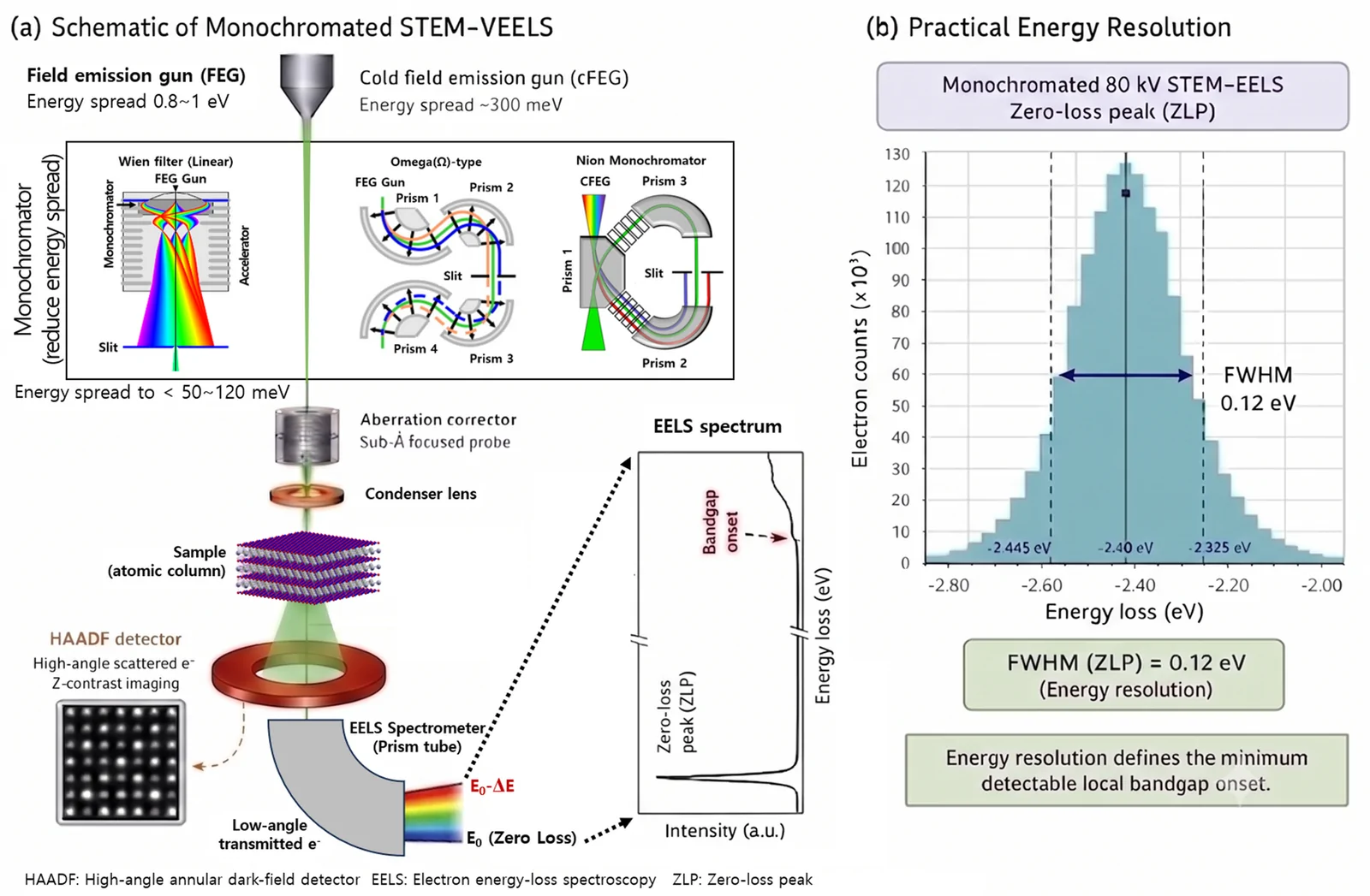
(**a**) Schematic illustration of a monochromated STEM-VEELS configuration. The electron beam generated by a general cold field emission gun (FEG) or cold field emission gun (cFEG) passes through a monochromator (e.g., a Wien filter, Omega-type, or Nion Monochromator), reducing the initial energy spread to < 50 ~ 120 meV before interacting with the sample. The high-angle scattered electrons form a Z-contrast image, while the low-angle transmitted electrons are dispersed by the magnetic prism for energy-loss analysis. (**b**) Zero-loss peak (ZLP) spectrum acquired under monochromated 80 kV STEM-EELS conditions. The practical energy resolution, defined as the full width at half maximum (FWHM) of the ZLP, is approximately 0.12 eV. This ultra-high energy resolution enables the direct detection of narrow-bandgap onsets and is essential for precise *E*_g_ extraction in low-dimensional materials. Panel (**b**) is reproduced from Ref. (Jung et al. 2013)

Despite its theoretical advantages, extracting a reliable local bandgap from a VEELS spectrum has historically been challenging due to the intense zero-loss peak (ZLP)—comprising elastically scattered and unscattered electrons—which obscures the much weaker, low-energy interband transition signals. The transformative breakthrough in this field over the past two decades has been the advent and commercialization of the electron monochromator. Monochromators used in STEM-EELS systems are commonly categorized into Wien-filter-type and Omega-type geometries as shown in Fig. 1a. Wien-filter monochromators utilize crossed electric and magnetic fields to selectively transmit electrons with a narrow energy distribution, whereas Omega-type monochromators employ multiple magnetic prisms and energy-selective slits along an Ω-shaped trajectory to achieve high energy resolution. Unlike conventional cold field-emission guns (cFEG) that typically yield an energy spread of ~ 300 meV, these monochromators selectively filter the electron beam before it interacts with the sample, narrowing the energy spread to below 50 meV.

This ultra-high energy resolution is perfectly preserved as the beam passes through aberration correctors, forming a sub-angstrom probe. After interacting with the sample, high-angle scattered electrons form a Z-contrast image on the high-angle annular dark-field (HAADF) detector, while the low-angle transmitted electrons enter the magnetic prism of the EELS spectrometer, where they are dispersed by energy loss (Δ*E*). The practical impact of this monochromated setup is demonstrated by an energy resolution of approximately 0.12 eV (defined as the full width at half maximum, FWHM, of the ZLP) at an accelerating voltage of 80 kV (Jung et al. 2013) in Fig. 1b. This level of energy resolution is the fundamental prerequisite for quantitative nanoscale bandgap metrology, enabling the unambiguous extraction of subtle onset variations even in narrow-bandgap materials and deeply confined nanostructures.

Building upon this advanced instrumental foundation, modern STEM-VEELS is rapidly evolving from a qualitative, low-loss spectroscopy technique into a rigorous, momentum-sensitive local-excitation metrology platform. However, achieving accurate local bandgap measurements involves profound physical and experimental challenges. Unlike core-loss EELS, low-loss valence excitations are governed by dipole selection rules, leading to significant spatial delocalization that inherently degrades the true spatial resolution well beyond the physical size of the electron probe. Furthermore, extracting the genuine inelastic onset requires overcoming formidable spectral artifacts, including specimen-induced Cherenkov radiation and guided optical modes.

Beyond improvements in spatial and energy resolution, a new challenge has emerged in the field of local bandgap characterization. Historically, advances in STEM-VEELS were largely driven by the pursuit of higher instrumental performance, including improved monochromation, aberration correction, and detector sensitivity. However, as modern instruments routinely achieve sub-100 meV energy resolution and nanometer-scale spatial resolution, the central question is no longer whether local bandgaps can be measured, but whether the measured values are reliable, reproducible, and comparable across different laboratories and experimental platforms. Consequently, increasing attention is being directed toward measurement uncertainty, data reliability, and methodological harmonization. Establishing robust protocols for specimen preparation, spectral acquisition, background treatment, ZLP subtraction, bandgap extraction, and uncertainty evaluation has become essential for transforming local bandgap measurements from a qualitative characterization tool into a quantitative metrological framework.

This review critically examines the current state, fundamental challenges, and future directions of local bandgap metrology using monochromated STEM-VEELS. Particular emphasis is placed on the physical origins of low-loss electronic excitations, the practical limitations associated with quantitative bandgap extraction, and emerging strategies to improve measurement reliability and reproducibility. We discuss the fundamental principles of interband transitions and localized optical excitations, experimental approaches for minimizing spatial delocalization, and robust methodologies for ZLP subtraction, onset determination, and suppression of Cherenkov-related artifacts. Representative applications in quantum dots, complex oxide interfaces, and two-dimensional materials are presented to illustrate the unique capabilities of STEM-VEELS for nanoscale electronic structure characterization. Finally, developments in artificial intelligence, uncertainty quantification, and data-driven analysis are reviewed, highlighting the ongoing transition from instrument-limited measurements toward standardized, reliable, and interoperable local bandgap metrology. Ultimately, monochromated STEM-VEELS is evolving from a high-resolution characterization technique into a standardized platform for quantitative nanoscale electronic metrology.

## Fundamentals of STEM-VEELS for local bandgap analysis

Over the past decade, monochromated STEM-VEELS has evolved from a qualitative low-loss spectroscopy technique into a quantitative nanoscale metrology platform. Although dielectric theory and Kramers–Kronig analysis remain central to understanding low-loss electron excitations, practical applications increasingly focus on the reliable extraction of local electronic and optical properties from spatially confined regions. The ability of monochromated STEM-VEELS to determine local bandgap energies originates from the interaction of fast electrons with the electronic structure of a material. Unlike conventional optical spectroscopies, which probe electronic transitions through photon absorption, STEM-VEELS measures the energy lost by incident electrons as they excite valence electrons, collective oscillations, and other low-energy electronic states within a specimen. Because these excitations are directly linked to the dielectric response and electronic band structure of the material, the low-loss EELS spectrum contains rich information regarding bandgaps, excitons, plasmons, optical transitions, and dielectric functions (Egerton 2011; Stöger-Pollach 2008).

Although the concept of bandgap determination from low-loss EELS appears straightforward, quantitative extraction of local bandgap values is considerably more complex. The measured spectrum represents a convolution of intrinsic electronic transitions with instrumental broadening, multiple scattering, spatial delocalization, zero-loss peak (ZLP) tails, and other parasitic optical processes. Consequently, reliable bandgap determination requires a thorough understanding of the physical origin of low-loss excitations, the relationship between the dielectric response and the electronic density of states, and the practical methodologies for identifying the true onset of interband transitions. This section, therefore, introduces the fundamental physics underlying local bandgap analysis and establishes the theoretical framework required for subsequent discussions of experimental methodologies, spectral processing, and quantitative metrology.

### Physical origin of bandgap information in low-loss EELS

The physical basis of local bandgap measurements using monochromated STEM-VEELS lies in the intimate relationship between low-loss electron excitations and the dielectric response of a material. When an incident electron traverses a specimen, it may transfer part of its kinetic energy to the electronic system through inelastic scattering processes. For semiconductors and insulators, these low-energy excitations include interband transitions, excitonic excitations, and collective oscillations of valence electrons. Among these processes, interband transitions are of particular importance because they provide direct information regarding the energy separation between occupied valence-band states and unoccupied conduction-band states.

Within the dielectric formalism, the electronic response of a material is described by the complex dielectric function.

1 $$\:\begin{array}{cccc}&\:\epsilon\left(\omega\:\right)={\epsilon}_{1}\left(\omega\right)+i{\epsilon}_{2}\left(\omega\right)&\:&\end{array}$$

where ε_1_(ω) and ε_2_(ω) represent the real and imaginary components of the dielectric function, respectively. Physically, ε_1_(ω) governs the dispersive response of the material, whereas ε_2_(ω) describes energy absorption arising from electronic excitations. Because low-loss EELS is fundamentally coupled to the dielectric response of the specimen, information contained within ε₂(ω) can be directly related to the optical and electronic properties of the material (Egerton 2011; Cheynet et al. 2007).

It should be noted that STEM-VEELS does not directly measure the dielectric function itself. Instead, the experimentally observed low-loss spectrum is approximately proportional to the energy-loss function.

2 $$Im\left[-\frac{1}{\epsilon\left(\omega\right)}\right]$$

which describes the probability of energy transfer from the incident electron to the specimen. Because the measured spectrum is related to the loss function rather than directly to ε_1_(ω) or ε_2_(ω), reconstruction of the dielectric response generally requires Kramers–Kronig analysis (KKA) (Egerton 2011; Stöger-Pollach 2008). KKA is based on the principle of causality, which dictates that the real and imaginary components of a response function cannot vary independently. Consequently, knowledge of one component permits reconstruction of the other through a Hilbert-transform relationship. For example, the real part of the dielectric function can be obtained from ε_2_(ω) through.

3 $${\epsilon}_{1}\left(\omega\right)-1=\frac{2}{\pi}P{\int}_{0}^{\infty}\frac{{\omega}^{{\prime}{\epsilon}_{2}}\left({\omega}^{{\prime}}\right)}{{{\omega}^{{\prime}}}^{2}-{\omega}^{2}}\;d{\omega}^{{\prime}}$$

where P denotes the Cauchy principal value. An analogous relation also exists for determining ε₂(ω) from ε₁(ω). Therefore, knowledge of one component over a sufficiently broad energy range permits reconstruction of the other component.

Application of KKA to low-loss EELS data allows determination of a wide range of optical properties, including the complex dielectric function, refractive index, extinction coefficient, optical conductivity, and plasmon characteristics. Historically, KKA has been one of the most powerful tools in VEELS analysis because it transforms experimentally measured spectra into physically meaningful optical constants that can be directly compared with optical spectroscopy measurements and theoretical calculations.

Nevertheless, several practical limitations must be considered when applying KKA. The procedure assumes accurate removal of plural scattering, proper normalization of the low-loss spectrum, and reliable subtraction of the zero-loss peak. Furthermore, finite energy ranges, detector noise, and thickness-dependent multiple scattering may introduce systematic errors into the reconstructed dielectric function. For nanoscale bandgap measurements, these limitations often make direct onset analysis more robust than full reconstruction of the dielectric function. Consequently, while KKA remains indispensable for understanding the physical origin of low-loss excitations and for extracting comprehensive optical properties, many modern monochromated STEM-VEELS studies rely primarily on onset-determination methods for quantitative local bandgap measurements.

For excitation energies below the bandgap threshold, electronic transitions between the valence band and conduction band are forbidden because no energetically accessible final states exist. Consequently, the contribution of interband transitions to ε_2_(ω) remains negligible. Once the excitation energy exceeds the bandgap energy, however, transitions become energetically allowed, and the absorption probability increases rapidly. This increase manifests as a characteristic onset in both the dielectric response and the measured low-loss EELS spectrum. Therefore, the bandgap information contained within a VEELS spectrum is fundamentally associated with the emergence of allowed interband transitions.

The physical origin of local bandgap measurements lies in the onset of interband transitions. As the energy loss approaches the bandgap threshold, the number of allowed electronic transitions from the valence band to the conduction band increases rapidly. This behavior can be described by the joint density of states (JDOS), which represents the total number of available electronic transitions at a given excitation energy. The most rigorous definition of the JDOS is obtained by explicitly summing all possible electronic transitions that satisfy energy conservation:

4 $$JDOS\left(E\right)=\varSigma(v,c,k)\delta\left[{E}_{C}\right(k)-{E}_{V}(k)-E]$$

where $\:{E}_{v}\left(k\right)$and $\:{E}_{c}\left(k\right)$are the energies of the valence-band and conduction-band states at crystal momentum * k*, respectively, * E* is the excitation energy, and $\:\delta\:$ denotes the Dirac delta function. This expression counts all electronic transitions whose energy difference matches the excitation energy * E*. The density of states (DOS) of the valence and conduction bands can be written as:

5 $${\rho}_{v}\left(E\right)=\varSigma(v,k)\delta[E-{E}_{v}(k\left)\right]$$

6 $${\rho}_{c}\left(E\right)=\varSigma(c,k)\delta[E-{E}_{c}(k\left)\right]$$

Substituting these definitions into Eq. (4) and transforming the summation over electronic states into an energy-space representation yields.

7 $$JDOS\left(E\right)=\int\;\rho_{v}\left(E^{\prime}\right)\rho_{c}\left(E^{\prime}+E\right)dE^{\prime}$$

where $\:{\rho\:}_{v}\left({E}^{{\prime\:}}\right)$and $\:{\rho\:}_{c}({E}^{{\prime\:}}+E)$represent the densities of occupied and unoccupied electronic states, respectively. Equation (7) shows that the JDOS can be interpreted as the overlap between occupied valence-band states and available conduction-band states separated by an energy * E*.

As the excitation energy approaches the bandgap threshold, an increasing number of transitions become energetically accessible, resulting in a rapid increase in the JDOS. This increase is reflected in the imaginary part of the dielectric function, which is approximately proportional to the product of the JDOS and the optical transition matrix element:

8 $${\epsilon}_{2}\left(E\right)\propto{M\left(E\right)}^{2}\cdot{JDOS}\left(E\right)$$

where $\:M\left(E\right)$ is the optical transition matrix element. Consequently, the onset of interband transitions first appears as an increase in the JDOS, followed by a rise in $\:{\epsilon\:}_{2}\left(E\right)$, which ultimately manifests as the low-loss spectral onset observed in monochromated STEM-VEELS spectra. (Zhang et al. 2008).

The shape of this onset depends strongly on the nature of the electronic transition. In direct-bandgap semiconductors, electrons can transition from the valence-band maximum to the conduction-band minimum without a change in crystal momentum. As a result, the transition probability increases abruptly above the bandgap energy, producing a relatively sharp onset in the low-loss spectrum. Typical examples include GaAs, InP, and many III–V compound semiconductors. In contrast, indirect-bandgap materials require simultaneous phonon participation to satisfy momentum conservation. Because both energy and momentum conservation must be fulfilled, the transition probability near the band edge is reduced, resulting in a broader and more gradual onset. Silicon is the most representative example of this behavior. Consequently, onset determination is generally more straightforward in direct-bandgap materials than in indirect-bandgap systems.

From an experimental perspective, the local bandgap is not determined directly from ε_2_(ω) itself but rather from the onset behavior associated with its rapid increase. One approach to identifying this transition region is derivative analysis, in which the energy-dependent rate of change of $\:{\epsilon\:}_{2}\left(\omega\:\right)$ is evaluated:

9 $$\frac{d\epsilon_{2}\left(\omega\right)}{d\omega}$$

The derivative spectrum highlights the energy range where the absorption probability changes most rapidly and can therefore assist in identifying the onset of interband transitions. However, derivative-based methods are highly sensitive to noise and spectral broadening, particularly in low signal-to-noise datasets. Consequently, derivative analysis is most commonly used as a complementary tool rather than a standalone method for determining bandgap. The onset of interband transitions initially appears as an increase in the JDOS, followed by a rapid rise in $\:{\epsilon\:}_{2}\left(\omega\:\right)$. This increase ultimately manifests as the low-loss spectral onset from which the local bandgap is determined. Understanding this chain of relationships is essential because all practical bandgap extraction methods discussed in the following section are fundamentally based on accurately and reproducibly identifying this onset.

Therefore, local bandgap analysis using monochromated STEM-VEELS should not be viewed simply as identifying the first detectable signal above the background. Rather, it represents a quantitative determination of the threshold energy at which interband electronic transitions become energetically allowed. This physical framework provides the foundation for the onset-determination methodologies discussed in Sect. "Practical Challenges in Local Bandgap Extraction".

### Practical challenges in local bandgap extraction

Although the physical origin of bandgap information in low-loss EELS is well understood, accurate extraction of local bandgap values remains a significant experimental challenge. In an ideal spectrum, the onset of interband transitions would appear as a clearly defined threshold corresponding directly to the bandgap energy. In practice, however, the measured signal represents a convolution of intrinsic electronic excitations with instrumental broadening, residual zero-loss peak (ZLP) tails, multiple scattering events, detector noise, and specimen-dependent artifacts. Consequently, the apparent onset observed in a raw VEELS spectrum often differs substantially from the true interband transition threshold.

Among these factors, the zero-loss peak is generally regarded as the most critical obstacle to reliable bandgap determination. The ZLP originates from elastically scattered electrons that have undergone no measurable energy loss and therefore appear as an intense peak centered at 0 eV. Even in state-of-the-art monochromated STEM-EELS systems, the finite energy spread of the electron source and spectrometer response produces a non-negligible tail extending into the low-energy region. Because semiconductor bandgaps frequently lie within the range of 0.5–5 eV, the weak interband transition signal of interest is often partially obscured by the residual ZLP tail. The severity of this issue becomes increasingly pronounced for narrow-bandgap semiconductors and materials exhibiting weak absorption edges. In such cases, the residual ZLP intensity may exceed the intrinsic onset signal by several orders of magnitude. Consequently, accurate determination of the bandgap requires rigorous removal or modeling of the ZLP prior to onset analysis. Failure to adequately account for the ZLP may result in systematic overestimation or underestimation of the bandgap energy.

Several approaches have been proposed for ZLP removal. One of the most widely adopted strategies involves acquiring a reference ZLP using either vacuum spectra or dual-EELS measurements, followed by subtraction from the specimen spectrum. This approach has been successfully employed in numerous monochromated STEM-VEELS studies because it directly incorporates the instrumental response function measured under identical experimental conditions. Alternatively, analytical functions can be fitted to the ZLP profile and subsequently removed from the measured spectrum. Depending on the spectral characteristics and instrument configuration, Gaussian, Lorentzian, exponential-tail, and hybrid fitting models have all been reported in the literature. Table 4 summarizes representative ZLP-removal approaches commonly employed in local bandgap measurements.

**Table 4:** Representative Zero-Loss Peak (ZLP) removal methods for STEM-VEELS bandgap analysis

Method	Principle	Advantages	Limitations
Reference ZLP subtraction	Subtract vacuum or dual-EELS reference spectrum	Physically realistic instrument response	Requires high-quality reference acquisition
Reflected-tail method	Assumes symmetric ZLP and reflects one side of the peak	Simple and computationally efficient	Sensitive to peak asymmetry
Gaussian fitting	Models ZLP using a Gaussian function	Suitable for symmetric monochromated spectra	Poor description of long tails
Lorentzian fitting	Represents extended spectral tails	Better tail description	May overestimate peak width
Gaussian + Lorentzian fitting	Combines narrow core and extended tail components	Widely used for semiconductor bandgap studies	Fitting parameter dependence
Exponential or power-law tail fitting	Models long-tail background contribution	Effective for low-intensity onsets	Model-dependent
Machine-learning-based estimation	Data-driven reconstruction of ZLP profile	Automated and potentially reproducible	Requires training and validation datasets

Even after successful ZLP removal, several additional factors can influence the onset determination. Multiple scattering may distort the low-loss spectrum, particularly in thicker specimens, while detector noise and limited counting statistics can obscure weak interband transitions. Furthermore, optical artifacts such as Cherenkov radiation and guided optical modes may generate additional spectral intensity within the bandgap region, leading to apparent reductions in the measured bandgap energy. These effects are discussed in detail in subsequent sections because they represent some of the most significant sources of systematic uncertainty in monochromated STEM-VEELS measurements.

Another important consideration is that no universally accepted criterion exists for defining the exact onset position. Depending on the analysis methodology, the same spectrum may yield slightly different bandgap values. Consequently, the quality of the ZLP subtraction procedure directly influences all subsequent stages of bandgap extraction, including baseline correction, onset fitting, derivative analysis, and machine-learning-assisted interpretation.

For these reasons, modern local bandgap metrology increasingly treats ZLP removal not merely as a preprocessing step but as an integral part of the measurement itself. The reliability and reproducibility of the final bandgap value are often determined as much by the quality of the ZLP treatment as by the intrinsic energy resolution of the instrument. Once the influence of the ZLP has been minimized, the remaining challenge is identifying the true onset energy of interband transitions. Various methodologies for determining onset developed for this purpose are discussed in the following section.

### Bandgap onset determination methods

Following the appropriate removal of the zero-loss peak (ZLP) and background contributions, the next critical step in local bandgap analysis is determining the interband-transition onset. Although the physical origin of the onset is well understood, its practical identification remains one of the most challenging aspects of STEM-VEELS analysis. In an ideal spectrum, the onset of interband transitions would appear as a well-defined threshold corresponding directly to the bandgap energy. In practice, however, the measured signal is broadened by finite instrumental energy resolution, residual ZLP tails, multiple scattering, detector noise, and specimen-dependent effects. Consequently, different onset-determination methodologies may produce different bandgap values even when applied to the same spectrum.

Among the various approaches reported in the literature, linear extrapolation remains the most widely adopted method because of its simplicity and intuitive physical interpretation (Batson et al. 1986; Egerton 2011). In this approach, a linear function is fitted to the steepest portion of the onset edge and extrapolated toward the baseline. The intersection between the fitted line and the baseline is then defined as the bandgap energy. Because the method directly utilizes the spectral region associated with interband transitions, it provides a straightforward estimate of the onset energy and can be readily implemented in most datasets. However, the extracted value is often sensitive to the selected fitting range, smoothing procedure, and signal-to-noise ratio. Consequently, operator-dependent variability remains one of the primary limitations of this method.

Derivative-based approaches provide an alternative strategy for onset identification. Rather than relying solely on the visual appearance of the spectral edge, the first derivative of the spectrum is evaluated to identify the energy range where the spectral intensity changes most rapidly (Keller et al. 2014; Korneychuk et al. 2018a, b; Stöger-Pollach 2008). Because the onset of interband transitions is associated with a rapid change in intensity, derivative analysis can enhance weak spectral features that may be difficult to distinguish in the original spectrum. In some cases, inflection points or local extrema in the derivative spectrum provide useful indicators of the onset energy. Derivative-based methods are particularly attractive because they reduce the subjective selection of fitting windows and may facilitate semi-automated analysis. However, numerical differentiation amplifies high-frequency noise and therefore generally requires smoothing or filtering prior to analysis. As a result, derivative methods are most commonly used as complementary tools rather than standalone approaches for quantitative bandgap determination.

A more physically motivated approach is based on Tauc-type analysis, which was originally developed for optical absorption spectroscopy and later adapted for EELS-based bandgap measurements (Tauc et al. 1966; Davis and Mott 1970). Because the onset observed in low-loss EELS originates from electronic transitions across the bandgap, the spectral intensity near the onset may be analyzed using relationships analogous to those employed in optical absorption studies. After appropriate background and ZLP subtraction, the inelastic intensity near the band edge can be approximated by.

10 $$I\left(E\right)=C\left(E-E_{g}\right)^n$$

where *I(E)* is the measured inelastic intensity at energy loss *E*, *C* is a proportionality constant, $\:{E}_{g}$ is the bandgap energy, and * n* describes the characteristic onset behavior associated with the electronic transition type.

**Fig. 2:**
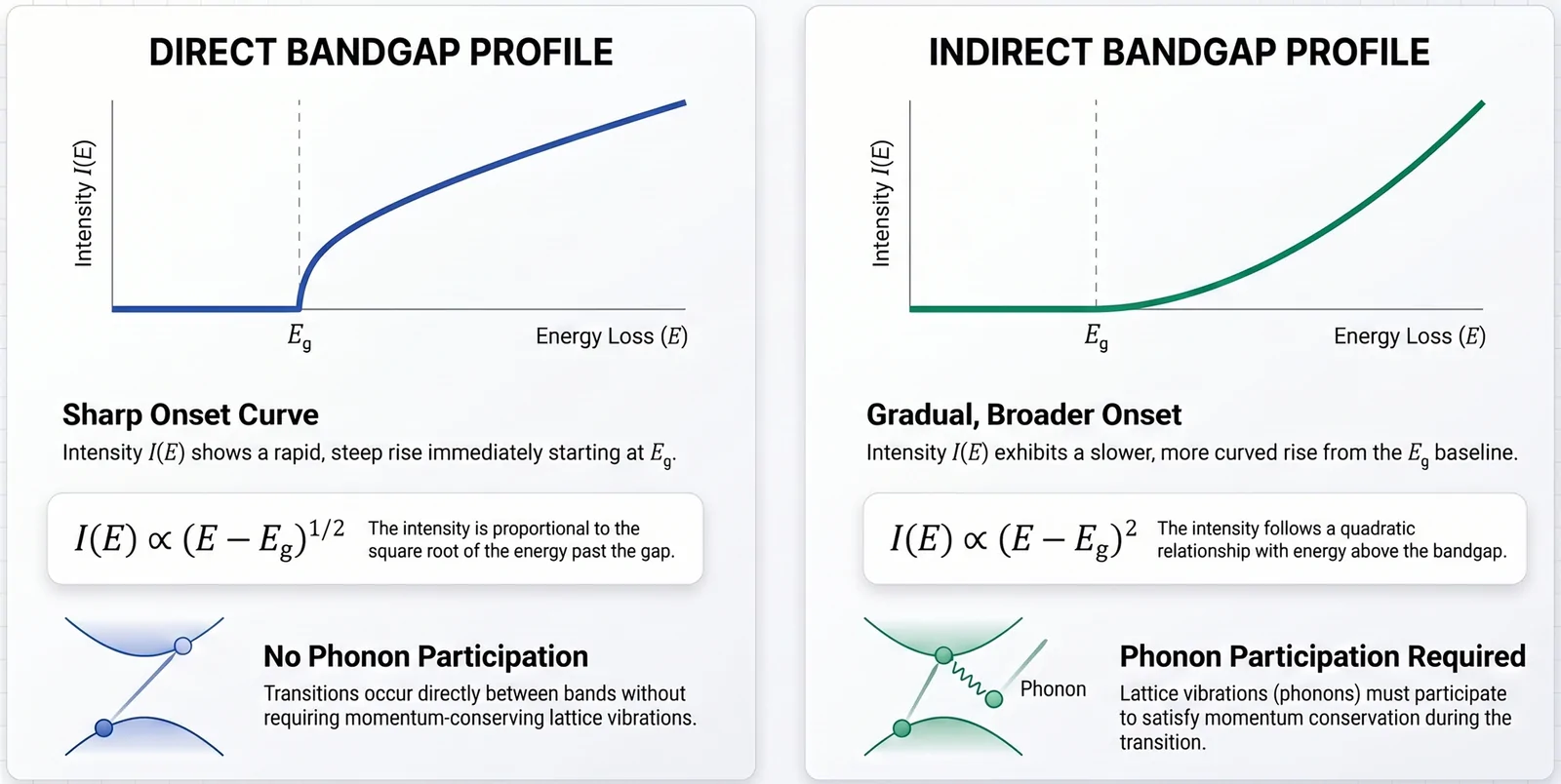
Comparison of idealized onset profiles for direct and indirect electronic transitions. Direct transitions exhibit an onset behavior approximately proportional to $\:{\left(E-{E}_{g}\right)}^{1/2}$, whereas indirect transitions are commonly described by a $\:{\left(E-{E}_{g}\right)}^{2}$dependence owing to phonon-assisted momentum conservation. The schematic illustrates the different onset evolutions used in bandgap fitting and onset-determination analyses in STEM-VEELS. Deviations from these idealized profiles may occur in real materials because of confinement effects, excitonic interactions, disorder, and experimental broadening. The direct and indirect transition profiles shown here represent the optical-limit picture, in which the photon momentum is assumed to be negligible (q ≈ 0). In STEM-VEELS, however, finite momentum transfer (q ≠ 0) from the incident electron can contribute to transitions between electronic states separated in momentum space, which is particularly relevant for indirect-gap materials. The experimentally observed bandgap onset may therefore depend on the momentum-transfer distribution and experimental geometry, as discussed in the text

For direct allowed transitions, the onset intensity generally increases rapidly above the band edge. In an ideal three-dimensional semiconductor, the joint density of states predicts an onset behavior approximately proportional to.

11 $$Direct\;bandgap:I\left(E\right)\propto\left(E-E_{g}\right)^{1/2}$$

which corresponds to $\:n\approx\:1/2$. Physically, this behavior arises because electronic transitions can occur without phonon participation, resulting in a rapid increase in the number of available final states above the bandgap energy as can be seen in Fig. 2. Consequently, direct-bandgap materials typically exhibit a relatively sharp onset but convergent saturation in low-loss EELS spectra. This is because the JDOS in the 3D materials has *E*^0.5^ trend unless the system has lower dimensions, such as a quantum dot (0D), nanowire (1D), or monolayer/nanosheet (2D).

For indirect transitions, momentum conservation requires simultaneous phonon participation, leading to a gradual onset. In the simplest approximation, the onset intensity may be described by.

12 $$Indirect\;bandgap:I\left(E\right)\propto\left(E-E_{g}\right)^2$$

corresponding to $\:n\approx\:2$. Compared with direct transitions, indirect transitions exhibit a gradual increase immediately upon onset and a parabolic divergence, often making precise bandgap determination more challenging. Because the signal near the onset point has very low electron counts, some uncertainty is always brought up.

It should be emphasized, however, that the exponents given in Eqs. (4) and (5) represent idealized cases derived from simple band-structure models. In practical STEM-VEELS measurements, the observed onset shape may deviate significantly from these values owing to dimensional confinement, excitonic effects, disorder, phonon broadening, instrumental resolution, and residual background contributions. Consequently, many fitting procedures treat * n* as an adjustable parameter rather than a strictly fixed quantity, particularly when analyzing low-dimensional materials, complex heterostructures, or strongly correlated systems. One distinctive feature to note is that the direct bandgap exhibits convergent saturation, whereas the indirect bandgap shows parabolic divergence near the bandgap (Fig. 2).

The direct and indirect transition profiles illustrated in Fig. 2 represent the optical-limit picture. In STEM-VEELS, however, finite momentum transfer from the incident electron can also contribute to electronic transitions. Consequently, the experimentally observed bandgap onset may depend not only on the intrinsic electronic structure but also on the momentum-transfer distribution and experimental geometry. In particular, a larger collection semi-angle (β) includes electrons scattered to larger angles and therefore samples a broader range of momentum transfers, while the convergence semi-angle (α) determines the angular distribution of the incident probe and further broadens the momentum-transfer distribution. Thus, even for the same material, variations in α, β, and acceleration voltage can alter the range and weighting of momentum transfers sampled in an EELS measurement. This is particularly relevant for indirect-gap materials, where finite-q transitions can contribute to the measured low-loss spectrum, such that the EELS onset does not necessarily follow the simple q ≈ 0 selection-rule picture commonly used for optical absorption. The specimen orientation can also be important because it determines the relationship between the momentum-transfer direction and the relevant directions in the Brillouin zone. Therefore, the optical-limit description of direct and indirect transitions should be regarded as an approximation when interpreting STEM-VEELS bandgap onsets. These considerations become particularly important for momentum-dependent electronic excitations, as discussed in a later section. From the perspective of Fermi’s golden rule, the transition probability is determined by both the available initial and final electronic states and the momentum-dependent transition matrix element. Unlike optical absorption, where the photon momentum is generally negligible (q ≈ 0), EELS can involve finite momentum transfer (q ≠ 0), allowing electronic transitions over a broader region of momentum space. This distinction is particularly relevant for indirect-gap materials, where the valence-band maximum and conduction-band minimum occur at different points in the Brillouin zone.

This limitation becomes particularly important when analyzing modern nanoscale materials. For example, quantum dots, two-dimensional semiconductors, moiré heterostructures, and complex oxide interfaces often exhibit electronic structures that differ substantially from those assumed in simple three-dimensional band models. In such systems, the exponent (n) may deviate significantly from the idealized values predicted by conventional JDOS theory. Consequently, many practical fitting procedures treat (n) as an adjustable parameter rather than a strictly fixed quantity. This approach often provides a more realistic representation of experimental spectra while preserving the physical interpretation of the onset behavior.

Beyond analytical fitting approaches, model-based methods have also been developed to improve robustness and reduce operator bias. Rather than fitting only the onset region, these methods attempt to reproduce the measured spectrum using physically constrained models of the dielectric response or the joint density of states (Rafferty and Brown 1998). Because the fitting procedure is directly linked to the underlying electronic structure, model-based approaches can provide improved physical interpretability and reduced sensitivity to subjective fitting choices. However, their accuracy depends strongly on the validity of the assumed model and often requires significantly greater computational effort.

Machine learning have introduced a new generation of automated onset-determination techniques. Granerød et al. demonstrated automated bandgap mapping workflows capable of processing large STEM-EELS datasets with minimal user intervention. More recently, Roest et al. developed an open-source Python package dubbed EELSfitter, a machine-learning-based framework capable of model-free ZLP estimation and automated bandgap extraction from low-loss spectra (Roest et al. 2021). Similarly, the Verbeeck group and other researchers have demonstrated automated fitting workflows designed to improve reproducibility while minimizing subjective user intervention. Deep-learning approaches based on convolutional neural networks and variational autoencoders have also been explored for spectrum-image datasets, enabling simultaneous analysis of both spatial and spectral information. These developments are particularly attractive for modern monochromated STEM-VEELS experiments, where large multidimensional datasets can render manual fitting procedures impractical.

Table 5 summarizes the most representative onset-determination methodologies currently employed in STEM-VEELS bandgap analysis. Although each approach offers distinct advantages, no single method is universally optimal. The choice of analysis strategy depends strongly on spectral quality, material system, energy resolution, and the specific scientific question being addressed.

**Table 5:** Comparison of representative bandgap onset determination methods

Method	Principle	Advantages	Limitations
Linear extrapolation	Fit onset edge and extrapolate to baseline	Simple, intuitive, widely used	User dependent
First derivative	Detect maximum rate of spectral change	Objective onset identification	Highly sensitive to noise
Slope-change method	Identify inflection region	No explicit fitting required	Threshold selection may be subjective
3σ criterion	Statistical threshold above background noise	Quantitative and reproducible	Requires accurate noise estimation
Tauc-type fitting	Transition-model-based analysis	Physically meaningful	Requires assumptions regarding transition type
JDOS/model fitting	Electronic-structure-based fitting	Reduced operator bias	Computationally intensive
Machine learning	Automated onset extraction	High reproducibility and throughput	Dependent on training data quality

Most importantly, different onset-determination methods applied to the same spectrum may yield different bandgap values. Therefore, the reported bandgap should not be regarded solely as a material property but also as a quantity influenced by the chosen analysis methodology. From a metrological perspective, rigorous uncertainty evaluation, transparent reporting of fitting procedures, and harmonized analysis protocols are essential for ensuring reproducibility and comparability of local bandgap measurements across different laboratories. As monochromated STEM-VEELS continues to evolve from a characterization technique into a quantitative nanoscale metrology platform, standardization of onset-determination procedures will become increasingly important for establishing reliable and traceable local bandgap measurements.

### Spatial delocalization and fundamental limits of local bandgap measurements

Although modern monochromated STEM instruments can routinely form sub-angstrom electron probes, it is important to distinguish the STEM probe size from the effective spatial resolution of a local bandgap measurement. The probe size represents the spatial extent of the incident electron beam, whereas the effective spatial resolution of STEM-VEELS is additionally limited by the delocalized nature of inelastic scattering processes. This distinction is particularly important for low-loss excitations near the bandgap because the interaction responsible for interband transitions can extend significantly beyond the physical dimensions of the incident electron probe (Egerton 2011; Stöger-Pollach 2008). Consequently, the effective spatial resolution of local bandgap measurements may be substantially poorer than the nominal imaging resolution of the microscope and is determined by the combined effects of probe size, inelastic-scattering delocalization, collection geometry, specimen thickness, and the energy-loss range used for bandgap determination. From a quantitative metrology perspective, the probe diameter should therefore not be directly interpreted as the spatial resolution of the bandgap measurement itself.

The origin of this limitation can be understood in terms of the long-range Coulomb interaction between the incident fast electron and the electronic states within the specimen. Unlike core-loss excitations, which are generally localized around individual atomic sites, low-loss excitations are governed predominantly by dipole-allowed transitions and therefore exhibit significantly larger interaction volumes (Egerton 2011). As first discussed by Isaacson and Johnson and later formalized by Egerton, the characteristic delocalization length increases rapidly as the energy loss decreases (Isaacson and Johnson 1975; Egerton 2011). Consequently, the low-energy regime relevant to semiconductor bandgap measurements represents the most challenging region for achieving true nanoscale spatial resolution.

A useful approximation for the characteristic delocalization length is given by

13 $$L\propto\frac{E_{0}}{\triangle{E}}$$

where * L* is the delocalization length, * E₀* is the incident beam energy, and $\:\varDelta\:E$ is the energy loss associated with the excitation. This relationship indicates that lower-energy excitations are inherently more delocalized than higher-energy excitations. For typical semiconductor bandgaps in the range of 1–3 eV, the interaction volume may extend over several to tens of nanometers despite the use of an atomic-scale electron probe (Egerton 2011; Stöger-Pollach 2008).

The degree of localization can also be described in reciprocal space through the momentum transfer, 𝑞, associated with the inelastic scattering event. Under the small-angle approximation,

14 $$q\approx\triangle{E}/\left(v\hslash\right)$$

where * v* is the electron velocity and $\:\hslash\:$ is the reduced Planck constant (Egerton 2011). Because small energy losses correspond to small momentum transfers, low-loss EELS preferentially probes long-range excitations. This reciprocal-space interpretation provides a useful framework for understanding why bandgap measurements are particularly sensitive to experimental collection conditions.

In practical STEM-VEELS experiments, both the convergence and collection semi-angles influence the range of momentum transfers that contribute to the measured spectrum. Increasing the convergence semi-angle improves probe formation and spatial resolution, while simultaneously increasing the range of incident wave vectors that contribute to the measured momentum-transfer distribution. Likewise, larger collection angles incorporate scattering events associated with wider momentum-transfer distributions. Consequently, optimization of electron-optical parameters often involves balancing spatial resolution, signal intensity, and localization (Egerton 2011; García de Abajo 2010; Hage et al. 2013; Korneychuk et al. 2018a, b).

These considerations have motivated the development of momentum-filtered and off-axis acquisition strategies designed to suppress strongly delocalized excitations and preferentially collect localized interband-transition signals. Such approaches have proven particularly valuable for nanoscale bandgap measurements in semiconductor heterostructures, quantum wells, and low-dimensional materials (Hage et al. 2013; Korneychuk et al. 2018a, b; Lagos et al. 2017). In particular, off-axis STEM-EELS geometries can selectively suppress long-range retardation effects and enhance sensitivity to localized electronic excitations, improving the reliability of local bandgap measurements.

Therefore, local bandgap measurements should not be interpreted solely on the basis of probe size or pixel spacing. Instead, the effective spatial resolution must be considered in the context of the underlying excitation physics and the associated momentum-transfer distribution. Understanding these fundamental limitations is essential for the rigorous interpretation of STEM-VEELS bandgap measurements and provides the foundation for the discussion of measurement uncertainties and spectral artifacts presented in the following section.

### Sources of uncertainty and spectral artifacts in local bandgap measurements

Although significant advances in monochromation, detector technology, and spectral analysis have greatly improved the reliability of STEM-VEELS bandgap measurements, the quantitative determination of local bandgap energies remains subject to numerous sources of uncertainty. Unlike conventional optical spectroscopy, where measurements typically represent spatially averaged responses over macroscopic volumes, STEM-VEELS seeks to determine bandgap energies from highly localized regions containing limited signal intensity. Consequently, the extracted bandgap value may be influenced not only by the intrinsic electronic structure of the specimen but also by experimental conditions, spectral artifacts, and data-processing procedures. Understanding and quantifying these sources of uncertainty is therefore essential for establishing STEM-VEELS as a quantitative metrology tool rather than a purely qualitative characterization technique.

The uncertainty associated with local bandgap measurements originates from a combination of physical artifacts, specimen-related effects, instrumental limitations, and data-analysis procedures. Many of these factors directly influence the low-loss spectral region and can modify the apparent onset position used to determine the bandgap. As a result, experiments performed on nominally identical materials may yield different bandgap values if these sources of uncertainty are not adequately controlled.

One of the most important sources of systematic error in bandgap measurements originates from parasitic low-loss excitations that overlap with the interband-transition region. Among these, Cherenkov radiation is particularly significant in high-refractive-index materials. First reported by Cherenkov in 1934, this relativistic electromagnetic phenomenon occurs when the velocity of an incident electron exceeds the phase velocity of light in a dielectric medium (Cherenkov 1934; Vavilov 1934). Under this condition, coherent electromagnetic radiation is emitted, producing additional intensity in the low-loss region of the EELS spectrum. Unlike interband transitions, which arise from intrinsic electronic excitations across the bandgap, Cherenkov radiation arises from long-range electromagnetic interactions and therefore does not directly reflect the material’s local electronic structure (Egerton 2011; Stöger-Pollach 2008). In addition to Cherenkov radiation, guided optical modes, retardation effects, surface polaritonic excitations, and other nonlocal electromagnetic excitations may also contribute to the low-loss spectrum, introducing spectral features unrelated to intrinsic interband transitions (García de Abajo 2010; Lagos et al. 2017; Vatanparast et al. 2017). Consequently, these parasitic excitations can obscure the true bandgap onset and complicate quantitative interpretation of monochromated STEM-VEELS spectra.

The probability of Cherenkov radiation increases with increasing refractive index because the phase velocity of light decreases according to $\:{v}_{ph}=c/n$, where c is the light velocity in vacuum and n is the refractive index. Consequently, high-refractive-index (or low bandgap) semiconductors such as Si, GaAs, InP, and related III–V compounds are particularly susceptible to Cherenkov-related contributions, especially at conventional accelerating voltages of 200–300 kV (Stöger-Pollach 2008; Vatanparast et al. 2017). When the velocity of the incident electron exceeds the phase velocity of light within the specimen, electromagnetic radiation may contribute additional intensity in the low-loss region of the spectrum. Because this signal can appear close to the bandgap onset, it may artificially distort the apparent transition edge and lead to an erroneous bandgap determination. Reducing the accelerating voltage, employing thinner specimens, or utilizing off-axis acquisition geometries can partially suppress Cherenkov-related contributions. Table 6 illustrates the general empirical trend that materials with smaller bandgaps tend to exhibit larger refractive indices. This behavior is often described by the Moss relation, which predicts an inverse correlation between bandgap energy and refractive index. Consequently, narrow-bandgap semiconductors such as Ge, Si, GaAs, and InP are generally more susceptible to Cherenkov-related contributions in STEM-VEELS measurements because the phase velocity of light is reduced in high-index media.

**Table 6:** Representative materials illustrating the relationship between bandgap energy, refractive index, and Cherenkov threshold

Material	E_g_ (eV)	Refractive Index (*n*)	Approx. Cherenkov Threshold (kV)	Expected Cherenkov Contribution at 60 kV	Expected Cherenkov Contribution at 200 kV	Remarks
Ge	0.66	~ 4.0	~ 17	High	Very High	Narrow-gap semiconductor with a high refractive index
Si	1.12	~ 3.5	~ 22	High	Very High	High-index semiconductor; Cherenkov contribution remains significant at 60 kV
InP	1.35	~ 3.1	~ 29	Moderate–High	High	Typical III–V semiconductor
GaAs	1.42	~ 3.4	~ 23	High	Very High	High-index direct-bandgap semiconductor
Diamond	5.5	~ 2.4	~ 51	Low–Moderate	High	60 kV is close to the estimated threshold
HfO_2_	~ 5.8	~ 2.0	~ 79	Very Low	Moderate	60 kV is below the estimated threshold
TiO_2_	3.0–3.2	~ 2.5–2.9	~ 34–47	Moderate	High	Relatively high refractive index despite the wider bandgap
SrTiO_3_	~ 3.2	~ 2.4–2.6	~ 43–51	Low–Moderate	High	60 kV is relatively close to the estimated threshold
MgO	~ 7.8	~ 1.74	~ 113	Very Low	Low–Moderate	60 kV is below, whereas 200 kV is above the estimated threshold
SiO_2_	8–9	~ 1.45	~ 196	Very Low	Very Low–Low	60 kV is far below, whereas 200 kV is near the estimated threshold

Narrow-bandgap semiconductors typically exhibit larger refractive indices and are therefore more susceptible to Cherenkov-related contributions in STEM-VEELS measurements. Notable exceptions, including diamond, TiO_2_, and SrTiO_3_, are also included

The approximate Cherenkov threshold is estimated from the classical condition, where is the relativistic electron velocity, is the speed of light in vacuum, and is the representative refractive index. The expected Cherenkov contributions at 60 and 200 kV are qualitative estimates, as the actual contribution also depends on the energy-dependent refractive index, specimen thickness, and experimental geometry

With modern aberration-corrected STEM, spatial resolutions of approximately 0.08–0.12 nm at 60 kV and 0.05–0.08 nm at 200 kV can typically be achieved. Although lowering the acceleration voltage involves a trade-off with spatial resolution, aberration correction can substantially mitigate this limitation, allowing atomic-scale resolution to be retained even at lower acceleration voltages

However, the bandgap energy alone does not uniquely determine the refractive index. The dielectric response is also influenced by factors such as electronic polarizability, bonding characteristics, orbital hybridization, and oscillator strength. As a result, several notable exceptions exist. For example, TiO_2_ exhibits a relatively high refractive index despite its moderate bandgap of approximately 3.0–3.2 eV. This behavior originates from the strong polarizability associated with low-lying Ti 3d electronic states, which contribute significantly to the dielectric response. Similarly, SrTiO_3_ exhibits a relatively high refractive index due to the strong hybridization between O 2p and Ti 3d orbitals and the resulting enhancement of its dielectric polarizability. Diamond also represents an exception, maintaining a comparatively high refractive index despite its wide bandgap exceeding 5 eV. Therefore, Cherenkov susceptibility should be evaluated using the material’s actual refractive index rather than inferred solely from its bandgap energy.

Another important relativistic-loss mechanism arises from guided optical modes. Unlike interband transitions, which are localized electronic excitations, guided optical modes arise from interactions between fast electrons and electromagnetic waves supported by the specimen geometry. These modes can propagate along thin films, interfaces, multilayer structures, or nanostructures and may contribute additional intensity in the low-loss region. Because guided modes can extend over distances significantly larger than the electron probe size, they may introduce apparent spectral variations that do not directly correspond to changes in the local electronic structure (García de Abajo 2010; Vatanparast et al. 2017; Lagos et al. 2017).

When a fast electron beam traverses a specimen, it does not interact solely through direct excitation of valence electrons. Because the incident electron carries both electric and magnetic fields, its passage through the material can also induce collective electromagnetic responses of the specimen. Consequently, the measured low-loss EELS signal may contain not only interband transitions and plasmon excitations, but also additional electromagnetic excitations generated by the interaction between the moving electron and the dielectric medium.

The bandgap information of interest in STEM-VEELS originates from interband transitions, in which electrons are excited from occupied valence-band states to unoccupied conduction-band states. These electronic excitations directly reflect the intrinsic electronic structure of the material. However, the electric field associated with a fast electron can also perturb the dielectric medium itself, causing the material to generate electromagnetic waves or optical modes that are not directly related to band-to-band transitions.

A useful analogy is that of a fast-moving boat traveling across water. A slow boat generates only local disturbances, whereas a sufficiently fast boat creates long-range wake patterns that propagate away from its trajectory. Similarly, a relativistic electron can induce electromagnetic disturbances that extend beyond the immediate region of electronic excitation. These disturbances may subsequently appear in the measured low-loss spectrum even though they do not originate from intrinsic bandgap transitions.

Among these effects, Cherenkov radiation occurs when the electron velocity exceeds the phase velocity of light within the material,

15 $$v>\frac{c}{n}$$

where * v*is the electron velocity, * c*is the speed of light in vacuum, and * n*is the refractive index of the specimen. Under this condition, the polarized medium cannot respond rapidly enough to the passing electron, emitting coherent electromagnetic radiation. The emitted photons contribute additional intensity to the low-loss region and may overlap with the bandgap onset.

Guided optical modes arise through a related mechanism. Instead of emitting radiation into the surrounding medium, the electron excites electromagnetic modes that are confined within thin films, interfaces, multilayers, or nanostructures. Because these modes can propagate over distances much larger than the electron probe diameter, they may introduce apparent spectral variations that do not necessarily reflect local changes in electronic structure.

The term *retardation effect* arises because electromagnetic fields propagate at a finite velocity rather than acting instantaneously. In classical electrostatics, interactions are often treated as occurring immediately throughout space. However, for relativistic electrons used in STEM-EELS, the electromagnetic field generated by the moving electron requires a finite time to propagate through the specimen. This temporal delay, or *retardation*, modifies the electron–matter interaction and enables radiative electromagnetic excitations, such as Cherenkov radiation and guided optical modes. In this sense, Cherenkov radiation and guided optical modes can be regarded as manifestations of retardation-related electromagnetic interactions. Importantly, these electromagnetic excitations do not alter the intrinsic bandgap of the material. Instead, they introduce additional spectral intensity within the same low-energy region where interband transitions occur. As a result, the true bandgap onset may become obscured, leading to increased uncertainty in bandgap determination. This effect is particularly significant in high-refractive-index semiconductors and narrow-bandgap materials, where relativistic-loss contributions become more pronounced.

**Fig. 3:**
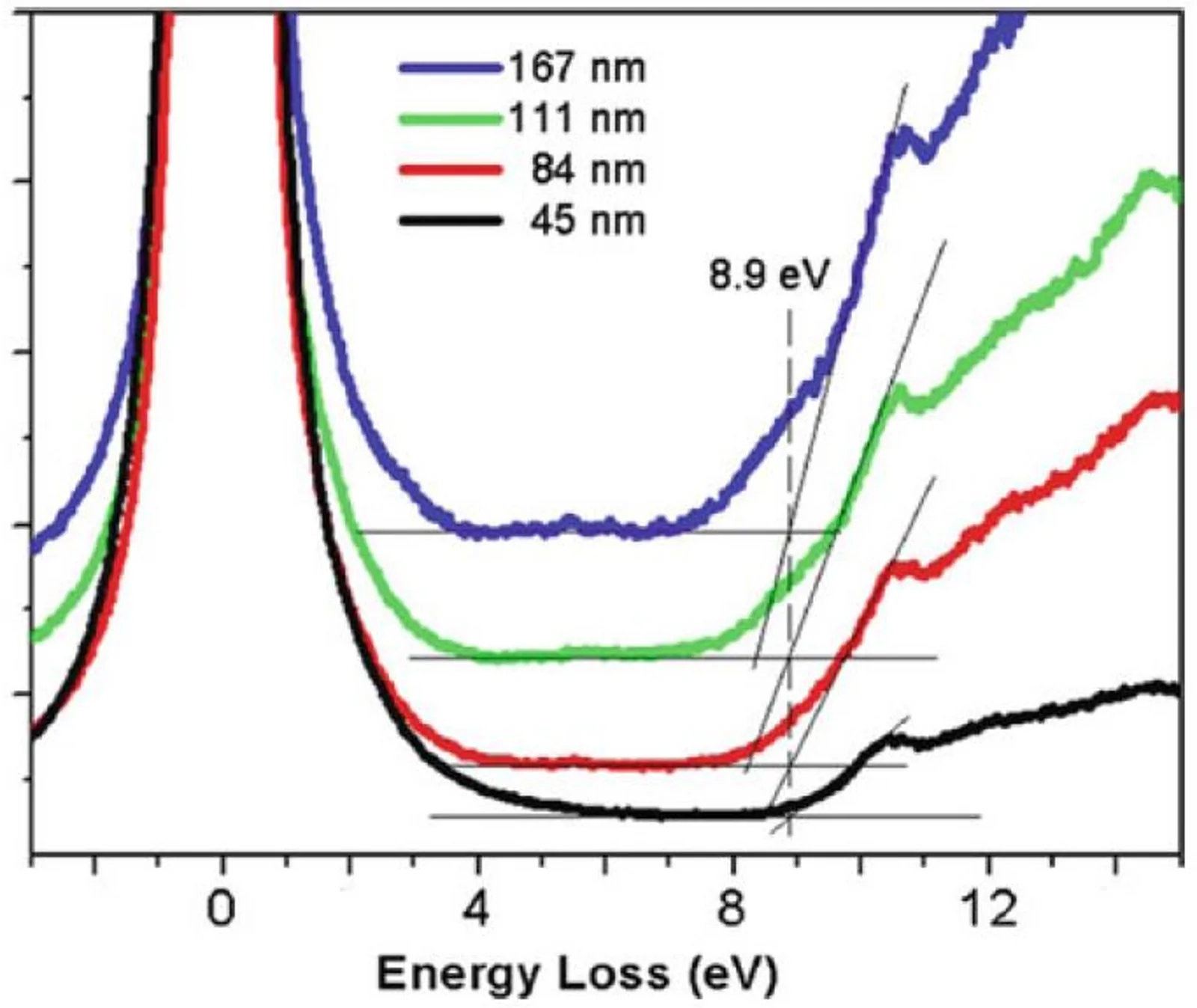
Bandgap measurements of amorphous SiO₂ films with different thicknesses using STEM-VEELS. The bandgap (~ 8.9 eV) was extracted by linear extrapolation of the rising edge of the inelastic signal and the background (= baseline). As the film thickness increases, the Cherenkov radiation component becomes more pronounced, distorting the true bandgap onset. Adapted from Ref. (Park et al. 2009)

Specimen-related factors also play a critical role. As discussed in Sect. "Spatial delocalization and fundamental limits of local bandgap measurements", spatial delocalization fundamentally limits the localization of low-energy excitations and may lead to spectral mixing across interfaces and nanoscale heterostructures (Egerton 2011; Stöger-Pollach 2008). In addition, specimen thickness influences the probability of plural scattering in Fig. 3, resulting in spectral broadening and distortion of the onset region. Although deconvolution procedures can partially recover the single-scattering distribution, uncertainties associated with thickness estimation and plural-scattering removal often remain (Egerton 2011). Experimental demonstrations of multiple-scattering correction in low-loss EELS have been reported by Batson and Silcox (1983). Figure 3 shows an example of the combined effects of multiple scattering and Cherenkov radiation in a thicker sample, emphasizing how thickness can disrupt bandgap measurements in practice.

Instrumental limitations provide another significant source of uncertainty. The finite energy resolution of the instrument, commonly characterized by the full width at half maximum (FWHM) of the zero-loss peak, limits the ability to resolve weak or closely spaced electronic transitions (Egerton 2011; Krivanek et al. 2014). Furthermore, residual energy drift, spectrometer instabilities, detector nonlinearity, and imperfect energy calibration may introduce systematic shifts in the extracted bandgap value, particularly when the expected bandgap variation is only a few tens of meV.

The final category of uncertainty originates from data processing and bandgap extraction procedures. As discussed in Sect.  "Bandgap onset determination methods", different onset-determination methodologies frequently produce different bandgap values when applied to the same spectrum. Linear extrapolation, derivative analysis, statistical thresholding, JDOS fitting, and machine-learning-based approaches all involve distinct assumptions regarding background treatment and onset behavior (Rafferty and Brown 1998; Keller et al. 2014; Roest et al. 2021; Brokkelkamp et al. 2022). In addition, subjective choices such as fitting-window selection, baseline definition, smoothing parameters, and noise filtering can introduce operator-dependent variability. Keller et al. (2014) demonstrated that acquisition reproducibility and fitting conditions can significantly affect both the precision and accuracy of local bandgap measurements, while Roest et al. highlighted the potential of machine-learning-based approaches to improve analysis consistency and reduce user bias.

For clarity, the major sources of uncertainty encountered in monochromated STEM-VEELS bandgap measurements are summarized in Table 7. The table categorizes the dominant sources of uncertainty by their physical origins, typical effects on bandgap determination, and representative mitigation strategies.

**Table 7:** Major sources of uncertainty in local bandgap measurements using monochromated STEM-VEELS

Source	Category	Physical origin	Typical effect on E_g_ determination	Mitigation strategy
Cherenkov radiation	Physical artifact	Electron velocity exceeds the phase velocity of light in the specimen	Artificial low-loss intensity near the onset region	Lower accelerating voltage, thinner specimens, off-axis EELS
Guided optical modes/retardation effects	Physical artifact	Long-range electromagnetic interactions	Additional low-loss features and apparent bandgap variations	Off-axis acquisition, momentum filtering
Spatial delocalization	Specimen effect	Long-range Coulomb interaction associated with low-energy excitations	Reduced effective spatial resolution and spectral mixing	Reduced collection angle, momentum-selective acquisition
Plural scattering	Specimen effect	Multiple inelastic scattering events in thicker specimens	Broadening of the onset region and inaccurate bandgap extraction	Thin specimens, Fourier-log deconvolution
Finite energy resolution	Instrumental limitation	Broadening of the zero-loss peak (ZLP)	Reduced ability to resolve weak or closely spaced transitions	Monochromation, spectrometer optimization
Energy drift/calibration error	Instrumental limitation	Instability of electron optics and spectrometer calibration	Systematic shift of extracted bandgap values	Frequent energy calibration using ZLP and reference materials
ZLP subtraction	Data analysis	Incomplete removal of elastic-scattering tails	Distortion of onset position and fitting bias	Optimized fitting models and reference ZLP acquisition
Onset fitting method	Data analysis	Different assumptions regarding edge shape and fitting range	Method-dependent variation of extracted Eg	Standardized fitting procedures
User-dependent parameters	Data analysis	Baseline definition, smoothing, and fitting-window selection	Reduced reproducibility between operators	Automated fitting and objective criteria
Machine-learning model uncertainty	Data analysis	Training-set dependence and model assumptions	Potential bias in automated bandgap extraction	Cross-validation and independent verification

As shown in Table 7, uncertainty in local bandgap measurements does not originate from a single factor but rather from the cumulative influence of specimen physics, instrumentation, and data-analysis procedures. Consequently, rigorous uncertainty evaluation requires consideration of the complete measurement workflow rather than any individual factor in isolation.

These considerations highlight an important distinction between precision and accuracy. A highly reproducible onset value obtained from a particular fitting procedure does not necessarily guarantee that the extracted bandgap accurately represents the true electronic bandgap of the material. Therefore, reporting only a single bandgap value without an accompanying uncertainty estimate may be insufficient for rigorous quantitative interpretation. As monochromated STEM-VEELS continues to evolve toward a quantitative nanoscale metrology platform, standardized acquisition protocols, reference materials, traceable calibration procedures, and harmonized analysis methodologies will become increasingly important (Stöger-Pollach 2008; Egerton 2011).

Ultimately, the future impact of STEM-VEELS will depend not only on achieving ever-higher spatial or energy resolution but also on establishing robust uncertainty quantification and reproducible measurement practices. Addressing these challenges will be critical to transforming local bandgap measurements from a qualitative analytical capability into a traceable, internationally comparable metrological technique.

## Experimental considerations for quantitative local bandgap measurements

While the physical principles governing local bandgap measurements using STEM-VEELS are reasonably well established, the practical realization of quantitative bandgap metrology remains highly dependent on experimental conditions. Unlike conventional optical spectroscopy, determining local bandgap energies at the nanometer or atomic scale requires simultaneous optimization of energy and spatial resolution, specimen quality, acquisition parameters, and spectral analysis procedures. Small variations in any of these factors can significantly influence the observed onset behavior and, consequently, the extracted bandgap value.

The development of electron monochromators has played a particularly important role in enabling local bandgap measurements by dramatically improving energy resolution in STEM-EELS experiments. Prior to the introduction of monochromated electron sources, the energy spread of conventional field-emission guns often exceeded several hundred meV, making it difficult to distinguish weak interband-transition signals from the tail of the zero-loss peak (ZLP). Modern monochromated STEM-EELS systems have largely overcome this limitation and now routinely achieve energy resolutions below 100 meV, enabling reliable bandgap measurements in semiconductors, oxides, and low-dimensional materials.

In addition to energy resolution, quantitative bandgap determination requires careful consideration of specimen thickness, accelerating voltage, convergence and collection angles, detector performance, and ZLP subtraction methodology. These experimental factors influence both the signal-to-noise ratio and the magnitude of unwanted spectral contributions such as plural scattering, Cherenkov radiation, guided optical modes, and instrumental broadening. Consequently, the measured bandgap should be regarded not merely as a material property but as the outcome of a complete measurement workflow.

This section discusses the key experimental requirements for quantitative local bandgap measurements using monochromated STEM-VEELS, including instrumentation, specimen preparation, acquisition conditions, and spectral-processing strategies.

### Instrumentation requirements for monochromated STEM-VEELS

The most critical instrumental requirement for local bandgap measurements is high energy resolution. Because interband-transition onsets typically occur within the first few electron volts of the low-loss spectrum, the ability to distinguish weak bandgap-related signals from the intense zero-loss peak (ZLP) is fundamentally determined by the energy spread of the incident electron beam. Early high-energy-resolution EELS (HREELS) studies demonstrated that improving energy resolution substantially enhanced the analysis of low-loss electronic excitations and near-edge fine structures (Lazar et al. 2003). The subsequent development of dedicated electron monochromators further reduced the intrinsic energy spread of the incident beam to the few-tens-of-meV range, enabling not only quantitative bandgap measurements but also vibrational spectroscopy with unprecedented energy resolution (Krivanek et al. 2014). These studies highlighted the importance of improving energy resolution for low-loss spectroscopy and helped motivate subsequent developments in monochromated STEM-EELS instrumentation.

Electron monochromators are designed to reduce the intrinsic energy spread of electrons emitted from the source before they interact with the specimen. Among commercially available systems, two monochromator geometries are most widely used: Wien-filter-type and Omega-type monochromators, as illustrated in Fig. 1a.

In a Wien-filter monochromator, crossed electric and magnetic fields are used to disperse electrons according to their kinetic energy. An energy-selecting slit positioned at the dispersion plane allows only electrons within a narrow energy range to pass through the optical system. By rejecting electrons with energies outside the selected range, the overall energy spread of the beam can be significantly reduced.

Omega-type monochromators employ a series of magnetic prisms that force electrons to travel along an Ω-shaped trajectory. Because electrons with different kinetic energies follow slightly different paths, an energy-selective slit can again be used to isolate a narrow energy distribution. Omega-type systems are often capable of achieving extremely high energy resolution while maintaining excellent beam stability and probe-forming performance.

The monochromated electron beam subsequently passes through the aberration-corrected STEM column, where a sub-angstrom probe is formed on the specimen. Following interaction with the sample, elastically and inelastically scattered electrons are separated according to scattering angle and energy loss. High-angle scattered electrons are collected by the high-angle annular dark-field (HAADF) detector to provide structural information, while transmitted electrons enter the EELS spectrometer, where a magnetic prism disperses electrons according to their energy loss.

The practical impact of monochromation is illustrated in Fig. 1b, which shows a representative ZLP acquired at an accelerating voltage of 80 kV with an energy resolution of approximately 0.12 eV, defined as the full width at half maximum (FWHM) of the ZLP (Jung et al. 2013). Such energy resolution is sufficient to resolve subtle onset variations in many technologically important semiconductors and provides the fundamental basis for quantitative local bandgap determination.

Although energy resolution is often considered the primary figure of merit, spatial resolution must also be considered simultaneously. The ultimate capability of STEM-VEELS arises from the combination of sub-nanometer spatial resolution and sub-100 meV energy resolution, enabling direct correlation between local electronic structure and nanoscale morphology. Consequently, modern monochromated aberration-corrected STEM-EELS systems provide a unique platform for quantitative bandgap measurements unattainable with conventional optical techniques.

In practice, an energy resolution better than approximately 100–150 meV is generally desirable for reliable bandgap determination, particularly for narrow-bandgap semiconductors and heterostructures exhibiting the small local variations in electronic structure. Achieving this performance requires careful optimization of monochromator alignment, spectrometer stability, source brightness, and detector calibration. These considerations form the instrumental foundation upon which all subsequent acquisition and analysis procedures are built.

### Specimen preparation and thickness effects

Specimen preparation represents one of the most critical factors influencing the accuracy of local bandgap measurements in monochromated STEM-VEELS. Unlike conventional structural imaging, where moderately thick specimens may still provide useful information, quantitative low-loss EELS measurements are highly sensitive to specimen thickness because multiple scattering events can significantly distort the spectral onset region used for bandgap determination.

As an incident electron traverses a specimen, it may undergo multiple inelastic scattering events before exiting the material. The measured low-loss spectrum therefore contains not only the desired single-scattering distribution associated with interband transitions but also contributions arising from plural scattering. These additional scattering events broaden spectral features and can obscure the true onset of the bandgap signal. Consequently, increasing specimen thickness generally leads to greater uncertainty in bandgap extraction and may introduce systematic errors in the measured bandgap energy (Egerton 2011; Batson and Silcox 1983).

The influence of specimen thickness becomes particularly important for materials exhibiting weak onset signals or narrow bandgaps. In such systems, even modest spectral broadening may significantly alter the apparent onset position. Although Fourier-log deconvolution and related procedures can partially recover the single-scattering distribution, uncertainties in thickness estimation and deconvolution often persist. Therefore, minimizing specimen thickness is generally preferable to relying solely on post-processing corrections.

In addition to plural scattering, specimen thickness influences several other artifacts discussed in Sect. "Sources of uncertainty and spectral artifacts in local bandgap measurements". Relativistic-loss contributions such as Cherenkov radiation and guided optical modes tend to become more pronounced as the interaction volume increases. Similarly, thicker specimens increase the probability of delocalized excitations and may enhance unwanted low-loss intensity unrelated to direct interband transitions. As a result, reducing specimen thickness simultaneously improves spectral fidelity and minimizes multiple sources of uncertainty.

For most semiconductor and oxide materials, specimen thicknesses below approximately one inelastic mean free path (t/λ < 1) are generally recommended for quantitative low-loss EELS analysis. In practice, local bandgap measurements are frequently performed on specimens with thicknesses ranging from approximately 20 to 80 nm, depending on material composition, accelerating voltage, and specimen geometry. However, the optimum thickness remains material dependent and should be evaluated together with the expected scattering cross-section and signal-to-noise requirements.

The specimen preparation method itself can also influence bandgap measurements. Focused ion beam (FIB) milling is widely used for preparing electron-transparent specimens but may introduce surface amorphization, implantation damage, defect formation, or compositional modification. These preparation-induced artifacts can alter the local electronic structure and potentially shift the measured bandgap. Consequently, low-energy ion polishing, plasma cleaning, and careful final thinning procedures are often employed to minimize preparation-related damage prior to STEM-VEELS acquisition.

Another important consideration is specimen uniformity. Thickness gradients, surface contamination, oxidation, and preparation-induced defects may produce spatial variations in the measured low-loss spectrum that are unrelated to intrinsic electronic-structure changes. Therefore, complementary characterization techniques such as HAADF-STEM imaging, thickness mapping, diffraction analysis, or compositional mapping are frequently used to verify specimen quality before quantitative bandgap analysis.

Ultimately, reliable local bandgap measurements require specimens that are not only sufficiently thin for minimizing plural scattering but also structurally and chemically representative of the material being investigated. Careful specimen preparation, therefore, forms a critical foundation for quantitative STEM-VEELS bandgap metrology and directly influences the reliability of all subsequent acquisition and analysis procedures.

### Acquisition parameters: accelerating voltage, convergence angle, and collection angle

The reliability of local bandgap measurements depends not only on the performance of the microscope and specimen quality but also on the selection of acquisition parameters during STEM-EELS experiments. Among these parameters, the accelerating voltage, convergence angle, and collection angle play particularly important roles because they directly influence the balance between spatial resolution, energy resolution, signal intensity, and unwanted relativistic-loss contributions.

The accelerating voltage determines the velocity of the incident electron and therefore influences both the interaction volume and the probability of relativistic-loss excitations. Historically, low-loss EELS measurements were commonly performed at accelerating voltages of 200–300 kV due to improved beam penetration and reduced chromatic aberration. However, higher accelerating voltages also increase the likelihood of Cherenkov radiation and related relativistic-loss processes in high-refractive-index materials. As discussed in Sect. "Sources of uncertainty and spectral artifacts in local bandgap measurements", these additional electromagnetic excitations can introduce low-loss intensity unrelated to interband transitions and may distort the apparent bandgap onset. Consequently, lower accelerating voltages, typically in the range of 60–120 kV, are often preferred for quantitative local bandgap measurements when specimen stability permits.

The convergence semi-angle controls the angular distribution of the incident probe and influences both probe size and momentum transfer. Larger convergence angles generally improve spatial resolution by producing a smaller probe diameter, enabling nanoscale or atomic-scale localization of electronic-structure measurements. However, increasing the convergence angle also broadens the range of momentum transfer sampled during the experiment and may complicate the interpretation of low-loss spectra, particularly in anisotropic materials or systems exhibiting strong momentum-dependent excitations. Therefore, convergence angles should be selected to provide sufficient spatial resolution while maintaining acceptable spectral interpretability.

The collection semi-angle determines the range of scattered electrons accepted by the EELS spectrometer and is often one of the most critical parameters in low-loss EELS. Small collection angles preferentially sample low-momentum-transfer excitations and can enhance contributions from Cherenkov radiation, guided optical modes, and other long-range electromagnetic interactions. In contrast, larger collection angles suppress these relativistic-loss contributions and increase the relative contribution of localized electronic excitations. Consequently, optimization of the collection angle can significantly improve the accuracy of local bandgap measurements.

Particularly effective suppression of relativistic-loss artifacts can be achieved by acquiring off-axis EELS. In this approach, the spectrometer entrance aperture is intentionally displaced from the transmitted beam, reducing the collection of low-angle electromagnetic scattering events while retaining a substantial fraction of the interband-transition signal. Several studies have demonstrated that off-axis EELS can significantly reduce Cherenkov-related contributions and improve the reliability of bandgap measurements in high-refractive-index semiconductors (Stöger-Pollach 2008; Vatanparast et al. 2017).

Energy dispersion and dwell time also influence spectral quality. Smaller energy dispersion values increase sampling density in the low-loss region and improve onset determination but may reduce the accessible energy range. Similarly, longer dwell times improve the signal-to-noise ratio but increase the risk of beam-induced damage, contamination, and specimen drift. Therefore, optimization of acquisition parameters often requires balancing spectral precision against specimen stability.

In practice, no universal acquisition condition exists for all materials. Instead, the optimum experimental parameters depend on the specimen thickness, bandgap energy, refractive index, beam sensitivity, and desired spatial resolution. Consequently, quantitative STEM-VEELS bandgap measurements should be regarded as a multidimensional optimization problem in which accelerating voltage, convergence angle, collection angle, energy dispersion, and acquisition time are carefully adjusted to maximize the accuracy and reproducibility of the extracted bandgap value.

### Zero-loss peak removal and spectral processing

Accurate determination of local bandgap energies from monochromated STEM-VEELS spectra critically depends on the reliable removal of the zero-loss peak (ZLP). Although monochromation significantly improves energy resolution, the intensity of the ZLP typically remains several orders of magnitude larger than the weak interband-transition signal associated with the bandgap onset. Consequently, even small inaccuracies in ZLP subtraction can produce substantial errors in the extracted bandgap value. The ZLP consists primarily of unscattered electrons and elastically scattered electrons that retain their initial energy after traversing the specimen. Because the ZLP possesses a finite width and extended tails arising from instrumental broadening, source instability, monochromator alignment, spectrometer response, and specimen-dependent broadening, its intensity often overlaps with the energy range where interband transitions begin to appear. This overlap is particularly problematic for narrow-bandgap semiconductors and materials exhibiting weak onset signals, where the bandgap signature may be partially obscured by the ZLP tail.

**Fig. 4:**
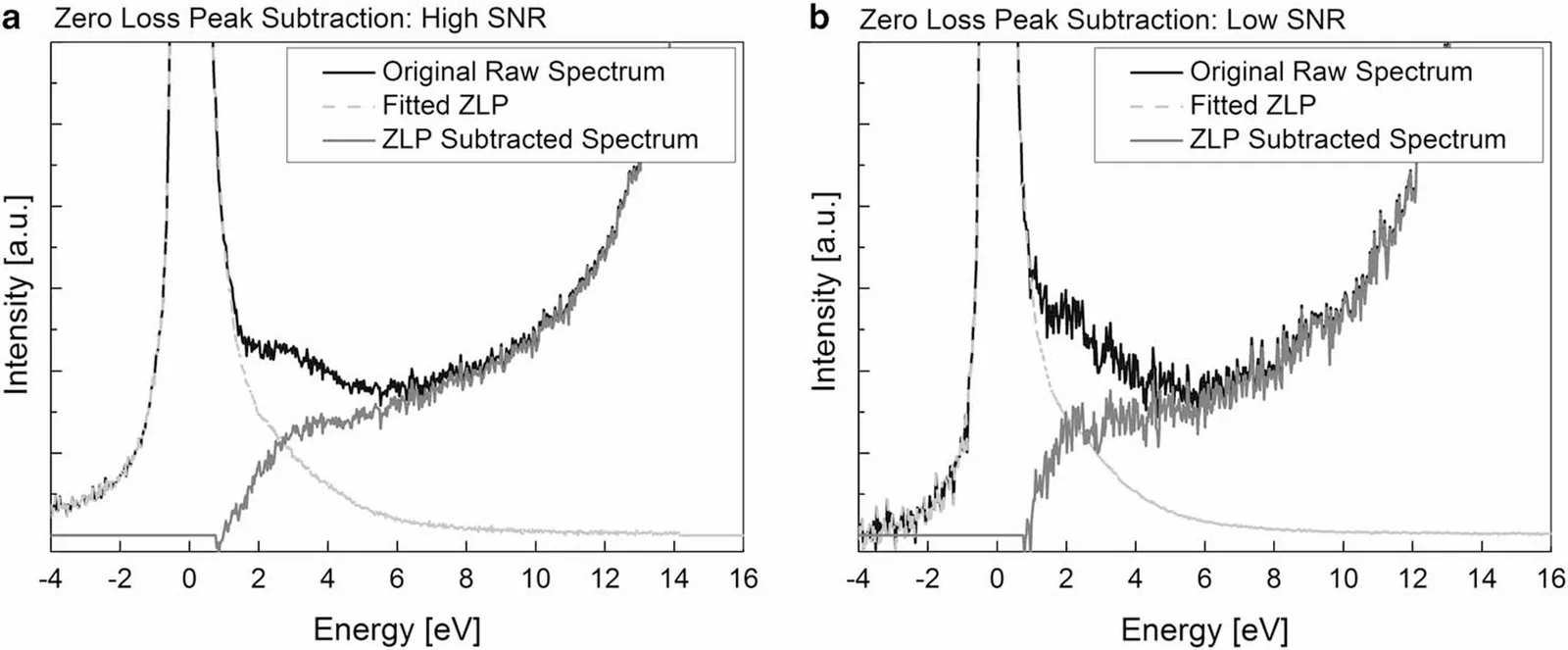
Conventional zero-loss peak subtraction using a vacuum reference. Representative examples of ZLP subtraction for (**a**) a high-SNR spectrum and (**b**) a low-SNR spectrum, illustrating the importance of spectral quality for reliable bandgap extraction. Adapted from Ref. (Keller et al. 2014)

Several conventional approaches (summarized in Table 8) have been developed to model and remove the ZLP contribution. One of the simplest methods involves acquiring a reference ZLP in vacuum and subsequently subtracting or deconvolving it from the specimen spectrum, shown in Fig. 4. While straightforward, this method assumes that the ZLP measured in vacuum accurately represents the effective ZLP shape within the specimen. In practice, however, specimen thickness, plural scattering, phonon scattering, surface losses, and other specimen-related broadening mechanisms may modify the ZLP profile, leading to systematic errors in the reconstructed low-loss spectrum (Keller et al. 2014; Roest et al. 2021).

**Table 8:** Comparison of ZLP-removal methods in STEM-VEELS bandgap analysis

Method	Advantages	Limitations
Vacuum reference	Direct experimental reference	May not represent specimen-broadened ZLP
Reflected tail	Fast	Assumes symmetry
Gaussian/Lorentzian fit	Flexible	Model dependent
Fourier-log deconvolution	Removes plural scattering	Noise amplification
Richardson–Lucy	High resolution	Over-iteration artifacts
ML-based fitting	Automated ZLP estimation, uncertainty propagation, spectrum-image analysis	Training-data dependence; validation and interpretability required

**Fig. 5:**
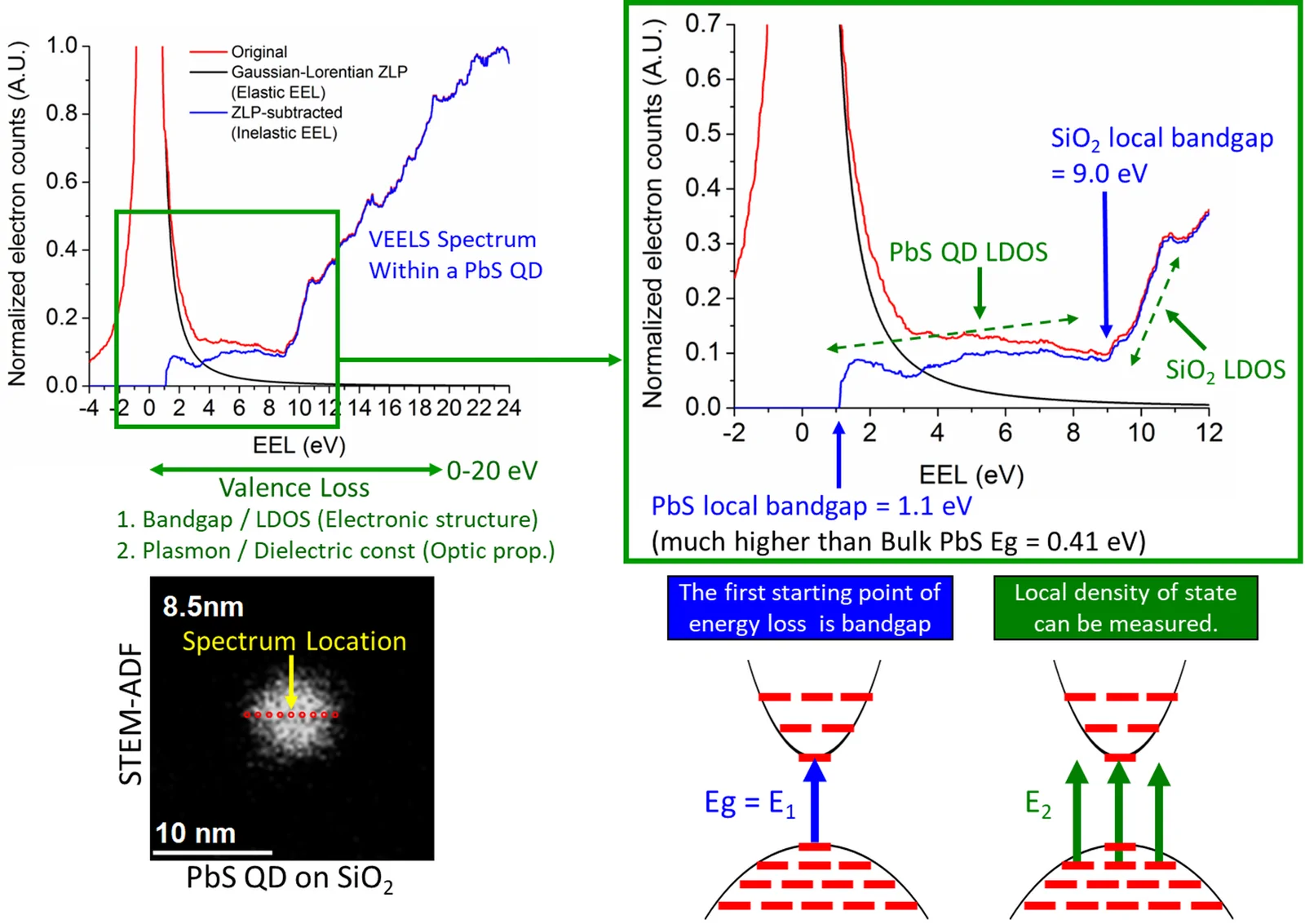
Representative monochromated STEM-VEELS spectrum acquired from the center of a dome-shaped PbS quantum dot (QD) deposited on an 8 nm amorphous SiO_2_ substrate at an accelerating voltage of 80 kV. The zero-loss peak (ZLP) was modeled using a Gaussian–Lorentzian function and subtracted to obtain the inelastic low-loss spectrum. The onset of the first interband transition was used to determine the local bandgap, while higher-energy loss features were assigned to the local density of states (LDOS) and dielectric response. The measured local bandgaps are approximately 1.1 eV for the PbS QD and 9.0 eV for the surrounding SiO_2_. The enlarged bandgap of the PbS QD relative to bulk PbS (0.41 eV) originates from quantum confinement. The schematic illustrates the relationship between the bandgap onset, subsequent LDOS-related transitions, and the measured VEELS spectrum. Reproduced from ref. (Jung et al. 2013)

A commonly used alternative is reflected-tail subtraction, in which the negative-energy side of the ZLP is mirrored about the zero-loss position and used to estimate the positive-energy tail. This approach is computationally simple and can provide satisfactory results when the ZLP is sufficiently symmetric. However, asymmetries arising from source fluctuations, spectrometer aberrations, or specimen-induced broadening may limit its accuracy. Model-based fitting approaches have also been widely employed, where the ZLP is represented using analytical functions such as Gaussian, Lorentzian, Gaussian-Lorentzian, pseudo-Voigt, Pearson, power-law, or combinations thereof. Figure 5 illustrates how the onset of the low-loss spectrum, as determined by the Gaussian-Lorentzian model for ZLP, corresponds to the local bandgap, whereas higher-energy spectral features reflect the local electronic density of states and dielectric response (Jung et al. 2013). Although these models can reproduce the central ZLP and its tail under selected conditions, the resulting bandgap value may depend on the assumed functional form and the selected fitting range (Roest et al. 2021).

Deconvolution techniques provide another important class of spectral-processing methods. Fourier-log deconvolution is commonly used to remove plural scattering contributions and recover the single-scattering distribution, while Richardson–Lucy deconvolution and related iterative algorithms have been applied to enhance spectral resolution and suppress instrumental broadening. These methods can improve the visibility of weak onset features, but they may also amplify spectral noise or introduce reconstruction artifacts if applied excessively. Keller et al. demonstrated that the reliability of local bandgap measurements depends strongly on the signal-to-noise ratio, background correction, and extraction strategy, showing that low-SNR spectra can generate artifacts during bandgap extraction even when ZLP subtraction is formally applied (Keller et al. 2014).

**Fig. 6:**
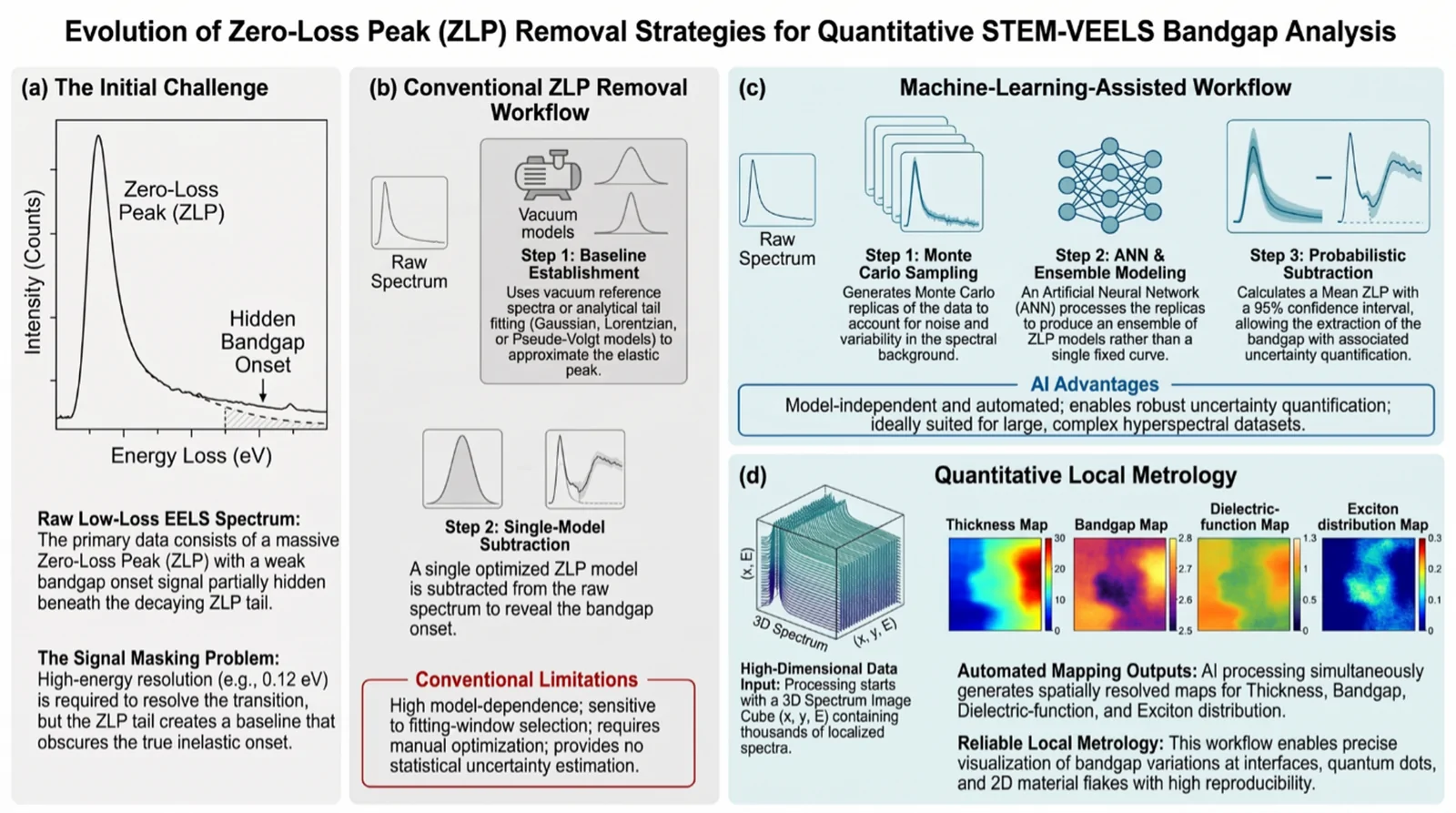
Evolution of zero-loss peak (ZLP) removal strategies for quantitative local bandgap analysis using monochromated STEM-VEELS. (**a**) The intense ZLP masks the weak onset of interband transitions, making accurate bandgap determination challenging. (**b**) Conventional ZLP-removal methods estimate the elastic contribution using vacuum-reference spectra or analytical fitting functions (e.g., Gaussian, Lorentzian, pseudo-Voigt, and Pearson models) to construct a single ZLP model prior to subtraction. Although widely adopted, these approaches are generally sensitive to the choice of fitting model and fitting window and do not explicitly quantify the uncertainty associated with the extracted bandgap. (**c**) Recent machine-learning-assisted approaches, exemplified by the EELSfitter framework, employ Monte Carlo sampling together with artificial neural networks (ANNs) to generate an ensemble of statistically equivalent ZLP models, enabling probabilistic background subtraction and uncertainty-aware bandgap extraction (Roest et al. 2021). (**d**) The machine-learning workflow can be further extended to monochromated STEM-EELS spectrum-image datasets, enabling automated reconstruction of spatially resolved maps of thickness, bandgap, dielectric function, and excitonic properties for quantitative local electronic-structure characterization (Brokkelkamp et al. 2022). This schematic summarizes the transition from conventional model-dependent ZLP subtraction to modern data-driven, uncertainty-aware spectral reconstruction for quantitative STEM-VEELS bandgap metrology

Advances in machine learning have introduced a new class of ZLP-removal strategies that directly address several limitations of conventional fitting. As summarized in Fig. 6, ZLP-removal strategies have evolved from conventional model-based fitting toward data-driven, uncertainty-aware frameworks for quantitative bandgap analysis. Traditional methods generally require explicit assumptions about the ZLP shape, the fitting window, and the peak’s degree of symmetry. These assumptions can introduce methodological bias, and in many cases, the associated uncertainty is difficult to propagate into the final bandgap value.

Roest et al. introduced EELSfitter as a machine-learning-based framework for model-independent ZLP estimation in the low-loss EELS region. In this approach, artificial neural networks serve as flexible interpolators to parameterize the ZLP, while Monte Carlo replica sampling is used to construct a probability distribution over possible ZLP backgrounds. This strategy enables not only ZLP subtraction but also propagation of the associated uncertainty into the extracted low-loss features (Roest et al. 2021). As illustrated in Fig. 6c, the method generates an ensemble of statistically equivalent ZLP models rather than a single deterministic background, enabling probabilistic background subtraction and uncertainty-aware bandgap extraction.

The significance of EELSfitter is that it treats ZLP subtraction as a statistical inference problem rather than a deterministic curve-fitting problem. Instead of producing a single background curve, the method generates an ensemble of plausible ZLP models, from which confidence intervals can be estimated. This is particularly valuable for local bandgap metrology because the uncertainty of the ZLP tail directly affects the uncertainty of the extracted onset energy. Applied to polytypic WS₂ nanoflowers, the method enabled robust identification of excitonic transitions in the ultra-low-loss region and extraction of an indirect bandgap of approximately 1.6 eV with an explicit uncertainty interval (Roest et al. 2021). The same framework has subsequently been extended to spectrum-image datasets for automated mapping of local bandgap, dielectric function, and specimen thickness (Brokkelkamp et al. 2022), as schematically illustrated in Fig. 6d.

This machine-learning strategy was subsequently extended to spatially resolved EELS spectrum images by Brokkelkamp et al. In spectrum-image datasets, each pixel can exhibit a different ZLP background because the local thickness, scattering probability, and specimen environment vary across the field of view. To address this problem, individual spectra were first grouped by thickness using K-means clustering, after which a deep learning model was trained to parameterize the ZLP background as a function of energy loss and integrated spectral intensity. The resulting ZLP-subtracted spectrum images enabled spatially resolved determination of specimen thickness, bandgap energy, and complex dielectric function in two-dimensional materials such as InSe flakes and polytypic WS₂ nanoflowers (Brokkelkamp et al. 2022). This development is important because it transforms ZLP subtraction from a spectrum-by-spectrum manual procedure into a reproducible, uncertainty-aware, hyperspectral analysis workflow. In particular, Brokkelkamp et al. showed that EELS spectral images can be treated as three-dimensional data cubes, in which the spatial coordinates and the energy-loss axis are analyzed jointly. After ZLP subtraction, the same dataset can be used not only for bandgap mapping but also for Kramers–Kronig analysis and dielectric-function reconstruction, linking local structural variation, thickness, bandgap energy, and optical response within a single analysis framework. The broader applicability of this approach has been demonstrated in subsequent studies. In 1D MoS₂ nanostructures, machine-learning-assisted EELS analysis was used to map localized excitonic features and spatial bandgap modulation associated with curvature-induced strain fields, revealing excitonic peaks around 2 and 3 eV and a spatially varying bandgap near the nanostructure endpoints (van der Lippe et al. 2023). Although this type of application is discussed in more detail in later sections, it illustrates why robust ZLP subtraction is not merely a preprocessing step but a prerequisite for extracting spatially meaningful electronic-structure information from low-loss VEELS datasets. Machine-learning-assisted analysis has also been extended beyond conventional energy-loss spectra. For example, La et al. applied uncertainty-aware ZLP modeling to energy-gain EELS in Bi₂Te₃, identifying edge-enhanced collective excitations around − 0.8 eV and demonstrating that Monte Carlo replica methods can be used to evaluate the robustness and statistical significance of weak spectral features (La et al. 2023). Although energy-gain EELS is distinct from bandgap onset determination, this work further demonstrates the potential of EELSfitter-type frameworks for automated, uncertainty-aware analysis of weak low-energy excitations.

Following ZLP removal, additional preprocessing steps are often performed before bandgap extraction. These may include energy calibration using the ZLP position, spectral alignment, denoising, thickness correction, plural-scattering removal, normalization, and masking of vacuum or substrate regions. Because each processing step can influence the apparent onset position, the complete analysis workflow should be carefully documented to ensure reproducibility and meaningful comparisons between studies. Ultimately, ZLP subtraction should not be regarded as a routine preprocessing step but rather as a critical component of quantitative bandgap metrology. The choice of ZLP-removal strategy directly influences the visibility of low-energy interband transitions and may introduce uncertainties that are comparable to or larger than the instrument’s intrinsic energy resolution. Machine-learning-based approaches such as EELSfitter provide an important path toward automated, reproducible, and uncertainty-aware local bandgap extraction, particularly for large spectrum-image datasets where manual fitting is impractical. As monochromated STEM-VEELS continues to move toward quantitative nanoscale metrology, such frameworks are expected to play an increasingly central role in standardizing ZLP subtraction, bandgap mapping, and dielectric-function analysis.

## Applications of monochromated STEM-VEELS for local bandgap measurements

The development of monochromated STEM-EELS has transformed local bandgap measurements from a proof-of-concept technique into a versatile analytical tool for investigating nanoscale electronic structures across a wide range of materials. By combining sub-100 meV energy resolution with nanometer- or even atomic-scale spatial resolution, monochromated STEM-VEELS enables direct correlation between local electronic properties and structural, compositional, or morphological variations that cannot be resolved by conventional optical spectroscopies.

Applications of local bandgap measurements have expanded rapidly from bulk semiconductor validation to quantum-confined nanostructures, semiconductor heterostructures, two-dimensional materials, and complex functional systems. These studies have demonstrated that STEM-VEELS can reveal spatial variations in electronic structure arising from quantum confinement, strain, composition, interfaces, defects, and reduced dimensionality. This section summarizes representative applications of monochromated STEM-VEELS and highlights how advances in instrumentation and spectral analysis have enabled quantitative local bandgap metrology across increasingly complex material systems.

### Quantum-confined nanostructures: local bandgap and plasmonic excitations

Quantum-confined semiconductor nanostructures provide an ideal platform for demonstrating the capability of monochromated STEM-VEELS for local bandgap measurements. Unlike bulk semiconductors, the electronic structure of quantum dots varies significantly over only a few nanometers owing to quantum confinement, making them difficult to investigate using conventional optical spectroscopies, which are inherently limited by diffraction and ensemble averaging. The combination of high spatial resolution and sub-100 meV energy resolution enables monochromated STEM-VEELS to directly probe local variations in electronic structure within individual nanostructures.

**Fig. 7:**
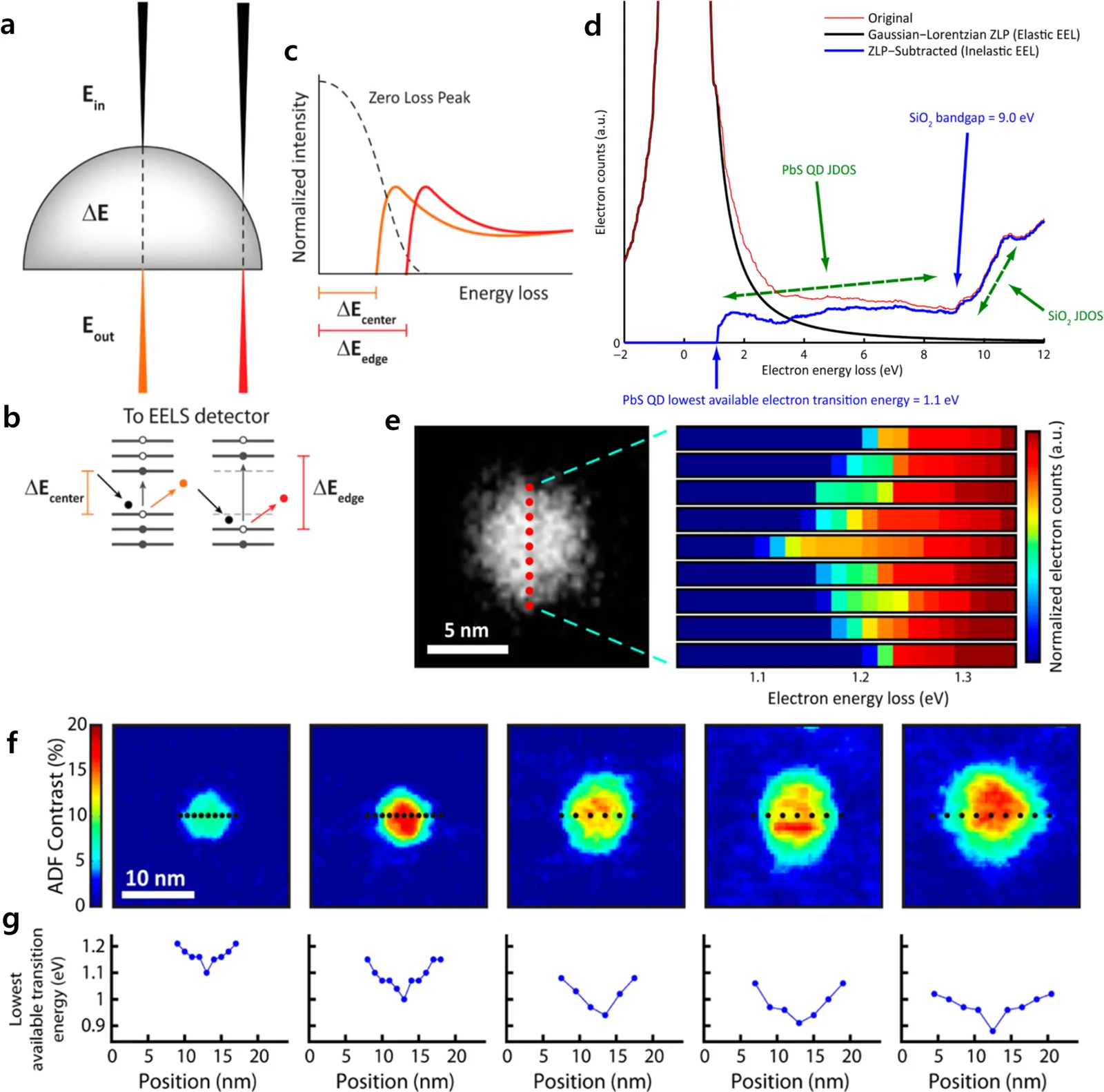
Local bandgap variations in individual PbS quantum dots (QDs) measured by monochromated STEM-VEELS. (**a**) Schematic illustration of the STEM-VEELS measurement geometry used to probe the local electronic structure of a single PbS QD deposited on an amorphous SiO_2_ substrate. (**b**) Schematic energy-level diagrams illustrating the increase in the lowest available electronic transition energy (local bandgap) from the center toward the edge of the QD as a consequence of quantum confinement. (**c**) Conceptual illustration of the relationship between the zero-loss peak (ZLP) tail and the onset of interband transitions, demonstrating the increase in the local bandgap from the QD center to the edge following ZLP subtraction. (**d**) Representative low-loss EEL spectrum acquired from the center of a PbS QD after Gaussian–Lorentzian ZLP fitting and subtraction, showing a local bandgap of approximately 1.1 eV for the PbS QD and ~ 9.0 eV for the surrounding SiO_2_. The higher-energy spectral features originate from interband transitions associated with the joint density of states (JDOS). (**e**) HAADF-STEM image indicating the positions used for spatially resolved spectrum acquisition together with the corresponding low-loss EEL spectra acquired across an individual PbS QD, demonstrating the evolution of the local bandgap with position. (**f**) HAADF-STEM images of PbS QDs with different diameters. (**g**) Corresponding local bandgap profiles measured across the individual QDs shown in (**f**), demonstrating that the lowest available transition energy increases from the center toward the edge of each QD and becomes larger with decreasing particle size owing to quantum confinement. Reproduced from Ref. (Jung et al. 2013),

PbS quantum dots have attracted considerable interest because their bandgap strongly depends on particle size as a consequence of quantum confinement. As the size of the quantum dot decreases, confinement of electron and hole wavefunctions increases the energy separation between the highest occupied molecular orbital (HOMO) and the lowest unoccupied molecular orbital (LUMO), resulting in a substantial increase in the bandgap relative to bulk PbS. Therefore, PbS quantum dots provide a representative model system for evaluating the capability of monochromated STEM-VEELS to measure local bandgap variations with nanometer-scale spatial resolution. Figure 7 summarizes representative local bandgap measurements performed on individual PbS quantum dots using monochromated STEM-VEELS (Jung et al. 2013). The figure combines the measurement principle, representative low-loss spectra following ZLP subtraction, HAADF-STEM imaging, spatially resolved spectral analysis, and the measured size dependence of the local bandgap. Unlike optical spectroscopy, which measures the ensemble-averaged response from a large number of nanoparticles, monochromated STEM-VEELS directly correlates local morphology with local electronic structure within a single quantum dot, enabling quantitative evaluation of quantum-confinement effects.

The measured local bandgap of approximately 1.1 eV for individual PbS quantum dots was significantly larger than the bulk value of 0.41 eV, consistent with the expected bandgap widening caused by quantum confinement (Jung et al. 2013). In addition to the bandgap onset, higher-energy loss features were assigned to transitions associated with the local density of states (LDOS), demonstrating that monochromated STEM-VEELS provides broader information on the local electronic structure beyond simple bandgap determination. Furthermore, spatially resolved measurements across individual quantum dots revealed systematic variations in the electronic structure that could be directly correlated with particle size and morphology. These results demonstrate the capability of monochromated STEM-VEELS to quantitatively investigate local electronic structures in individual quantum-confined nanostructures and establish a foundation for subsequent applications to semiconductor heterostructures, interfaces, and other low-dimensional materials.

**Fig. 8:**
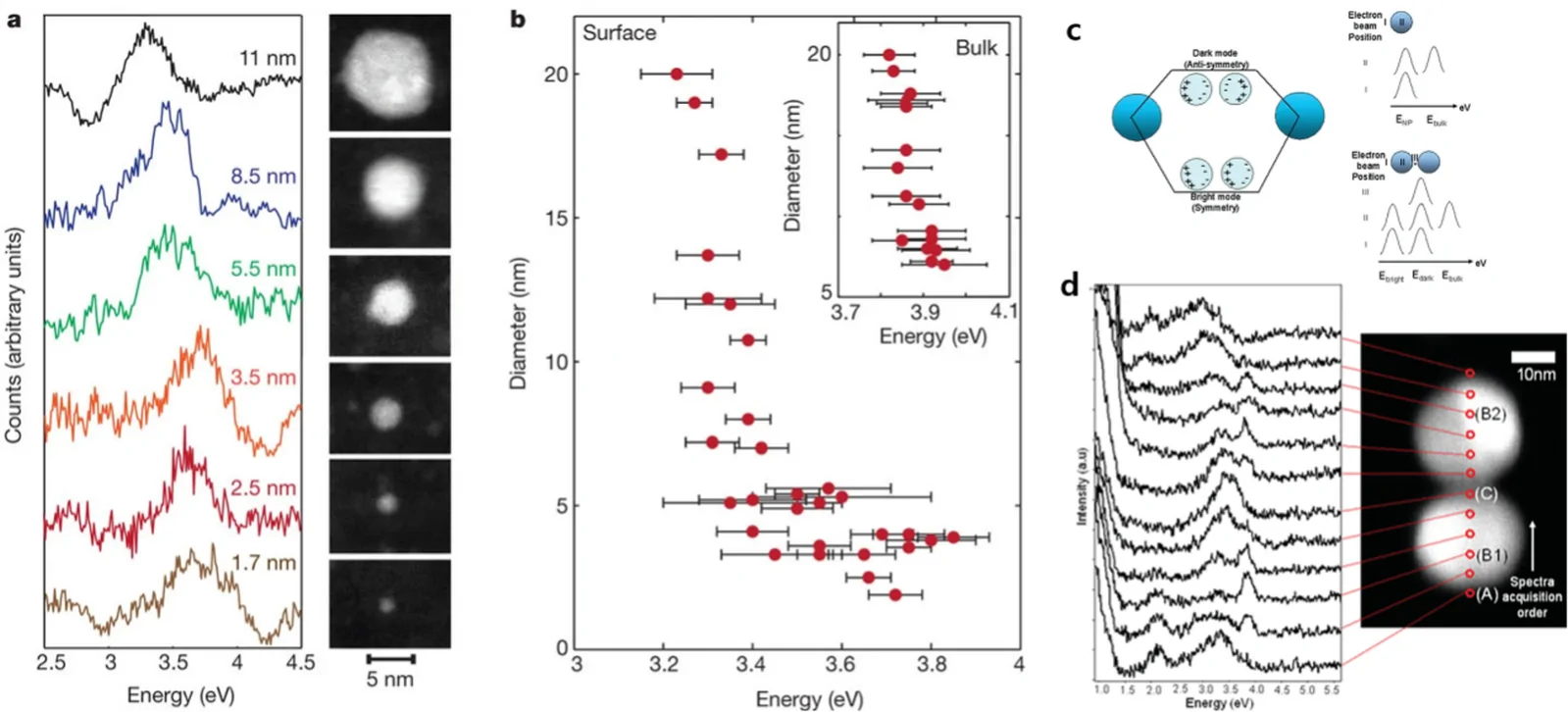
Monochromated STEM-EELS investigation of localized surface plasmon resonances in metallic nanoparticles. (**a**) Representative normalized low-loss EEL spectra acquired from individual silver nanoparticles with diameters ranging from 1.7 to 11 nm, together with the corresponding STEM images. The electron probe was positioned near the particle edge to selectively excite localized surface plasmon resonances. (**b**) Dependence of the surface plasmon resonance energy on nanoparticle diameter, demonstrating a systematic blue shift of the resonance with decreasing particle size due to quantum confinement and nonlocal effects. (**c**) Schematic illustration of plasmon hybridization in coupled silver nanoparticle dimers, showing the formation of optically bright and dark plasmon modes under electron-beam excitation. (**d**) Spatially resolved monochromated STEM-EELS spectra acquired across a silver nanoparticle dimer, directly revealing the spatial distribution of dark plasmon modes that are inaccessible using conventional optical spectroscopy. These results demonstrate the unique capability of monochromated STEM-EELS to probe localized plasmonic excitations and mode hybridization with nanometer spatial resolution. Panels (**a**, **b**) reproduced from Ref. (Scholl et al. 2012). Panels (**c**, **d**) reproduced from Ref. (Koh et al. 2009)

In addition to semiconductor bandgap measurements, monochromated STEM-VEELS has also become a powerful technique for investigating localized surface plasmon resonances in metallic nanostructures. Unlike optical spectroscopy, which predominantly probes optically allowed bright modes, the highly localized electron probe can efficiently excite both radiative and non-radiative plasmon modes with nanometer-scale spatial resolution. This unique capability enables direct investigation of plasmon confinement, mode hybridization, and size-dependent resonance behavior within individual nanoparticles and coupled nanostructures. As shown in Fig. 8, Scholl et al. systematically investigated localized surface plasmon resonances in individual silver nanoparticles with diameters ranging from approximately 1.7 to 11 nm using monochromated STEM-EELS (Scholl et al. 2012). The surface plasmon resonance exhibited a clear size dependence, shifting toward higher energies as the nanoparticle diameter decreased because of quantum confinement and nonlocal electronic effects. These measurements demonstrated that monochromated STEM-VEELS can directly probe quantum plasmonic behavior at dimensions beyond the reach of conventional optical spectroscopy. Monochromated STEM-EELS also provides unique access to plasmon hybridization in coupled nanoparticle systems. Koh et al. employed spatially resolved STEM-EELS to investigate individual silver nanoparticles and nanoparticle dimers, demonstrating that the electron beam can directly excite both optically allowed bright modes and optically forbidden dark plasmon modes that arise from electromagnetic coupling between neighboring nanoparticles (Koh et al. 2009). As illustrated in Fig. 8, spectrum imaging across a nanoparticle dimer reveals the spatial distribution of these hybridized plasmon modes, providing direct experimental visualization of localized plasmon coupling with nanometer-scale spatial resolution. These studies highlight that monochromated STEM-VEELS serves as a versatile platform for probing not only interband transitions associated with local bandgaps but also localized plasmonic excitations in metallic nanostructures.

### Semiconductor interfaces and heterostructures

Semiconductor heterostructures consist of multiple layers with different compositions, crystal structures, and electronic properties, producing abrupt changes in the local band structure across buried interfaces. These interfaces play a crucial role in determining carrier transport, band alignment, optical emission, and overall device performance in optoelectronic and electronic devices. Therefore, direct measurement of local bandgap variations across semiconductor heterointerfaces is essential for understanding nanoscale electronic structures that cannot be resolved by conventional optical spectroscopies. Unlike isolated quantum dots, heterostructures present additional experimental challenges because the local bandgap must be measured across multiple buried layers while maintaining high spatial and energy resolution. Furthermore, wide-bandgap materials such as GaN- and AlN-based semiconductors are particularly susceptible to parasitic optical excitations, including Cherenkov radiation and guided optical modes, which can obscure the intrinsic interband-transition onset discussed in Sect. "Fundamentals of STEM-VEELS for local bandgap analysis". Consequently, optimizing the experimental acquisition geometry is an important prerequisite for reliable bandgap measurements.

**Fig. 9:**
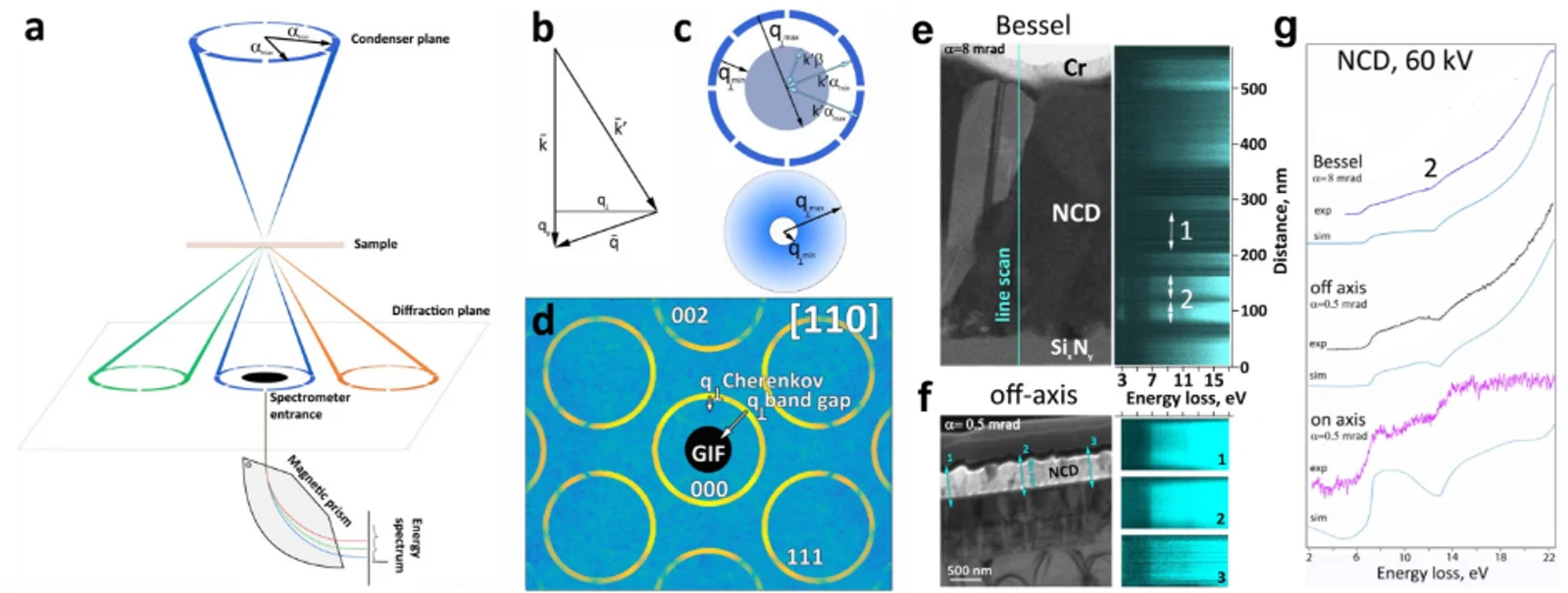
Comparison of advanced STEM-EELS acquisition geometries for quantitative local bandgap measurements in wide-bandgap semiconductor materials. (**a**) Schematic illustration of the Bessel-aperture STEM-EELS configuration, in which annular illumination combined with off-axis signal collection suppresses low-momentum-transfer radiative excitations. (**b**, **c**) Momentum-transfer diagrams illustrating the scattering vectors and q-space selection achieved by the Bessel geometry. (**d**) Simulated convergent-beam electron diffraction (CBED) pattern showing the exclusion of the low-q region associated with Cherenkov radiation while retaining the momentum-transfer range relevant for interband transitions. (**e**) Experimental Bessel STEM-EELS line-scan acquired from nanocrystalline diamond (NCD), demonstrating efficient suppression of radiative background while preserving the bandgap signal. (**f**) Conventional off-axis STEM-EELS measurement acquired under comparable conditions, illustrating partial suppression of Cherenkov radiation but with increased residual background compared with the Bessel configuration. (**g**) Comparison of experimental and simulated low-loss EEL spectra acquired using Bessel, conventional off-axis, and conventional on-axis geometries, demonstrating the progressive improvement in bandgap visibility as parasitic radiative losses are reduced. While conventional on-axis acquisition exhibits the strongest contribution from Cherenkov radiation, the off-axis geometry provides partial suppression, and the Bessel configuration achieves the most effective rejection of radiative excitations for reliable local bandgap determination. Reproduced from Ref. (Korneychuk et al. 2018a, b)

**Fig. 10:**
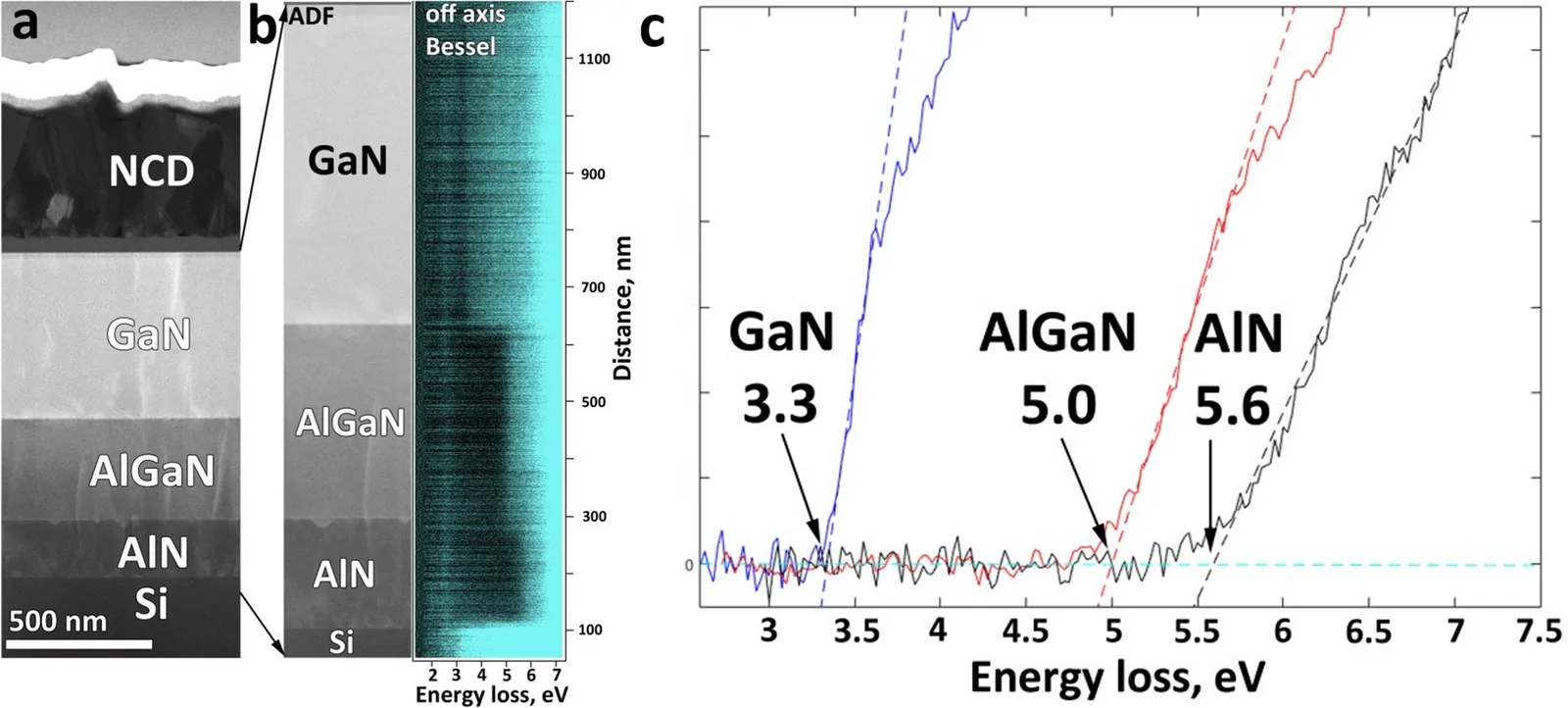
Quantitative local bandgap profiling across a multilayer GaN/AlGaN/AlN semiconductor heterostructure using off-axis Bessel STEM-EELS. (**a**) ADF-STEM image of a FIB-prepared cross-sectional specimen consisting of nanocrystalline diamond (NCD), GaN, AlGaN, AlN, and Si layers. (**b**) Spatially resolved STEM-VEELS line scan acquired in the off-axis Bessel configuration, demonstrating effective suppression of the zero-loss peak (ZLP) tail and enabling reliable bandgap profiling across the multilayer structure. (**c**) Representative low-loss EEL spectra acquired from the GaN, AlGaN, and AlN layers, with the bandgap onsets determined by linear fitting, yielding local bandgaps of approximately 3.3, 5.0, and 5.6 eV, respectively. These results demonstrate the capability of off-axis Bessel STEM-EELS to quantitatively resolve local bandgap variations across buried semiconductor heterostructures. Reproduced from Ref. (Korneychuk et al. 2018a, b)

To address these limitations, alternative STEM-EELS acquisition geometries have been developed to selectively suppress low-momentum-transfer radiative excitations while preserving the interband-transition signal. In conventional on-axis STEM-EELS, most incident electrons are concentrated near the optical axis and are therefore highly susceptible to forward-scattered radiative excitations, including Cherenkov radiation. A straightforward improvement is the off-axis acquisition geometry, in which the spectrometer entrance aperture is displaced from the transmitted beam, excluding electrons scattered at very small momentum transfers from detection. Because Cherenkov radiation is predominantly concentrated in this low-*q* region, off-axis acquisition substantially reduces its contribution while retaining a significant fraction of the interband-transition signal. A further improvement is provided by the Bessel-aperture STEM-EELS geometry. Instead of modifying only the collection geometry, the Bessel configuration employs an annular condenser aperture to generate a ring-shaped incident electron beam. As a result, low-momentum-transfer electrons are largely avoided during both illumination and collection, enabling more efficient suppression of Cherenkov radiation while maintaining sensitivity to intrinsic interband transitions. These acquisition geometries were experimentally validated using nanocrystalline diamond (NCD), a representative wide-bandgap material in which Cherenkov radiation strongly overlaps with the intrinsic interband-transition signal, making reliable bandgap determination particularly challenging. As illustrated in Fig. 9, the combination of annular illumination, momentum-space filtering, and off-axis signal collection significantly improves the visibility of bandgap onsets compared with conventional on-axis STEM-EELS (Korneychuk et al. 2018a, b). These methodological advances were subsequently applied to multilayer GaN/AlGaN/AlN heterostructures, enabling quantitative local bandgap profiling across buried semiconductor interfaces. As shown in Fig. 10, off-axis Bessel STEM-EELS enabled spatially resolved line-profile measurements across the multilayer structure, allowing distinct local bandgaps of approximately 3.3 eV for GaN, 5.0 eV for AlGaN, and 5.6 eV for AlN to be quantitatively resolved (Korneychuk et al. 2018a, b). These measurements demonstrate that monochromated STEM-VEELS can accurately determine bandgap discontinuities across buried semiconductor interfaces while simultaneously preserving nanometer-scale spatial resolution. Such capability provides direct experimental access to local band alignment in multilayer semiconductor devices, which is difficult to obtain using conventional optical techniques.

**Fig. 11:**
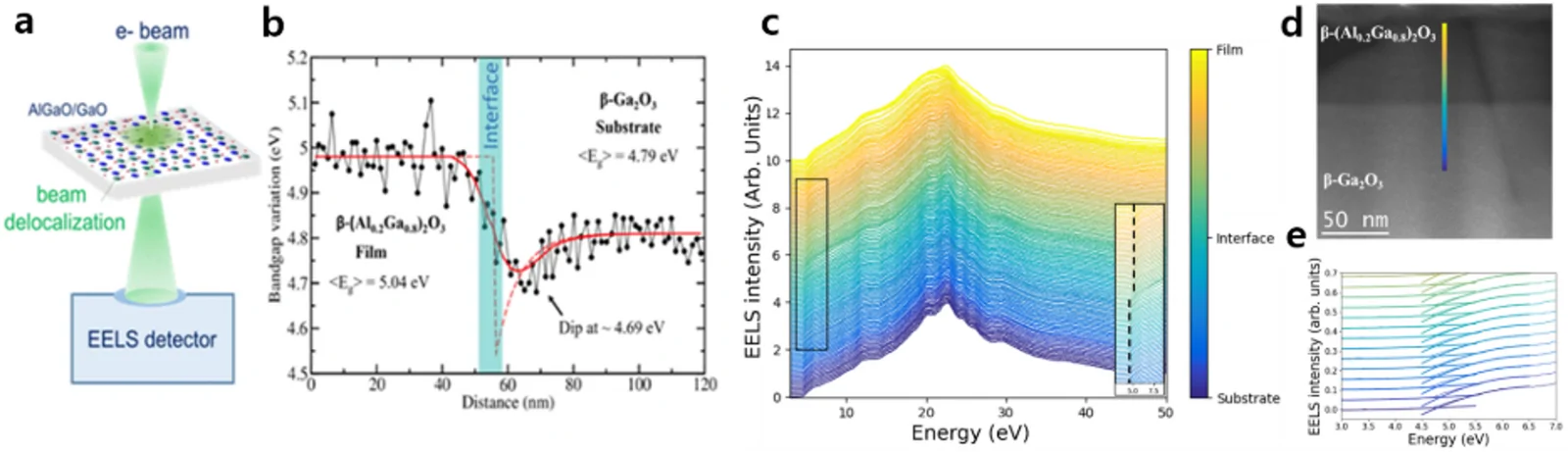
Spatially resolved STEM-VEELS analysis of bandgap distortion across a β-(Al_0.2_Ga_0.8_)_2_O_3_/β-Ga_2_O_3_ heterointerface. (**a**) Schematic illustration of the experimental concept, showing the influence of beam delocalization on local bandgap measurements across a buried semiconductor interface. (**b**) Quantitative local bandgap profile extracted from STEM-VEELS measurements, revealing a pronounced bandgap narrowing near the heterointerface. The experimental data (black symbols) are compared with a model incorporating an exponential bandgap transition convoluted with beam broadening, indicating an interface bandgap dip of approximately 0.25 eV arising from defect-related electronic states. (**c**) Spatially resolved low-loss EEL spectra acquired across the film/substrate interface, illustrating the gradual evolution of the bandgap onset. (**d**) HAADF-STEM image showing the β-(Al_0.2_Ga_0.8_)_2_O_3_ thin film, β-Ga_2_O_3_ substrate, and the corresponding STEM-VEELS line-scan position. (**e**) Enlarged view of the low-loss spectral region highlighting the systematic shift of the bandgap onset across the heterointerface. These results demonstrate that monochromated STEM-VEELS can quantitatively resolve subtle interface-induced bandgap distortions associated with buried defect states. Reproduced from Ref. (Chmielewski et al. 2022)

Complementary to determining bandgap discontinuities between adjacent layers, monochromated STEM-VEELS has also been applied to investigate subtle electronic-structure modifications induced by interface defects. Chmielewski et al. employed spatially resolved STEM-VEELS to study the β-(Al_0.2_Ga_0.8_)_2_O_3_/β-Ga_2_O_3_ heterointerface and observed a localized reduction in the bandgap near the buried interface (Chmielewski et al. 2022). As summarized in Fig. 11, analysis of the spatially resolved bandgap profile revealed a bandgap dip of approximately 0.25 eV that could not be explained solely by compositional averaging. Instead, after accounting for beam delocalization through convolution modeling, the observed bandgap narrowing was attributed to interface-related defect states. This work demonstrated that monochromated STEM-VEELS can detect subtle electronic-structure distortions associated with buried defects and interface states, extending its application beyond simple bandgap measurements toward quantitative nanoscale electronic-structure characterization.

These studies demonstrate that monochromated STEM-VEELS has evolved from measuring local bandgaps in individual quantum-confined nanostructures to quantitatively characterizing complex semiconductor heterostructures and buried interfaces. Advances in acquisition geometry, together with improvements in monochromator performance and spectral analysis, have significantly expanded the applicability of local bandgap measurements to technologically important wide-bandgap semiconductor systems. More importantly, these developments illustrate that monochromated STEM-VEELS can reveal not only spatial variations in bandgap energy but also interface-induced modifications to the electronic structure that are inaccessible to conventional characterization techniques. This capability provides the foundation for extending local bandgap measurements to atomically thin materials, where reduced dimensionality and interfacial interactions become even more pronounced.

### Low-dimensional materials (2D or 1D)

Following the successful application of monochromated STEM-VEELS to quantum-confined nanostructures and semiconductor heterostructures, recent studies have extended the technique to low-dimensional materials, where electronic properties become highly sensitive to atomic-scale structural variations. In two-dimensional (2D) materials, the reduction of dimensionality, strong dielectric confinement, and interlayer coupling give rise to electronic structures that differ substantially from those of their bulk counterparts. Consequently, local electronic excitations may vary over only a few nanometers due to changes in layer number, stacking sequence, strain, defects, or moiré superlattice formation. These characteristics make monochromated STEM-VEELS particularly attractive because it combines sub-nanometer spatial resolution with meV-scale energy resolution, enabling direct visualization of local electronic structures that are inaccessible using conventional optical spectroscopies.

**Fig. 12:**
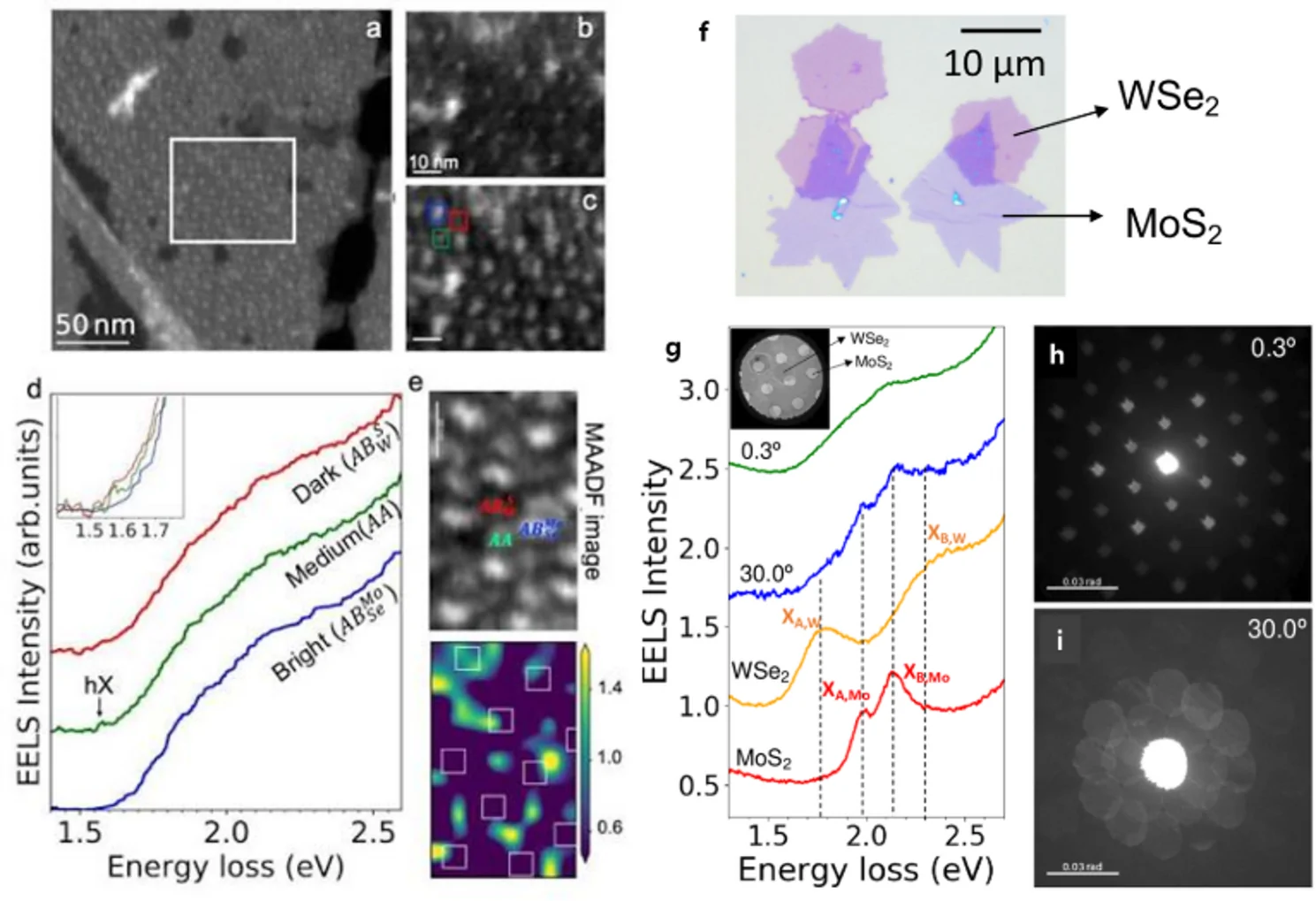
Monochromated STEM-VEELS analysis of local electronic excitations in twisted MoS_2_/WSe_2_ van der Waals heterobilayers. (**a**–**c**) Atomic-resolution MAADF-STEM images of the MoS_2_/WSe_2_ heterobilayer, showing the analyzed moiré region and representative local stacking configurations. (**d**) Low-loss EEL spectra acquired from different stacking sites within a moiré supercell, revealing stacking-dependent variations in low-energy electronic excitations. (**e**) MAADF-STEM image and corresponding map highlighting the spatial distribution of local stacking configurations. (**f**) Optical image of representative twisted MoS_2_/WSe_2_ heterobilayers. (**g**) Comparison of low-loss EEL spectra acquired from isolated MoS₂, isolated WSe_2_, and twisted heterobilayers with different twist angles, demonstrating the emergence of modified electronic states arising from interlayer coupling. (**h**, **i**) Selected-area electron diffraction patterns obtained from heterobilayers with twist angles of 0.3° and 30°, confirming the crystallographic orientation used for monochromated STEM-VEELS measurements. These results demonstrate that monochromated STEM-VEELS can directly probe nanoscale variations in local electronic structure induced by moiré superlattices and interlayer coupling in two-dimensional van der Waals materials. Reproduced from Ref. (Susarla et al. 2021)

Although monochromated STEM-VEELS has been widely employed for local bandgap measurements, the same experimental framework can also probe other low-energy electronic excitations that evolve together with the band structure. A representative example is provided by twisted MoS_2_/WSe_2_ van der Waals heterobilayers, in which lattice mismatch and rotational misalignment generate a periodic moiré superlattice. Within each moiré unit cell, different local stacking configurations, such as AA and AB stacking, yield distinct interlayer coupling strengths and, consequently, different local electronic states. These spatial variations cannot be resolved by conventional ensemble-averaged optical techniques. Susarla et al. employed monochromated low-loss STEM-EELS to directly visualize these local electronic excitations within individual moiré supercells of twisted MoS_2_/WSe_2_ heterobilayers (Susarla et al. 2021). As shown in Fig. 12, atomic-resolution STEM imaging combined with spatially resolved low-loss EELS revealed distinct spectral responses associated with different stacking configurations, demonstrating that the low-energy electronic structure varies periodically across the moiré superlattice. In particular, the AA and AB stacking regions exhibit characteristic low-loss spectral features arising from their different interlayer interactions, providing direct experimental evidence of nanoscale electronic modulation within a single moiré supercell. Rather than measuring only a single average bandgap, this work illustrates that monochromated STEM-VEELS can spatially map local electronic excitations governed by interlayer coupling and atomic registry. These results significantly expand the capabilities of monochromated STEM-VEELS from quantitative bandgap determination to comprehensive mapping of local electronic structures in van der Waals materials.

**Fig. 13:**
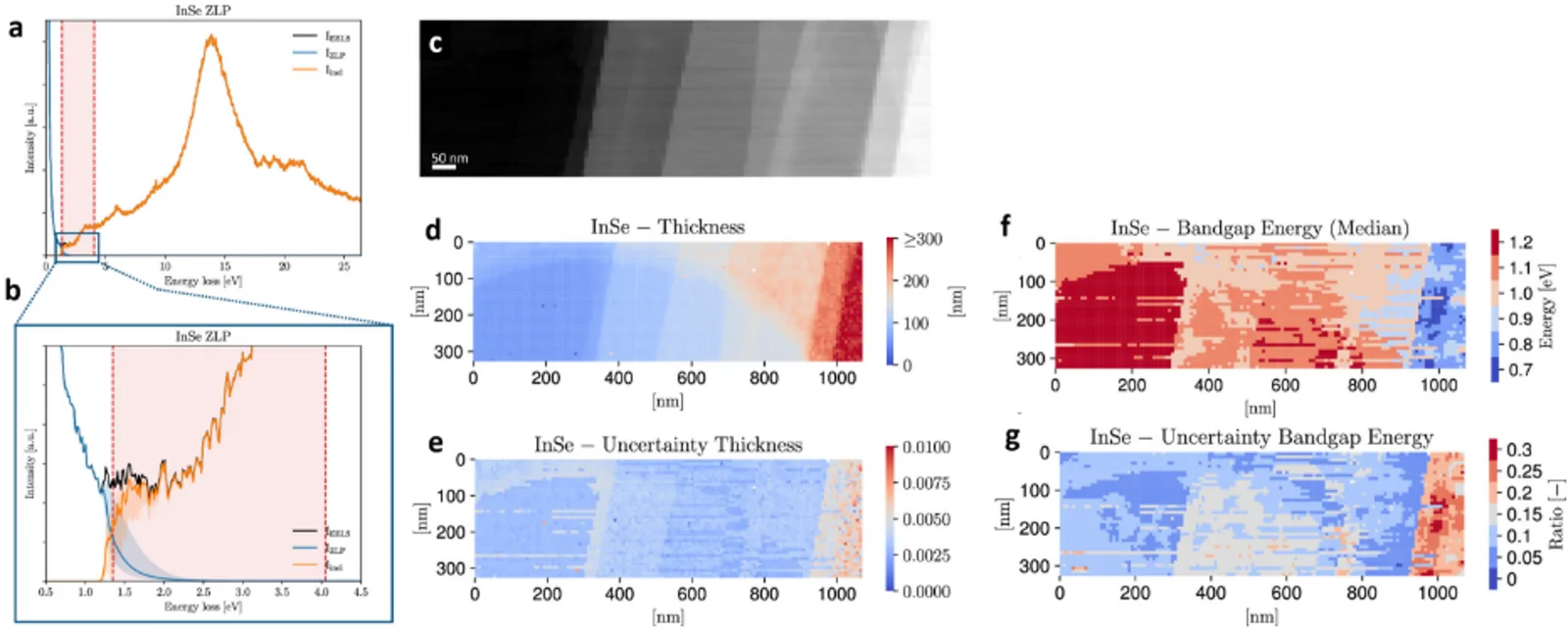
AI-assisted hyperspectral monochromated STEM-VEELS analysis of a two-dimensional InSe flake. (**a**) Representative low-loss EEL spectrum showing the zero-loss peak (ZLP), inelastic scattering signal, and the energy region used for quantitative analysis. (**b**) Enlarged view of the low-energy-loss region illustrating machine-learning-based ZLP modeling and background subtraction with uncertainty estimation using the EELSfitter framework. (**c**) STEM image of the exfoliated InSe flake used for hyperspectral spectrum imaging. (**d**, **e**) Quantitative maps of the local specimen thickness and its associated uncertainty obtained from the hyperspectral dataset. (**f**, **g**) AI-assisted mapping of the local bandgap energy and the corresponding uncertainty across the InSe flake, demonstrating automated and statistically robust extraction of spatially resolved electronic properties from monochromated STEM-VEELS spectrum imaging. Adapted from Ref. (Brokkelkamp et al. 2022)

Recent advances in artificial intelligence (AI) and machine learning (ML) have substantially expanded the analytical capability of monochromated STEM-VEELS for hyperspectral spectrum imaging. In modern STEM-EELS experiments, a complete low-loss spectrum is acquired at every probe position, generating hyperspectral datasets that may contain thousands to tens of thousands of individual spectra. Such datasets simultaneously encode rich information on local bandgap energy, dielectric response, specimen thickness, excitations, and other electronic properties. However, conventional point-by-point analysis is often labor-intensive, susceptible to user-dependent fitting bias, and impractical for large-scale quantitative characterization. To address these challenges, Brokkelkamp et al. developed an AI-assisted analysis framework for monochromated STEM-EELS by incorporating machine-learning techniques into the EELSfitter platform (Brokkelkamp et al. 2022). The workflow begins with unsupervised K-means clustering, which automatically groups individual spectra according to their local specimen thickness. These clustered spectra are subsequently used to train a deep-learning neural network that models the ZLP background without assuming a predefined analytical function. Rather than fitting each spectrum independently, the framework exploits statistical correlations among thousands of spectra in the hyperspectral dataset to achieve automated, model-independent ZLP subtraction and uncertainty estimation. The ZLP-subtracted spectra are subsequently processed using Fourier deconvolution and Kramers–Kronig analysis to determine both the local bandgap and the complex dielectric function with nanometer-scale spatial resolution. As shown in Fig. 13, the AI-assisted EELSfitter framework enables quantitative mapping of specimen thickness, local bandgap energy, and their associated uncertainties from a single monochromated STEM-EELS spectrum image. Because the machine-learning framework automatically classifies spectra with similar characteristics and performs statistically robust background modeling, it significantly reduces operator-dependent variability while improving both reproducibility and computational efficiency. Furthermore, the simultaneous visualization of thickness, bandgap, and dielectric response provides a comprehensive description of local electronic heterogeneity that would be difficult to obtain using conventional spectrum-by-spectrum analysis. These results demonstrate that AI is not merely an auxiliary post-processing tool but an integral component of next-generation monochromated STEM-VEELS, enabling high-throughput, quantitative, and uncertainty-aware characterization of low-dimensional quantum materials (Brokkelkamp et al. 2022).

Another important extension of AI- and statistics-assisted monochromated STEM-VEELS is the analysis of the energy-gain region of EELS spectra. In conventional low-loss EELS, most discussion focuses on the positive energy-loss side, where the incident electron loses energy by exciting interband transitions, plasmons, phonons, or other collective modes in the specimen. However, the EELS spectrum also contains a negative-energy-loss side, where the transmitted electron appears to gain energy through interaction with pre-existing excitations in the material. These energy-gain features can arise from thermally populated or otherwise excited quasiparticles that transfer energy to the electron beam. Although such signals are generally weak and located close to the zero-loss peak (ZLP), they can be useful because the energy-gain side is less affected by the broad inelastic scattering continuum that often obscures weak low-energy features on the conventional loss side.

**Fig. 14:**
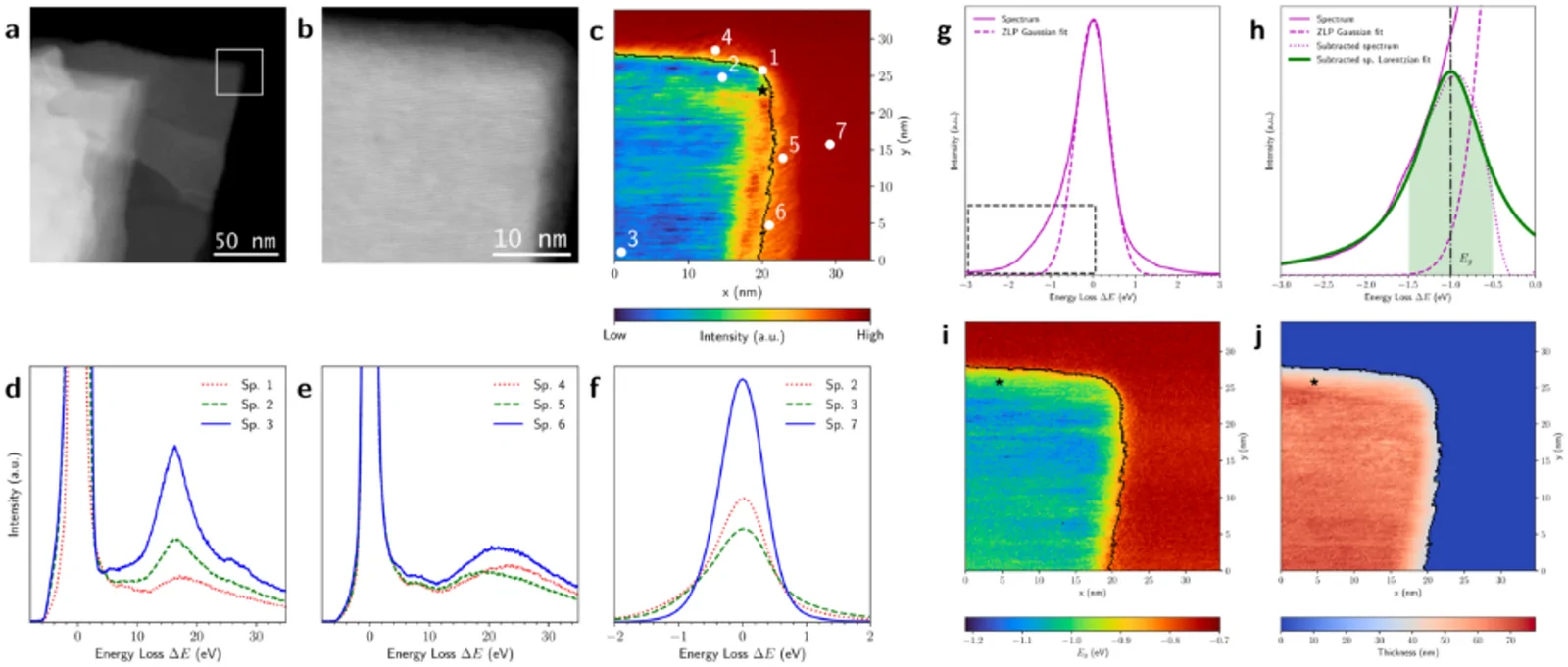
Spatially resolved energy-gain EELS analysis of a Bi_2_Te_3_ topological insulator using the EELSfitter framework. (**a**) HAADF-STEM image of an exfoliated Bi_2_Te_3_ flake. (**b**) Enlarged STEM image of the region indicated in (**a**). (**c**) STEM-EELS spectral image acquired from the selected region, with the integrated spectral intensity displayed as a color map and the specimen edge indicated by the black contour. (**d**–**f**) Representative EEL spectra collected from different positions across the vacuum, edge, and interior regions of the specimen, illustrating the evolution of bulk plasmon and low-energy gain/loss features. (**g**) Representative EEL spectrum together with the Gaussian fit of the zero-loss peak (ZLP) used for background modeling. (**h**) Enlarged view of the energy-gain region after ZLP subtraction, showing Lorentzian fitting for extraction of the characteristic energy-gain peak. (**i**) Spatial map of the extracted energy-gain peak energy obtained by pixel-by-pixel spectral fitting. (**j**) Corresponding specimen thickness map used to correlate the spatial distribution of the energy-gain peak with the local sample geometry. This workflow demonstrates automated ZLP modeling, robust peak identification, and quantitative mapping of localized low-energy excitations in quantum materials. Reproduced and adapted from Ref. (La et al. 2023)

La et al. applied spatially resolved energy-gain EELS analysis to Bi_2_Te_3_, a topological insulator in which edge- and surface-induced collective excitations are expected to appear in the low-energy region (La et al. 2023). By focusing on the energy-gain region, they identified a narrow peak around − 0.8 eV, whose intensity was strongly enhanced near specimen edges and surfaces. As shown in Fig. 14, this analysis correlates the energy-gain peak with local specimen geometry by combining STEM imaging, spectrum imaging, ZLP subtraction, Lorentzian peak fitting, and specimen thickness mapping. The result demonstrates that energy-gain analysis can provide spatially resolved information on weak collective excitations that may be difficult to isolate on the energy-loss side. A key aspect of this work is the uncertainty-aware analysis implemented within the EELSfitter framework. Because energy-gain peaks are weak and lie close to the ZLP tail, their identification depends strongly on reliable background modeling. To evaluate the robustness of the extracted peaks, La et al. applied K-means clustering to group spectra with similar local thickness, followed by Monte Carlo replica sampling to propagate ZLP-fitting uncertainty into the subtracted spectra and peak fitting results (La et al. 2023). This approach provides confidence intervals rather than only a single fitted spectrum or peak map, allowing weak energy-gain features to be assessed statistically. This example illustrates how monochromated STEM-VEELS is moving beyond conventional bandgap extraction toward automated, uncertainty-aware analysis of complex low-loss spectral images. By combining energy-gain peak analysis with EELSfitter-based spectral processing, K-means clustering, Monte Carlo uncertainty propagation, and confidence-based interpretation, weak localized excitations in quantum materials can be mapped with improved reliability and reproducibility.

Monochromated STEM-VEELS has also enabled detailed investigation of localized electronic excitations in one-dimensional (1D) nanostructures, where curvature, reduced dimensionality, and local strain produce electronic properties that differ significantly from those of planar crystals. Because these structural variations often occur over only a few nanometers, a direct correlation between atomic structure and local electronic excitations requires both high spatial and high-energy resolution. A representative example is provided by one-dimensional MoS_2_ nanotubes investigated by van der Lippe et al., who combined monochromated low-loss STEM-EELS with the EELSfitter analysis framework to quantitatively resolve excitonic, plasmonic, and bandgap-related electronic excitations from hyperspectral spectrum images (van der Lippe et al. 2023). Rather than focusing solely on the bandgap onset, their analysis simultaneously identified multiple low-energy spectral features associated with localized excitonic transitions, plasmon resonances, and interband excitations, demonstrating the rich electronic information contained within monochromated STEM-VEELS datasets.

**Fig. 15:**
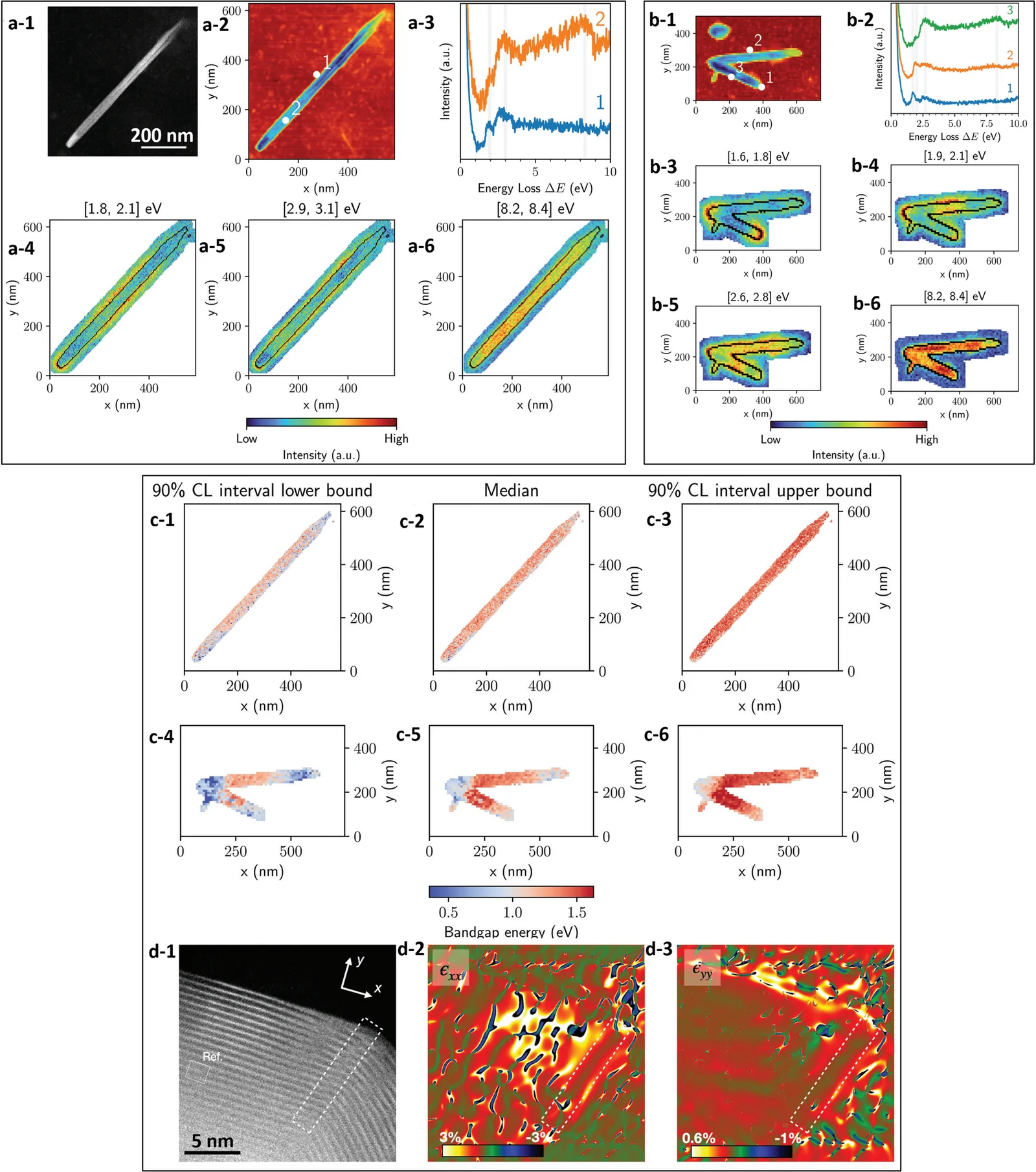
Spatially resolved monochromated STEM-VEELS analysis of one-dimensional MoS_2_ nanostructures. (**a-1** ~ **a-6**) HAADF-STEM image of an individual MoS₂ nanotube, the corresponding STEM-EELS spectrum image, representative low-loss EEL spectra acquired from different locations, and spatial intensity maps integrated over selected energy windows, revealing the localization of distinct low-energy electronic excitations and plasmonic features. (**b-1** ~ **b-6**) Equivalent analysis performed on two interconnected MoS₂ nanotubes, demonstrating that the spatial distribution of electronic excitations varies with local geometry and structural connectivity. (**c-1** ~ **c-6**) Spatially resolved bandgap maps showing the 90% confidence interval lower bound, median, and upper bound obtained from EELSfitter analysis for both individual and interconnected nanotubes, illustrating quantitative mapping of local bandgap variations together with their statistical uncertainty. (**d-1** ~ **d-3**) Atomic-resolution HAADF-STEM image and corresponding geometrical phase analysis (GPA) strain maps (ε_xx_ and ε_yy_), revealing localized tensile strain associated with structural defects near the bent region of the nanotube. The correlation between strain mapping and bandgap distribution demonstrates that local curvature and defect-induced strain modulate the electronic structure of one-dimensional MoS_2_ nanostructures. Reproduced and adapted from Ref. (van der Lippe et al. 2023)

As shown in Fig. 15, spatially resolved spectral analysis revealed that the excitonic transitions near 2 ~ 3 eV exhibit pronounced spatial localization, whereas the plasmon resonance near 8.3 eV remains more uniformly distributed throughout the nanotube. Quantitative bandgap mapping further demonstrated that the central region of the nanotube preserves an indirect bandgap close to the bulk MoS₂ value, while the highly curved end regions exhibit a measurable bandgap reduction. Correlation with atomic-resolution STEM imaging and geometrical phase analysis confirmed that these local electronic variations originate from curvature-induced tensile strain and structural defects, which modify both the electronic band structure and the localization of excitonic states. This study demonstrates that monochromated STEM-VEELS can simultaneously map local bandgap energy, excitonic excitations, plasmon resonances, and strain-dependent electronic modulation within a single nanostructure. Such multidimensional characterization considerably expands the capability of monochromated STEM-VEELS in addition to conventional local bandgap measurements, providing direct insight into the interplay between atomic structure and electronic properties in low-dimensional quantum materials.

### Representative applications of monochromated STEM-VEELS

The examples presented throughout this section demonstrate the rapid evolution of monochromated STEM-VEELS from a technique primarily developed for nanoscale bandgap measurements to a comprehensive platform for quantitative electronic-structure characterization. Advances in monochromator technology, spectrum-imaging acquisition, detector performance, and computational analysis have considerably expanded the range of accessible materials and physical phenomena. Table 9 summarizes representative applications of monochromated STEM-VEELS reported to date, including the investigated materials, measurement targets, characteristic excitation energies, and principal findings. The technique has been successfully applied to a broad spectrum of materials, ranging from narrow-bandgap semiconductors such as PbS to wide-bandgap dielectrics including SiO_2_ and diamond, as well as semiconductor heterostructures, oxide interfaces, quantum-confined nanostructures, van der Waals materials, topological materials, and one-dimensional nanostructures. The measurable energy range spans from approximately 0.4 eV for PbS thin films to more than 9 eV for SiO_2_, highlighting the versatility of monochromated STEM-VEELS across an exceptionally wide electronic energy window. Other than quantitative bandgap determination, recent studies have demonstrated that monochromated STEM-VEELS can probe a wide variety of localized electronic excitations, including surface plasmons, excitons, moiré-induced electronic states, interlayer coupling, strain-dependent electronic modulation, and energy-gain features. Furthermore, the integration of AI-assisted hyperspectral analysis, automated spectral fitting, Bayesian inference, and uncertainty quantification has significantly improved the robustness and reproducibility of quantitative electronic structure analysis. Collectively, these advances establish monochromated STEM-VEELS as a versatile platform for nanoscale characterization of local electronic structures and low-energy excitations across diverse classes of functional materials.

**Table 9:** Representative materials investigated by monochromated STEM-VEELS

Material	Measurement target	Technique	Characteristic energy (eV)	Representative findings	Reference
PbS quantum dots	Local bandgap	80 kV monochromated STEM-VEELS	0.7–1.5	Size- and position-dependent quantum-confined bandgap mapping under Cherenkov-free conditions	Jung et al. 2013
PbS thin film (~ 20 nm thick)	Local bandgap	80 kV monochromated STEM-VEELS	~ 0.41	Quantitative narrow-bandgap measurement under Cherenkov-free conditions	Jung et al. 2013
SiO2	Local bandgap	80 kV monochromated STEM-VEELS	~ 9.0	Wide-bandgap dielectric measured without Cherenkov radiation	Jung et al. 2013
GaN	Local bandgap / dielectric function	STEM-EELS	~ 3.4	Nanoscale dielectric-function and bandgap analysis in GaN and related heterostructures	Brockt and Lakner 2000
HfO2 thin film	Local bandgap / dielectric function	VEELS	~ 5.7	Bandgap and dielectric-function determination in nanoscale oxide thin films	Cheynet et al. 2007
a-SiN_x_ (N/Si = 1.46)	Local bandgap	Monochromated STEM-EELS	5.3	Composition-dependent nanoscale bandgap mapping	Park et al. 2009
a-SiN_x_ (N/Si = 1.20)	Local bandgap	Monochromated STEM-EELS	4.1	Bandgap reduction with decreasing nitrogen content	Park et al. 2009
a-SiN_x_ (N/Si = 0.92)	Local bandgap	Monochromated STEM-EELS	2.9	Composition-sensitive bandgap variation in amorphous silicon nitride	Park et al. 2009
SiO_2_	Local bandgap	Monochromated STEM-EELS	8.95	Benchmark wide-bandgap dielectric measurement	Park et al. 2009
Diamond	Local bandgap	Monochromated STEM-EELS	6.14 ± 0.23	Wide-bandgap benchmark material for onset determination	Park et al. 2009
Diamond	Dielectric function	VEELS	Indirect gap region	Retrieval of dielectric function from valence-loss EELS	Zhang et al. 2008
Diamond	Indirect bandgap	Off-axis STEM-VEELS	~ 5.6	Indirect bandgap measurement with suppressed Cherenkov contribution	Korneychuk et al. 2018a, b
GaN	Local bandgap	Off-axis Bessel STEM-VEELS	3.3	Layer-resolved bandgap mapping in nitride multilayers	Korneychuk et al. 2018a, b
AlGaN	Local bandgap	Off-axis Bessel STEM-VEELS	5.0	Local bandgap determination of AlGaN barrier layers	Korneychuk et al. 2018a, b
AlN	Local bandgap	Off-axis Bessel STEM-VEELS	5.6	Wide-bandgap AlN resolved using momentum-filtered acquisition	Korneychuk et al. 2018a, b
β-(Al_0.2_Ga_0.8_)_2_O_3_	Local bandgap	STEM-VEELS	5.04	Bandgap of epitaxial β-(Al, Ga)2O3 film	Chmielewski et al. 2022
β-Ga_2_O_3_	Local bandgap	STEM-VEELS	4.79	Local bandgap of β-Ga2O3 substrate	Chmielewski et al. 2022
β-(Al_0.2_Ga_0.8_)_2_O_3_/β-Ga_2_O_3_ interface	Interface bandgap	STEM-VEELS	~ 4.57	Defect-induced bandgap narrowing at the heterointerface	Chmielewski et al. 2022
ZnCdO	Local bandgap	STEM-VEELS	2.94 ± 0.21	Alloy-semiconductor bandgap mapping	Granerød et al. 2018
ZnO	Local bandgap	STEM-VEELS	3.22 ± 0.01	Reference wide-bandgap semiconductor measurement	Granerød et al. 2018
Ga-doped ZnO	Conduction-band plasmon / Burstein–Moss shift	STEM-EELS	Plasmon-related low-loss features	Direct observation of conduction-band plasmons and doping-induced Burstein–Moss shift	Granerød et al. 2018
GeTe (crystalline)	Local bandgap	Monochromated STEM-EELS	0.6	Crystalline phase with reduced bandgap	Oh et al. 2023
GeTe (amorphous)	Local bandgap	Monochromated STEM-EELS	0.9	Amorphous phase showing increased bandgap	Oh et al. 2023
Thin-film solar cell materials	Local bandgap	VEELS	Spatially varying	Local bandgap variation in thin-film photovoltaic materials	Keller et al. 2014
CsPbBr_3_ nanocrystals	Band structure modification	STEM-VEELS	~ 2.3–2.4	Direct observation of quantum-confinement-induced band structure modification	Lin et al. 2016
CsPbBr_3_ nanosheets	Local bandgap	STEM-EELS	2.44–2.49	Thickness-dependent bandgap in individual orthorhombic nanosheets	Brescia et al. 2020
InSe	Local bandgap	Monochromated STEM-VEELS + AI	0.9–1.3	AI-assisted hyperspectral bandgap and dielectric-function mapping	Brokkelkamp et al. 2022
WS_2_ nanoflowers	Local bandgap	Monochromated STEM-VEELS + AI	1.6 ± 0.3	AI-assisted bandgap extraction from low-loss spectral images	Brokkelkamp et al. 2022
MoS_2_/WSe_2_ heterobilayer	Local electronic excitations	Monochromated STEM-VEELS	1.62 (AA), 1.80 (AB)	Moiré-stacking-dependent local electronic structure	Susarla et al. 2021
Ag nanoparticles	Surface plasmon resonance	STEM-EELS	~ 3–4	Bright and dark plasmon mode mapping in individual nanoparticles and dimers	Koh et al. 2009
Au nanoparticles	Quantum plasmon resonance	STEM-EELS	Size dependent	Quantum plasmon confinement in individual metallic nanoparticles	Scholl et al. 2012
Bi_2_Te_3_	Energy-gain peak	STEM-EELS + Bayesian inference	~ 0.8–1.1	Edge-induced energy-gain excitations analyzed with uncertainty-aware spectral fitting	La et al. 2023
1D MoS_2_ nanostructures	Bandgap, excitons and plasmons	Monochromated STEM-VEELS + AI	Eg ≈ 1.2–1.6; excitations at ~ 2, ~3 and ~ 8.3	Correlative mapping of strain, excitonic response, plasmonic features and bandgap modulation	van der Lippe et al. 2023
GaN	Local bandgap	Monochromated STEM-VEELS	~ 3.5	High-energy-resolution monochromated benchmark measurement	Addiego et al. 2024
SiO_2_	Local bandgap	Monochromated STEM-VEELS	~ 9.1	High-energy-resolution benchmark measurement of a wide-bandgap dielectric	Addiego et al. 2024

## Future perspectives

Monochromated STEM-VEELS has evolved from a specialized electron spectroscopic technique into a powerful platform for quantitatively probing local electronic structures with nanometer-scale spatial resolution and millielectronvolt-scale energy resolution. As discussed throughout this review, recent advances in electron monochromation, aberration-corrected optics, optimized spectral analysis, and artificial intelligence have significantly expanded its capabilities beyond conventional bandgap measurements to encompass dielectric responses, excitonic transitions, plasmonic excitations, and other low-energy electronic phenomena in a broad range of material systems.

Despite these remarkable achievements, several technical challenges remain before monochromated STEM-VEELS can become a universally applicable and quantitative metrology tool. Electron-beam-induced damage continues to limit investigations of highly sensitive materials, while the requirement for electron-transparent specimens restricts the analysis of bulk samples. Furthermore, the rapidly increasing volume and complexity of hyperspectral datasets demand automated, statistically robust, and user-independent analytical workflows. Addressing these challenges will require continued advances in instrumentation, experimental methodologies, and computational data analysis.

Looking ahead, future developments are expected to proceed along several complementary directions. Cryogenic electron spectroscopy will enable reliable characterization of beam-sensitive materials, while ultrafast and operando monochromated STEM-VEELS will provide direct access to dynamic electronic processes in response to external stimuli. Artificial intelligence is anticipated to transform hyperspectral data processing into autonomous, uncertainty-aware electronic-structure analysis. In parallel, emerging monochromated SEM-based reflection electron energy-loss spectroscopy (SEM-REELS) offers a promising nondestructive alternative for probing surface electronic structures in bulk specimens without the need for electron-transparent sample preparation. Collectively, these advances will move monochromated electron energy-loss spectroscopy beyond a high-resolution characterization technique toward a comprehensive, intelligent, and quantitative metrology platform capable of probing local electronic structures across increasingly complex materials systems.

### Cryogenic monochromated STEM-VEELS for beam-sensitive materials

Despite the remarkable progress achieved with monochromated STEM-VEELS under ambient conditions, electron-beam-induced damage remains a major limitation to quantitative measurements of local electronic structures in beam-sensitive materials. Organic–inorganic hybrid perovskites, organic semiconductors, polymers, metal–organic frameworks, and many two-dimensional van der Waals materials readily undergo radiolysis, knock-on damage, and mass loss during electron irradiation, resulting in irreversible modifications of their electronic structures and consequently reducing the reliability of local bandgap measurements (Egerton 2011, 2017).

Cryogenic electron microscopy provides an effective strategy to mitigate these beam-induced artifacts by significantly suppressing atomic diffusion, radiolytic decomposition, and thermally activated structural degradation. Operating at cryogenic temperatures also reduces phonon populations and thermal diffuse scattering, narrowing spectral broadening in the low-loss region and improving the precision of zero-loss peak subtraction and bandgap onset determination. As monochromated STEM-VEELS continues to approach energy resolutions of only a few millielectronvolts, reducing thermal broadening will become increasingly important for resolving subtle interband transitions, excitonic features, and other low-energy electronic excitations that are difficult to distinguish at room temperature.

Cryogenic monochromated STEM-VEELS is therefore expected to play an increasingly important role in the quantitative characterization of fragile quantum materials, including hybrid perovskites, low-dimensional semiconductors, organic optoelectronic materials, and emerging van der Waals heterostructures. Nevertheless, careful interpretation remains necessary because intrinsic electronic structures themselves evolve with temperature through electron–phonon interactions and lattice expansion. Future developments will therefore require quantitative correction schemes that combine cryogenic experiments with first-principles calculations to accurately separate intrinsic temperature-dependent bandgap shifts from beam-induced artifacts. Such developments will further establish cryogenic monochromated STEM-VEELS as a reliable platform for quantitative nanoscale electronic-structure metrology in highly beam-sensitive materials.

### Ultrafast and operando monochromated STEM-VEELS

Although monochromated STEM-VEELS has been primarily employed to characterize static electronic structures, many technologically important materials operate under dynamic nonequilibrium conditions in which their local electronic properties continuously evolve over time and in response to external stimuli. Future developments are therefore expected to extend monochromated STEM-VEELS against static bandgap mapping toward real-time observation of transient electronic structures during device operation. Such capabilities would provide direct insight into carrier relaxation, charge transfer, phase transformations, and other ultrafast processes that govern the performance of functional materials.

One promising direction is the integration of monochromated electron spectroscopy with ultrafast electron microscopy (UEM). By employing femtosecond or picosecond pump–probe schemes, time-resolved electron energy-loss spectroscopy enables direct visualization of nonequilibrium electronic excitations with simultaneous high spatial and temporal resolution. Early demonstrations have shown that ultrafast EELS can monitor transient plasmonic responses and photoinduced electronic dynamics following optical excitation, enabling investigations of carrier relaxation pathways, exciton formation, and ultrafast energy transfer processes in nanoscale materials (Piazza et al. 2013; Cremons et al. 2016). As monochromator technology and pulsed electron sources continue to advance, the combination of ultrahigh energy resolution with femtosecond temporal resolution is expected to provide unprecedented insight into dynamic low-energy electronic excitations.

Along with ultrafast measurements, increasing attention is also being directed toward operando monochromated STEM-VEELS, in which local electronic structures are monitored while external stimuli such as electrical bias, temperature, optical illumination, or reactive gas environments are continuously applied. Unlike conventional ex situ characterization, operando experiments allow direct observation of how local band structures evolve during actual device operation. Such approaches will be particularly valuable for investigating resistive switching in memristors, carrier transport across semiconductor heterostructures, electrochemical reactions in batteries, photocatalytic processes, and phase transitions in quantum materials. Coupling monochromated STEM-VEELS with in situ biasing and environmental transmission electron microscopy, therefore, represents an important step toward quantitative characterization of functional electronic materials under realistic operating conditions.

Future instrumentation is expected to integrate monochromated electron sources, ultrafast pulsed beams, advanced in situ holders, and high-speed direct electron detectors into unified experimental platforms capable of simultaneously achieving high spatial, temporal, and energy resolution. Together with increasingly sophisticated computational analysis, these developments will transform monochromated STEM-VEELS from a static characterization technique into a dynamic spectroscopic platform capable of quantitatively tracking the evolution of local bandgaps, dielectric responses, excitonic transitions, and plasmonic excitations in real time.

### Artificial intelligence for hyperspectral EELS analysis and future autonomous spectroscopy

Artificial intelligence (AI) and machine learning (ML) are expected to play an increasingly important role in transforming monochromated STEM-VEELS from a user-dependent characterization technique toward a more automated spectroscopy platform capable of extracting quantitative electronic-structure information from increasingly large and complex hyperspectral datasets. Modern monochromated spectrum imaging routinely generates tens of thousands of spectra within a single experiment, making conventional workflows for zero-loss peak subtraction, background modeling, bandgap onset determination, and spectral interpretation increasingly time-consuming and susceptible to user-dependent variability. Consequently, AI-assisted data analysis is emerging as one of the most important developments for achieving high-throughput, reproducible, and quantitative nanoscale electronic-structure characterization. However, it is important to distinguish between AI approaches already demonstrated for low-loss STEM-EELS/VEELS and bandgap analysis and those developed primarily for broader EELS, STEM imaging, or other microscopy applications that may be transferable to monochromated STEM-VEELS.

Among the approaches directly demonstrated for low-loss EELS and local electronic-structure analysis, machine-learning-assisted ZLP modeling represents one of the most established applications. EELSfitter employs artificial neural networks together with Monte Carlo replica sampling for model-independent ZLP estimation and uncertainty propagation, enabling robust analysis of weak low-energy spectral features (Roest et al. 2021). This framework was subsequently extended to hyperspectral EELS datasets, where automated ZLP subtraction enabled spatially resolved determination of local bandgap, specimen thickness, and complex dielectric function in two-dimensional materials (Brokkelkamp et al. 2022). These studies demonstrate that ML can already contribute directly to quantitative VEELS analysis by reducing user-dependent background modeling and enabling reproducible processing of large spectrum-image datasets.

Beyond direct bandgap analysis, AI and deep-learning approaches have also been demonstrated in broader EELS applications and provide methodologies that may be transferable to monochromated VEELS. Convolutional neural network (CNN)-based methods have been applied to denoising and classification of rapidly acquired EELS datasets (Pate et al. 2021), while deep-learning approaches have enabled automated identification and characterization of core-loss EELS features (Annys et al. 2023). Machine-learning approaches have also been used to identify spatial features associated with low-loss plasmonic and core-loss excitations in multidimensional EELS datasets (Roccapriore et al. 2024). Although these approaches have not necessarily been demonstrated for quantitative bandgap extraction, their ability to denoise, classify, and identify subtle spectral variations suggests potential applicability to monochromated STEM-VEELS datasets.

Complementing supervised learning, unsupervised deep-learning models are becoming increasingly important for exploring large, multidimensional datasets without requiring manually labeled training data. Variational autoencoders (VAEs) compress high-dimensional spectral datasets into low-dimensional latent representations while preserving their essential physical characteristics, enabling efficient visualization and interpretation of complex hyperspectral data. Building upon this concept, convolutional variational autoencoders (CVAEs) combine convolutional feature extraction with latent-space representation learning, enabling the automatic identification of subtle spatial and spectroscopic variations. Three-dimensional convolutional variational autoencoders (3D-CVAEs) have demonstrated powerful capabilities for anomaly detection in multidimensional EELS datasets by identifying unusual spectral features that may be overlooked using conventional analysis (Sultanov et al. 2025). These approaches have been further extended by integrating principal component analysis (PCA), silhouette-score optimization, and k-means clustering. PCA reduces the dimensionality of hyperspectral datasets while preserving their dominant spectral variations, whereas k-means clustering automatically groups spectra with similar characteristics without requiring prior knowledge of the underlying classes. Such integrated unsupervised workflows can enable statistically robust identification of structural and spectroscopic anomalies while minimizing user bias (Ayyubi et al. 2026). Although these unsupervised approaches have been demonstrated across STEM imaging and EELS applications, their application specifically to quantitative monochromated VEELS bandgap analysis remains relatively limited. Nevertheless, their capabilities for dimensionality reduction, clustering, and anomaly detection suggest potential applicability to the automated identification of local bandgap variations, plasmonic excitations, excitonic features, and other low-energy electronic excitations.

More general AI frameworks developed for electron and scanning probe microscopy may provide additional tools for future VEELS analysis. For example, the AtomAI framework integrates convolutional neural networks, variational autoencoders, deep kernel learning, Bayesian optimization, and atomistic feature analysis within a common framework for image and spectroscopic data analysis (Ziatdinov et al. 2022). Although such integrated approaches have not yet been established as autonomous monochromated STEM-VEELS workflows, they provide methodological components that could potentially be adapted for automated feature extraction and structure–property correlation in hyperspectral VEELS datasets. Graph neural networks (GNNs) may offer another future direction by representing atoms as graph nodes and their local relationships as edges, potentially enabling correlations between atomic configurations, strain, defects, and spatially resolved electronic properties. Such applications to quantitative VEELS remain prospective and will require dedicated development and experimental validation.

Alongside advances in deep learning, uncertainty-aware data analysis is becoming an increasingly important component of quantitative electron spectroscopy. Bayesian inference estimates probability distributions rather than single deterministic values, allowing measurement uncertainties to be explicitly quantified. Monte Carlo sampling further propagates experimental uncertainties by repeatedly perturbing spectral fitting parameters according to the underlying noise characteristics, generating statistically robust confidence intervals for extracted electronic parameters. Instead of reporting only a single bandgap value, future monochromated STEM-VEELS studies may increasingly incorporate confidence-aware electronic-structure measurements that account for uncertainties arising from spectral noise, zero-loss peak subtraction, background modeling, and fitting procedures. Such probabilistic approaches could improve reproducibility, facilitate quantitative comparison between different laboratories, and enhance the reliability of nanoscale electronic-structure metrology.

Looking further ahead, AI-assisted analysis may become increasingly integrated with automated and adaptive electron microscopy workflows. Approaches being explored in the broader electron-microscopy community, including real-time data analysis, adaptive acquisition, AI-guided feature identification, and closed-loop experimental control, could eventually be adapted to monochromated STEM-VEELS. Such developments may enable automated identification of regions of interest, optimization of acquisition parameters, and real-time assessment of low-loss spectral features. However, fully autonomous quantitative STEM-VEELS, particularly for local bandgap metrology, has not yet been established and will require further advances in robust spectral analysis, uncertainty quantification, experimental control, and cross-instrument validation. Continued progress in these areas could ultimately support more automated, reproducible, and high-throughput nanoscale electronic-structure measurements.

### Emerging monochromated SEM-REELS for nondestructive surface electronic-structure characterization

Although monochromated STEM-VEELS has been one of the most powerful techniques for nanoscale electronic-structure characterization, its application remains fundamentally dependent on electron-transparent specimens prepared by focused ion beam (FIB) milling, ion polishing, or mechanical thinning. Such specimen preparation is often destructive, time-consuming, and unsuitable for many technologically important bulk materials, large-area wafers, and industrial devices. In addition, high-energy electron irradiation may induce structural modification or beam damage in beam-sensitive materials. These practical limitations have motivated the development of complementary electron spectroscopic techniques that can probe electronic structures directly from bulk specimens without destructive sample preparation.

Reflection electron energy-loss spectroscopy (REELS), implemented in a scanning electron microscope (SEM), has recently emerged as a promising surface-sensitive alternative for high-energy-resolution electronic-structure characterization. Unlike transmission EELS, REELS analyzes low-energy electrons reflected from the specimen surface, enabling direct characterization of bulk materials without preparing electron-transparent TEM specimens. Owing to the use of primary electron energies typically below several kiloelectronvolts, REELS is inherently free from Cherenkov radiation and provides high sensitivity to near-surface electronic excitations, including bandgap transitions, interband excitations, plasmons, and dielectric responses.

**Fig. 16:**
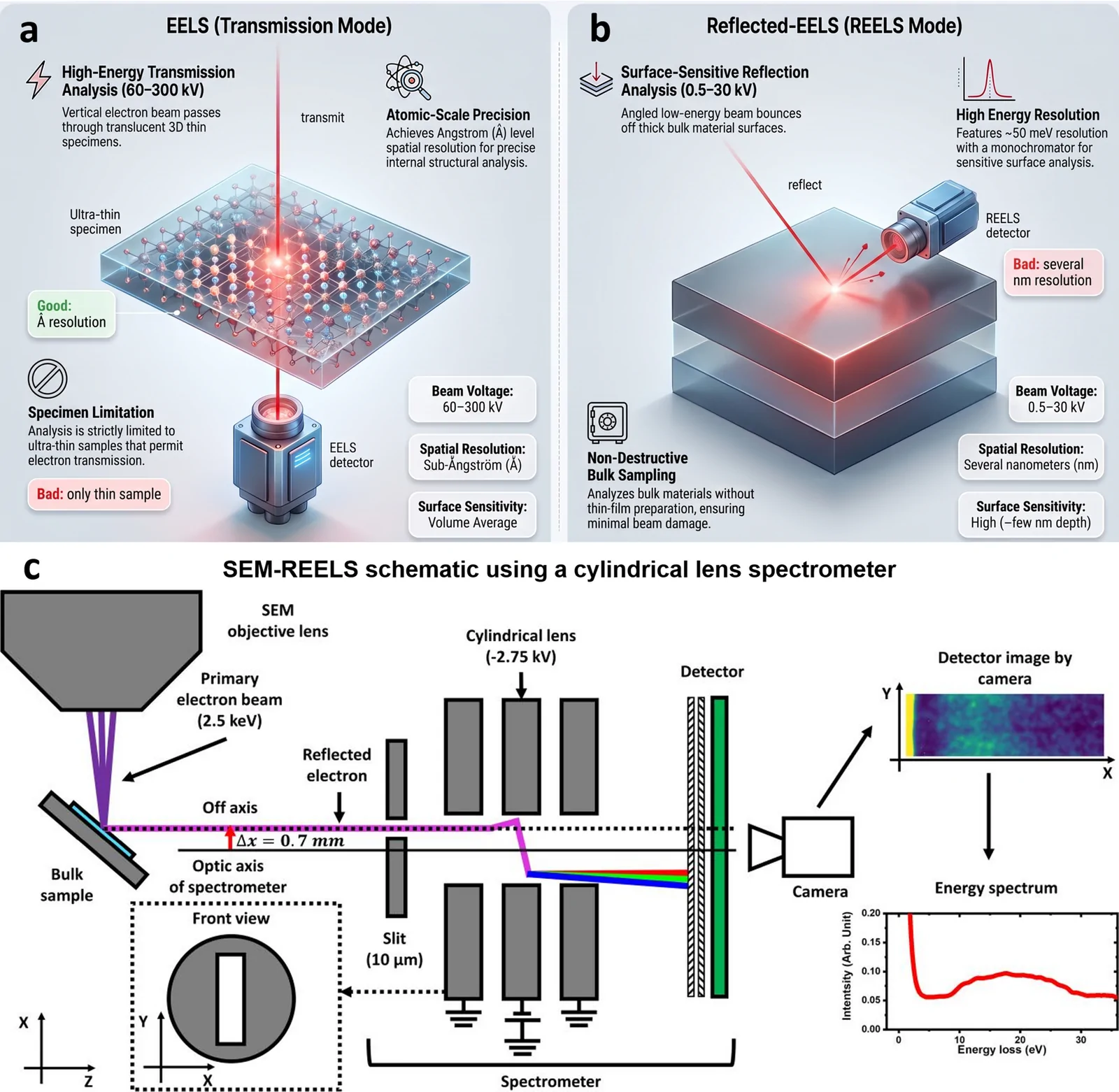
Comparison of transmission EELS and reflection EELS (REELS). **a** Schematic illustration of conventional transmission EELS in STEM. **b** Schematic illustration of reflection EELS (REELS) in SEM. **c** Schematic illustration of a monochromated SEM-REELS system employing a cylindrical lens spectrometer with parallel detection. The reflected electrons generated by the primary electron beam are collected through an off-axis slit, dispersed by energy in the cylindrical lens spectrometer, and simultaneously detected by a parallel detector consisting of double microchannel plates (MCPs) and a phosphor screen. The detector image is converted into an energy-loss spectrum through image processing and energy calibration. Panel (**c**) is adapted from Ref. (Hwang et al. 2025)

**Fig. 17:**
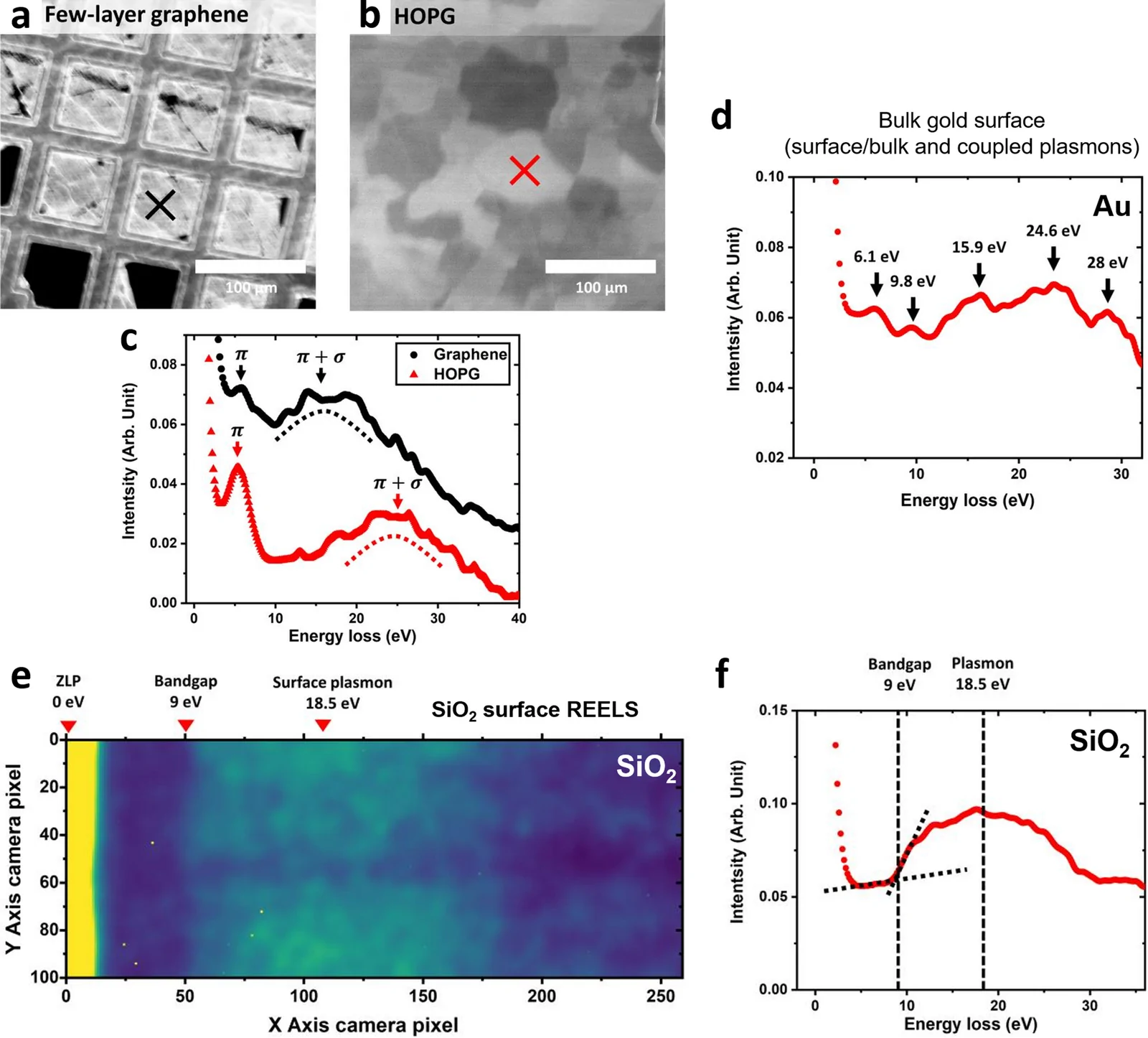
Representative monochromated SEM-REELS results for surface electronic-structure characterization. **a**, **b** SEM images of few-layer graphene and highly oriented pyrolytic graphite (HOPG), with the electron-beam positions indicated by crosses. **c** Reflection electron energy-loss spectra of graphene and HOPG, showing distinct π and π + σ plasmon excitations. **d** Reflection electron energy-loss spectrum of a bulk Au surface, revealing multiple energy-loss features associated with surface plasmons, bulk plasmons, and their coupled modes. **e** Representative detector image acquired from a SiO_2_ surface using the parallel-detection SEM-REELS system. **f** Corresponding REELS spectrum of SiO_2_, showing the bandgap onset at approximately 9 eV and the surface plasmon peak near 18.5 eV without observable Cherenkov contributions. Adapted from Ref. (Hwang et al. 2025)

A significant advance is the development of a monochromated SEM-REELS system employing a cylindrical lens spectrometer with parallel detection (Hwang et al. 2025). Figure 16 compares the fundamental operating principles of conventional transmission EELS and reflection EELS (REELS), along with the schematic configuration of the recently developed monochromated SEM-REELS system employing a cylindrical-lens spectrometer with parallel detection. In this instrument, reflected electrons generated by a monochromated primary electron beam are energy-dispersed using a cylindrical lens spectrometer and simultaneously detected by a parallel detector consisting of double microchannel plates (MCPs) and a phosphor screen. The parallel-detection configuration substantially improves acquisition efficiency while maintaining an energy resolution of approximately 50 meV, enabling reliable characterization of low-energy electronic excitations from bulk specimens. The system’s performance was demonstrated using representative materials, including graphene, highly oriented pyrolytic graphite (HOPG), Au, and SiO_2_, as summarized in Fig. 17. For graphene and HOPG, the characteristic π and π + σ plasmon excitations were clearly resolved, demonstrating the capability of SEM-REELS to distinguish subtle differences in the electronic structures of sp^2^-bonded carbon materials. Measurements on bulk Au further revealed multiple energy-loss peaks at 6.1, 9.8, 15.9, 24.6, and 28 eV, corresponding to surface plasmons (ES), bulk plasmons (EB), and higher-order coupled plasmon excitations (e.g., ES + EB and ES + 2EB). The clear separation of these plasmon modes demonstrates the high energy resolution and sensitivity of monochromated SEM-REELS for probing collective electronic excitations at metallic surfaces. Furthermore, the measured plasmon energies are in good agreement with previous theoretical and experimental studies, confirming the technique’s reliability for quantitative plasmon spectroscopy. Measurements on SiO_2_ successfully identified the bandgap onset near 9 eV and the surface plasmon at approximately 18.5 eV, with no observable Cherenkov contributions, highlighting the applicability of monochromated SEM-REELS for quantitative bandgap measurements in wide-bandgap dielectric materials.

Although the current spatial resolution of SEM-REELS remains limited to several nanometers, continued advances in monochromators, electron optics, spectrometer design, and detector technology are expected to further improve both energy and spatial resolution. Rather than replacing monochromated STEM-VEELS, SEM-REELS should be regarded as a complementary spectroscopy platform that extends quantitative electronic-structure characterization from electron-transparent TEM specimens to bulk materials and device surfaces. Combined with recent developments in AI-assisted spectral analysis, uncertainty-aware bandgap estimation, and automated hyperspectral mapping, monochromated SEM-REELS has the potential to become a versatile, nondestructive platform for quantitative surface electronic-structure metrology in semiconductors, catalysts, energy materials, and industrial wafer inspection.

## Conclusions

Monochromated STEM-VEELS has become one of the most powerful electron spectroscopic techniques for probing local electronic structures with nanometer-scale spatial resolution and millielectronvolt-scale energy resolution. By enabling direct measurements of bandgaps, dielectric functions, interband transitions, excitonic features, plasmons, and other low-energy electronic excitations, monochromated STEM-VEELS has significantly expanded the capabilities of transmission electron microscopy beyond conventional structural and chemical characterization. Throughout this review, we have discussed the fundamental principles of low-loss EELS, recent advances in monochromator technology and experimental methodologies, strategies for quantitative bandgap determination, and representative applications across a wide range of semiconductor, dielectric, low-dimensional, and quantum materials.

Recent developments have substantially broadened the scope of monochromated STEM-VEELS from qualitative spectroscopy toward quantitative electronic-structure metrology. Improvements in energy resolution, optimized spectral processing, off-axis acquisition geometries, and advanced analytical approaches have enabled increasingly reliable measurements of local bandgap variations while minimizing the influence of the zero-loss peak and Cherenkov radiation. Furthermore, recent applications to two-dimensional materials, one-dimensional nanostructures, moiré heterostructures, phase-change materials, and topological materials demonstrate that monochromated STEM-VEELS can provide direct insight into complex electronic phenomena at the nanoscale. As summarized in this review, the range of measurable electronic properties has also expanded beyond bandgap determination to include excitonic transitions, plasmonic excitations, dielectric responses, and energy-gain features, highlighting the versatility of monochromated low-loss electron spectroscopy.

Equally important is the rapid emergence of computational electron spectroscopy. Artificial intelligence and machine learning are transforming hyperspectral EELS analysis by enabling automated denoising, spectral segmentation, feature recognition, and quantitative extraction of electronic properties from increasingly large multidimensional datasets. Recent developments based on convolutional neural networks, variational autoencoders, convolutional variational autoencoders, and integrated AI frameworks are expected to accelerate autonomous hyperspectral analysis while reducing user-dependent variability. At the same time, Bayesian inference and Monte Carlo sampling are introducing statistically rigorous uncertainty quantification into bandgap measurements, allowing confidence intervals and probabilistic electronic-structure analysis to become an integral part of future quantitative spectroscopy. These developments represent an important transition from deterministic spectral interpretation toward uncertainty-aware and data-driven electronic-structure metrology.

Future advances are expected to arise from the synergistic integration of next-generation instrumentation and intelligent data analysis. Cryogenic and operando monochromated STEM-VEELS will enable quantitative characterization of beam-sensitive materials and dynamic electronic processes under realistic operating conditions, while ultrafast electron spectroscopy will provide direct access to transient carrier dynamics and nonequilibrium electronic states. In parallel, the recent development of monochromated SEM-based reflection electron energy-loss spectroscopy (SEM-REELS) provides a promising complementary platform for characterizing nondestructive surface electronic structure of bulk materials without electron-transparent specimen preparation. Together with AI-assisted hyperspectral analysis and uncertainty-aware workflows, these emerging techniques will significantly broaden the applicability of electron energy-loss spectroscopy across both fundamental research and industrial metrology.

In conclusion, monochromated electron energy-loss spectroscopy is rapidly evolving beyond a high-resolution characterization technique into a comprehensive platform for quantitative electronic-structure metrology. Continued advances in monochromated instrumentation, computational spectroscopy, artificial intelligence, uncertainty quantification, and complementary electron spectroscopic techniques will further enhance its capability to probe local electronic structures across multiple length scales, from atomic interfaces to bulk device surfaces. These developments are expected to play an increasingly important role in the design, optimization, and quality assessment of next-generation semiconductor, quantum, energy, and functional materials, establishing monochromated electron energy-loss spectroscopy as a cornerstone technique for future nanoscale electronic-structure characterization.

## Data Availability

No datasets were generated or analyzed during the current study. Any materials related to this review are available from the corresponding author upon reasonable request.

## Abbreviations

AI: Artificial intelligence
CNN: Convolutional neural network
CL: Cathodoluminescence
CVAE: Convolutional variational autoencoder
EB: Bulk plasmon energy
EELS: Electron energy-loss spectroscopy
ES: Surface plasmon energy
FIB: Focused ion beam
FWHM: Full width at half maximum
GNN: Graph neural network
HAADF: High-angle annular dark-field
HOPG: Highly oriented pyrolytic graphite
JDOS: Joint density of states
KKA: Kramers–Kronig analysis
LDOS: Local density of states
MCP: Microchannel plate
ML: Machine learning
PL: Photoluminescence
QD: Quantum dot
REELS: Reflection electron energy-loss spectroscopy
SEM: Scanning electron microscopy
SEM-REELS: Scanning electron microscopy–reflection electron energy-loss spectroscopy
SNR: Signal-to-noise ratio
STEM: Scanning transmission electron microscopy
STEM-EELS: Scanning transmission electron microscopy–electron energy-loss spectroscopy
STEM-VEELS: Scanning transmission electron microscopy–valence electron energy-loss spectroscopy
STS: Scanning tunneling spectroscopy
TEM: Transmission electron microscopy
UEM: Ultrafast electron microscopy
UPS: Ultraviolet photoelectron spectroscopy
UV–Vis: Ultraviolet–visible spectroscopy
VAE: Variational autoencoder
VEELS: Valence electron energy-loss spectroscopy
XPS: X-ray photoelectron spectroscopy
ZLP: Zero-loss peak
